# Species diversity and geographical distribution of ticks infesting domestic animals in Bagmati Province, Nepal

**DOI:** 10.1371/journal.pone.0351151

**Published:** 2026-06-08

**Authors:** Kamana Thapa, Chet Raj Pathak, Somnath Aryal, Surendra Prasad Kanu

**Affiliations:** Faculty of Animal Science, Veterinary Science and Fisheries, Agriculture and Forestry University, Rampur, Chitwan, Nepal; Kerman University of Medical Sciences, IRAN, ISLAMIC REPUBLIC OF

## Abstract

**Background:**

Ticks are important vectors for bacterial, viral, rickettsial, and protozoal diseases in different animals and humans, especially in tropical countries. Tick-borne pathogens (TBPs) are host and vector-species-specific and depict geographical variations. This study was conducted to determine the diversity of ticks and their geographical distribution among domestic animals.

**Methodology/Principal Findings:**

Ticks were collected from a total of 210 domestic animals: cattle (n = 30), buffaloes (n = 40), sheep (n = 50), goats (n = 30), and dogs (n = 60), representing Chitwan (Terai), Kathmandu (mid-hill), and Rasuwa (high-hill) of Nepal. The genus and species of ticks were identified using morphological keys under a stereomicroscope. Analysis was performed using MS Excel, and Shannon-Wiener’s and Simpson’s diversity indexes were calculated. A total of 4 genera of hard ticks were observed, representing *Rhipicephalus*, *Haemaphysalis*, *Dermacentor*, and *Hyalomma*. Among them, *Rhipicephalus (Boophilus) microplus* (30.42%) was most abundant, followed by *Haemaphysalis longicornis* (26.74%), *Rhipicephalus sanguineus* (18.8%), *Haemaphysalis sulcata* (13.23%), and *Rhipicephalus decoloratus* (2.86%). Similarly, *Haemaphysalis spp.* (2.18%), *Haemaphysalis leachi* (2.05%), *Dermacentor sp.* (1.36%), *Rhipicephalus spp.* (1.36%), *Hyalomma sp.* (0.41%), and *Rhipicephalus parva* (0.14%). Geographical distribution and tick species diversity were higher in Chitwan. Similarly, species diversity in the case of the host was found to be greater in small ruminants. The result suggested higher tick distribution and diversity among warm tropical regions of Nepal.

**Conclusions:**

This study investigates the distribution of ticks (*Haemaphysalis*, *Rhipicephalus*, *Dermacentor*, and *Hyalomma*) in domestic animals across various climatic conditions, identifying the high-risk areas and animal species for tick-borne diseases, including zoonoses. The findings highlight the need for advanced research on tick ecology, epidemiology of TBPs, and the implementation of effective control strategies.

## Introduction

Ectoparasites constitute a significant problem for livestock and humans of most developing and underdeveloped countries worldwide. Different types of ectoparasites are observed in animals, like lice, ticks, fleas, and mites, hampering animal health as well as their production capabilities [1]. Ticks are considered one of the most common ectoparasites, affecting both animals and humans. Since ticks are hematophagus ectoparasites, they act as a potential vector for transmitting pathogens from infected to healthy humans and animals [2]. The nature of the transmission cycle and tick-borne pathogens (TBPs) depends on the species of tick involved. Ticks are found worldwide but are more prevalent in tropical and subtropical countries [3]. Approximately 900 tick species have been documented worldwide, of which more than 700 belong to the hard ticks (Ixodidae) [4].

Ticks cause significant economic loss in animals through anemia, reduction in weight, and milk production [5]. Tick-induced itching and local irritations have been shown to cause a 20–30% drop in the hide quality in cattle [6]. Ticks are considered the most important vectors in the case of animals, whereas they are considered the second most important vectors in humans for disease the transmission [7,8]. Ticks are responsible for the transmission of viral, bacterial, rickettsial, and parasitic pathogens [9–13]. However, in humans, the most common diseases transmitted by ticks are Lyme disease, ehrlichiosis, babesiosis, Colorado fever, Rocky Mountain fever, tularemia, Q fever, tick paralysis, and tick encephalitis [14–17].

In the context of Nepal, economic loss due to ticks and tick-borne diseases in livestock was reported to be 18.71% of the total livestock production cost [18]. The economic loss can be described as direct loss, loss due to the cost of treatment and management of ticks and tick-borne pathogens, and indirect loss, loss due to the retardation in the growth, production, and reproduction due to ill health, respectively. Similarly, in the case of dogs, the large proportion of community and stray dogs lacks basic health monitoring and treatment [19]. Those dogs are more likely to harbor ticks and different TBPs, which may become a source of infection for healthy humans [19]. Previous records regarding TBPs and their potential health impacts highlight the probability of diverse tick species in different geographical locations and animal hosts.

There are limited studies regarding tick species prevalence, abundance, species diversity, and geographic distribution of ticks infesting animals in Nepal [18]. Those limitations in systematic research on prevalence, abundance, and distribution are one of the main reasons for the failure to attain effective preventive measures against ticks in Nepal [6]. The environmental conditions and inadequate preventive and control strategies have offered an ideal setting for tick species in Nepal. Thus, the research was conducted as an attempt to identify ticks and observe the distribution pattern among different species of animals and geographical locations of Bagmati Province, Nepal.

## Materials and methods

### Study area

**Fig 1:**
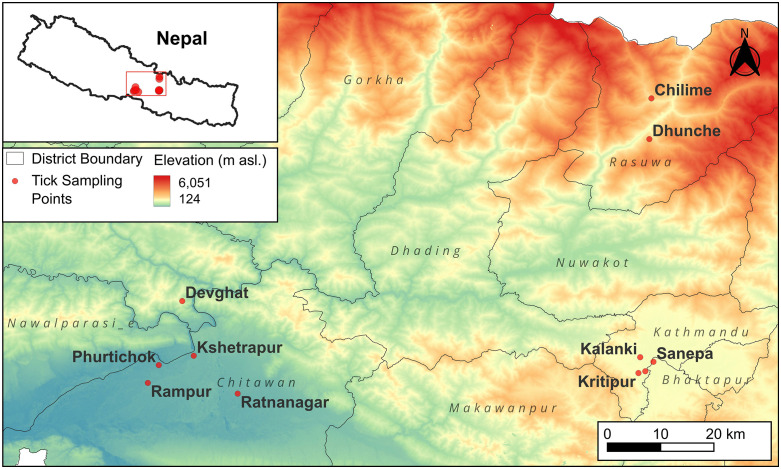
Different areas of study in Bagmati Province. Chitwan is located at an altitude of 301–1000 m and has subtropical to tropical climatic conditions with a temperature of 10°C–38°C, precipitation of 1,800 mm, and humidity of 75–80%. Similarly, Kathmandu is located at an elevation of 1,262 m–2,732 m above sea level, with a subtropical to temperate climatic zone with a temperature of 1°C–30°C, precipitation of 1,400 mm, and humidity of 65%. Rasuwa, the Himalayan region at an altitude of 300–5000 masl, ranges from subtropical to alpine with a temperature of −5°C – 20°C, precipitation of 1,200 mm, and humidity of 70–80%.

The study was carried out in Bagmati Province, which is in the central part of Nepal. Samples were collected from six different sampling municipalities: Kathmandu Metropolitan City and Kritipur Municipality, representing the Kathmandu District (mid-hill); Ratnanagar Municipality and Bharatpur Metropolitan City, representing Chitwan District (plain); and Dhunche Municipality and Aamachhodingmo Rural Municipality, representing Rasuwa (high-hill). Kathmandu and Chitwan were categorized as urban areas and Rasuwa a semi-urban/rural area. Ratnanagar, Rampur, Phurtichok, Khsetrapur, and Devghat were the sites from Chitwan District; Chovar, Sanepa, Kritipur, and Kalanki were the sampling sites in Kathmandu District; and Dhunche and Chilime were from Rasuwa District (Fig 1).

### Study duration and population

Ticks were collected directly from five different domestic animal species, including buffalo, cattle, sheep, goats, and dogs. The study was carried out from September 2023 to January 2024, and a total of 210 animals were included. Ethical approval for the study was obtained from the Department of Microbiology and Parasitology, and informed verbal consent was obtained from animal owners.

### Sample collection

The ticks were carefully removed using forceps and gloved hands. During the detachment of the tick from the animal's body, the basis capitulum of the tick was held to ensure that the mouth part remained intact. While collecting ticks from animals, the entire body was examined, particularly the ear, dewlap, perineal region, scrotum, udder, back, and inside the tuft of the tail, and ticks were collected from each animal as described in previous studies [20,21]. The collected ticks from different animal species were kept in separate labeled vials containing 70% ethanol and were stored at 4℃. Detailed information regarding the animals, such as age, sex, date of collection, severity of infestation, and locations, was also recorded.

### Identification of ticks

The collected ticks were brought to the laboratory, where tick genera were identified using a stereo-zoom microscope as dry method. Further species-level identification was performed at magnification of 40X and 100X. For the identification, different key morphological features of ticks, such as scutum, basis capitula, palp, dentition, presence or absence of spur, adanal or accessory plates, and festoon, were observed based on the published guidelines [22–24]. If the number of ticks identified up to the genus level contains more than one morphospecies of ticks, then we have used the term “spp.” considering their possibility of being multiple species under the common genera, whereas if the identified genera contain a single unidentified morphospecies, then we used the term “sp.” [25]. Only adult ticks were used in statistical analysis.

### Data analysis

Data entry and analysis of all variables and numerical data were conducted using MS Excel 2016. The data were analyzed quantitatively with a focus on species diversity, which implies species richness and evenness. Ticks identified up to the species level and genera containing single unidentified morphospecies were used for diversity index calculation.


$$\begin{array}{c} 𝐒𝐡𝐚𝐧𝐧𝐨𝐧-𝐖𝐢𝐞𝐧𝐞𝐫\;𝐝𝐢𝐯𝐞𝐫𝐬𝐢𝐭𝐲\;𝐢𝐧𝐝𝐞𝐱\;(𝐇)=-\;\sum \;{𝐩}_{𝐢}\;𝐥𝐧({𝐩}_{𝐢}) \end{array}$$

Where p_i_ proportion of each tick species.

A higher **H** value showed more diversity (many species and/or more even distribution), and a lower **H** value showed less diversity (few species or dominance by one species).


$$\begin{array}{c} 𝐒𝐢𝐦𝐩𝐬𝐨𝐧\text{'}𝐬\;𝐝𝐢𝐯𝐞𝐫𝐬𝐢𝐭𝐲\;𝐢𝐧𝐝𝐞𝐱\;(𝐒)=\sum \;𝐧(𝐧-1)/𝐍(𝐍-1) \end{array}$$

Where n = number of individuals of that single species

N = No. of individuals of the total population.

Simpson diversity index provides more emphasis on the dominant species [26,27]

Additionally, the Simpson diversity index and Shannon-Wiener diversity index were calculated for each sampling district (region), and animal categories [small ruminants (sheep and goats), large ruminants (cattle and buffalo), and pets (dogs)], as well as for the entire province using the formulas.

## Results

### Sample description

Approximately, 2–5 ticks were collected from each animal. A total of 755 ticks were collected from 210 animals in Bagmati Province. Among these animals, there were 50 sheep, 30 goats, 30 cattle, 40 buffaloes, and 60 dogs as mentioned in the S1 Table. Animals were categorized into the three age groups to make inferences for epidemiological relevance and susceptibility to tick infestations, i.e., < 1 yr, 1–3 yrs, or >3 yrs. In addition, the severity of tick infestations was also categorized as low (if <10 ticks/animal), moderate (10–50 ticks/animal), and high (>50 ticks/animal), as described previously in Haji et al. (2022) [28].

The number of male sheep was 6 (12%), compared to 44 female sheep (88%). For the goats, there were 13 males (43.33%) and 17 females (56.66%). All cattle were female, while 9 males (22.50%) and 26 females (77.50%) were found among the buffalo. The pet dogs were composed of 48 females (80%) and 12 males (20%). Most of the sheep and dogs in the study were aged 1–3 years, while the goats were less than 1 year old, and the cattle and buffalo were older than 3 years. During the sampling, most cattle, sheep, and goats had moderate tick infestations, whereas buffalo and dogs showed high levels of infestation (Table 1).

**Table 1:** Descriptive information on age, sex, and tick infestation severity on the host.

Animal	Sex		Age			Tick infestation severity		
	Male	Female	<1yr	1–3 yrs.	>3yrs	Less	Moderate	High
Sheep	6	44	11	26	13	8	22	20
Goat	13	17	12	11	7	5	18	7
Cattle	–	30	3	4	23	6	15	9
Buffalo	9	31	5	16	19	10	9	21
Dog	12	48	8	28	24	4	22	34
Total	40	170	39	85	86	33	86	91

There were 755 ticks in total, 17 (2.25%) of which were nymphs and 738 (97.75%) adults. Sexual dimorphism and identification of only adults were possible; however, only 5 ticks were classed as unidentified ticks. Male ticks accounted for 38.60% and female ticks for 61.3% of the total adult tick population, and the sex ratio of each species of tick genus was calculated (Table 2).

**Table 2:** Sex ratio of the tick population on domestic animals of the Bagmati province.

Tick species	Male tick count	Female tick count	Sex ratio (female: male)	Sex ratio of the genus
*Rhipicephalus microplus*	86	137	1.59:1	1.14:1
*Rhipicephalus decoloratus*	15	6	0.40:1	
*Rhipicephalus sanguineus*	81	57	0.70:1	
*Rhipicephalus parva*	1	–	–	
*Rhipicephalus* spp.	–	10	–	
*Haemaphysalis sulcata*	–	97	–	2.52:1
*Haemaphysalis longicornis*	92	104	1.13:1	
*Haemaphysalis leachi*	–	15	–	
*Haemaphysalis* spp.	–	16	–	
*Dermacentor* **sp*.***	8	5	0.62:1	0.62:1
*Hyalomma* **sp*.***	–	3	–	–
**Total**	**283**	**450**		

### Tick species identification and abundance

**Fig 2:**
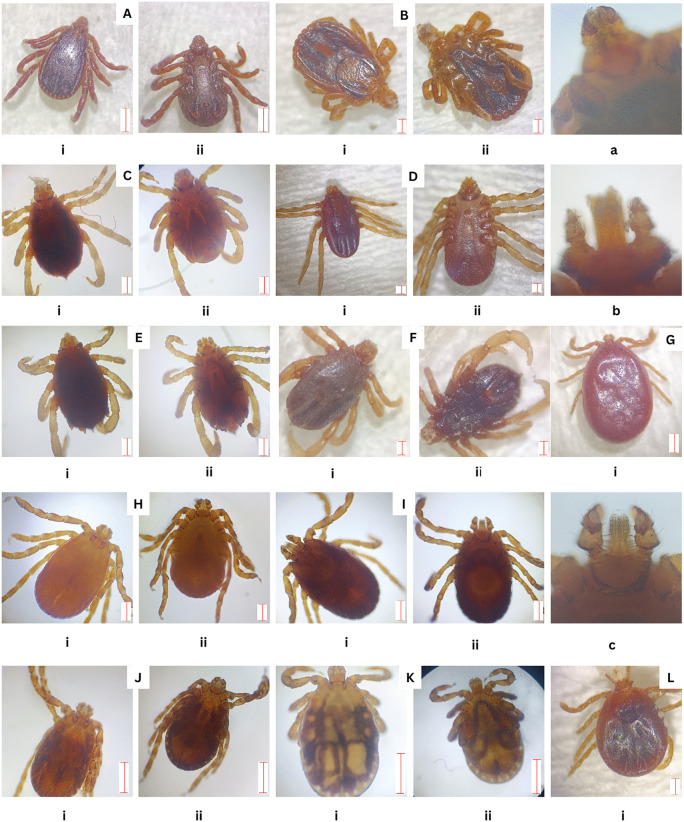
Morphology of different species of ticks. **A.** R*hipicehalus sanguineus* male: 2.3–3.1 mm; **B**. *R. sanguineus* female: 4.0–5.0 mm; **a**. mouthpart of *Rhipicehalus sanguineus;* **C**. *R. microplus* male: 1.0–1.5 mm; **D**. *R. microplus* female: 4.5–8.0 mm; **b**. Mouthpart of *R. microplus;* **E**. *R. decoloratus* male: 2.3–2.4 mm; **F**. *R. parva* male: 3.5–5.0 mm; **G**. *Haemaphysalis sulcate* female: 2.7–3.5 mm; **H**. *H. longicornis* male: 2.40–2.50 mm; **I**. *H. longicornis* female: 2.0–3.4 mm; **c**. Mouthpart of *H. longicornis;* **J**. *H. leachi* male: 1.5–1.8 mm; **K**. *Dermacentor* sp*.* male: 2.5–4.0 mm; **L**. *Hyalomma* sp*.* female: 3.5–6.0 mm. (Scale bar: 1 mm for total body length of ticks).

Ticks collected were identified to genus and species level, except nymphs and 5 unidentified ticks. During the study, 4 genera of ticks were identified (Fig 2). Among them, two genera were identified to the species level and the remainder to the genus level only. These include ticks like *Rhipicephalus* [*Rhipicephalus (Boophilus*) *microplus, Rhipicephalus (Boophilus) decoloratus, Rhipicephalus parva, Rhipicephalus sanguineus, Rhipicephalus* spp.], *Haemaphysalis* (*Haemaphysalis longicornis, Haemaphysalis sulcata, Haemaphysalis leachi, Haemaphysalis* spp*.), Dermacentor* sp*.,* and *Hyalomma* sp*.* During identification, the main morphological features of ticks, like scutum, palp, basis capitula, dentition, festoon, coxae, and spur, were detected (Fig 2).

In the current study, *Rhipicephalus* had the highest prevalence (53%) in domestic animals. *Rhipicephalus microplus* was found to be the most abundant species with 30.42%, followed by *Haemaphysalis longicornis* with 26.74%, *Rhipicephalus sanguineus* with 18.83%, *Haemaphysalis sulcata* with 13.23%, *Rhipicephalus decoloratus* with 2.86%, and *Haemaphysalis* spp. 2.18%, *Haemaphysalis leachi* 2.05%, *Dermacentor* sp*.* 1.77%, *Rhipicephalus* spp. 1.36%, *Hyalomma* sp*.* 0.41% and *Rhipicephalus parva* 0.14% (Table 3).

**Table 3:** The abundance of different identified tick species in domestic animals.

Tick Species	Count	Abundance (%)
*Rhipicephalus microplus*	233	30.42
*Haemaphysalis longicornis*	196	26.74
*Rhipicephalus sanguineus*	138	18.83
*Haemaphysalis sulcata*	97	13.23
*Rhipicephalus decoloratus*	21	2.86
*Haemaphysalis leachi*	15	2.05
*Haemaphysalis* spp.	16	2.18
*Dermacentor* sp.	13	1.77
*Rhipicephalus* spp.	10	1.36
*Hyalomma* sp.	3	0.41
*Rhipicephalus parva*	1	0.14
**Total**	**733**	**100**

### Host-wise tick distribution

*Rhipicephalus microplus* was found in all four species of domestic ruminants, whereas *Rhipicephalus decoloratus* was found only in large ruminants. Moreover, the goats and sheep of Bagmati province were mostly observed to be affected by *Haemaphysalis longicornis* (59.21%) and then by *Haemaphysalis sulcata* (31.5%). Large ruminants and dogs were less infested by these tick species. Mostly, *Rhipicephalus sanguineus* (76.6%) was observed in dog species and was not seen in domestic ruminants. Also, *Hyalomma* ticks were noted in sheep, and most of the *Dermacentor* sp*.* (76.92%) were found in goats. The host-wise distribution of ticks is presented in Table 4.

**Table 4:** Host-wise distribution of ticks.

Tick species	Tick number				
	Cattle	Buffalo	Sheep	Goat	Dog
*Rhipicephalus microplus*	78	142	2	1	0
*Rhipicephalus decoloratus*	7	14	0	0	0
*Rhipicephalus sanguineus*	0	0	0	0	138
*Rhipicephalus* spp.	0	0	0	0	10
*Rhipicephalus parva*	0	1	0	0	0
*Haemaphysalis sulcata*	1	0	75	21	0
*Haemaphysalis longicornis*	5	0	126	54	11
*Haemaphysalis leachi*	0	0	0	0	15
*Haemaphysalis* spp.	1	0	8	3	4
*Dermacentor* **sp*.***	0	0	1	10	2
*Hyalomma* **sp*.***	0	0	3	0	0
**Total**	**92**	**157**	**215**	**89**	**180**

### Geographical distribution of ticks and diversity

More ticks were observed from Chitwan (90.72%) compared to Rasuwa (2.72%) and Kathmandu (6.54%) (Table 5). Total abundance and richness of tick species were observed to be higher in Chitwan (90.72%) or plain terai compared to hill and high hill. Similarly, *Rhipicephalus microplus* was found to be the most abundant tick in Chitwan (29.77%), followed by *Haemaphysalis longicornis* (28.57%), and the least abundant was *Rhipicephalus parva* (0.15%). *Rhipicephalus microplus* ticks from Kathmandu also showed the highest abundance (52.08%), whereas in Rasuwa, *Rhipicephalus sanguineus* (50%) and *Dermacentor* sp*.* (50%) were observed in equal proportion.

**Table 5:** Geographical distribution of ticks in different locations.

Tick species	Abundances of tick species			Total abundance (%)
	Chitwan	Kathmandu	Rasuwa	
*Rhipicephalus microplus*	198	25	–	223 (30.42)
*Rhipicephalus decoloratus*	21	–	–	21 (2.86)
*Rhipicephalus sanguineus*	113	15	10	138 (18.83)
*Rhipicephalus parva*	1	–	–	1 (0.14)
*Rhipicephalus* spp.	10	–		10 (1.36)
*Haemaphysalis sulcata*	97	–		97 (13.23)
*Haemaphysalis longicornis*	190	6		196 (26.74)
*Haemaphysalis leachi*	15	–		15 (2.05)
*Haemaphysalis* spp.	14	2		16 (2.18)
*Dermacentor* sp***.***	3	–	10	13 (1.77)
*Hyalomma* sp*.*	3	–	–	3 (0.40)
**Total**	665	48	20	733

### Species richness and diversity

In Bagmati province species’ richness was found to be higher in the Terai (9 species), followed by mid-hill (3 spp.) and high hill/mountain (2 spp.) (Table 6). In the case of the Shannon-Wiener diversity index, the values calculated were 1.57, 0.96, and 0.69, respectively, from Terai, mid-hill, and high hill. Among these regions, the Terai was found to have high diversity, and the high hills had low diversity. Moreover, the Simpson indices for various species of ticks in three geographical regions were 0.24 for plain, 0.41 for mid-hill, and 0.47 for high hill. This also showed the highest diversity in plains, as the lower the value of Simpson’s index, the higher the diversity.

**Table 6:** Calculation of tick species diversity among different areas and hosts.

Parameters	Bagmati Province	Terai	Mid-hill	High hill	Large Ruminants	Small Ruminants	Dogs
**Species richness**	9	9	3	2	6	5	4
**Shannon Wiener's diversity index**	1.59	1.57	0.96	0.69	0.44	0.88	0.60
**Simpson’s Diversity Index**	0.24	0.24	0.41	0.47	0.79	0.49	0.70

Among large ruminants, small ruminants, and pets, a total of 6 species, 5 species, and 4 species of ticks were identified respectively, i.e., all animal species were infested by the number of ticks. The Shannon Wiener diversity index was higher for small ruminants (0.88), followed by dogs (0.60), and large ruminants (0.44) (Table 6). This showed more diversity of ticks among small ruminants and less diversity in large ruminants. In addition, Simpson’s diversity index values for large ruminants (0.79), small ruminants (0.49), and dogs (0.70) indicate higher diversity in ticks among small ruminants. The overall value of the Shannon-Wiener diversity index of ticks among domestic animals of Bagmati province was 1.59, and Simpson’s diversity index was 0.24.

## Discussion

Previous studies in ticks and tick-borne pathogens in Nepal detected *Rhipicephalus, Haemaphysalis, Dermacentor* ticks and pathogens like *Babesia vogeli, Babesia gibsoni*, *Ehrlichia canis*, *Hepatozoon canis*, *Anaplasma platys* in dogs [19,21,29]. However, in the case of cattle, *Rhipicephalus microplus, Haemaphysalis, Ixodes, Amblyomma* ticks and *Anaplasma bovis, Anaplasma marginale*, *Theileria annulata, Theileria orientalis* pathogens were observed [30–33]. Limited studies can be found in case of tick-borne pathogens in goats, diversity, and distribution of ticks among different animals and different geographical regions.

In this study, four genera of ticks (*Rhipicephalus*, *Haemaphysalis*, *Dermacentor*, and *Hyalomma*) were identified among the domestic animals and pets of different geographical regions of central Nepal. Among the four genera, *Rhipicephalus* had four species (*R. microplus*, *R. decoloratus*, *R*. *sanguineus*, *R. parva*), and *Haemaphysalis* had three species (*H. sulcata*, *H. longicornis*, *H. leachi*); however, the two genera, *Dermacentor* and *Hyalomma*, were not fully identified up to the species level based on the morphological characteristics.

The prevalence and host associations of various tick species in this study showed distinct patterns across the different livestock and companion animals. The current study revealed the prevalence of three genera of ticks in goats, including *Rhipicephalus*, *Haemaphysalis*, and *Dermacentor,* similar to those of the study from Kunwar A. et al. (2022) [18]. The presence of more Ixodids in goats could be because of the sampling duration, as this study was conducted mainly in the winter period (unfavorable conditions) [21,34]. These ticks are responsible for transmitting *Anaplasma*, *Babesia*, and *Theileria* in goats [35]. Similarly, *H. longicornis* was the most abundant and predominant species of tick infesting sheep, which is supported by a study in China [36]; however, it contrasts with findings from Iran [37], which showed *R. sanguineus* as the dominant tick species. These may be due to geographical differences, grazing patterns, and sampling sites in the small ruminants, like the ear and head [38]. *H. longicornis* is mainly responsible for transmitting *Rickettsia*, *Anaplasma*, and *Borrelia* in sheep and can potentially infect humans, also contributing to the transmission of Japanese spotted fever (*Rickettsia japonica*), Anaplasmosis (*Anaplasma* spp.), and Lyme disease (*Borrelia* spp.) [36]. One study in the Siraha district of Nepal revealed the prevalence of *Babesia* and *Anaplasma* in goats [39], but the prevalence of tick-borne diseases in goats is still lacking in the study areas. Therefore, the study highlights the prevalence and distribution of various tick species in small ruminants, emphasizing their role in pathogen transmission and the potential health risks among animals and humans.

Tick infestation patterns in cattle and buffaloes revealed the predominance of a single species, with important implications for disease transmission. *Rhipicephalus microplus* was the most abundant among the cattle, which is supported by previous studies [32,33,40]. *R. microplus* is responsible for transmitting *Rickettsia* spp., *Anaplasma* spp., and *Ehrlichia* spp. in cattle [41]. However, ticks like *Ixodes and Amblyomma* were observed in Chitwan in a previous study [32], which were not detected in this study. Similarly, the predominant tick infesting buffaloes was *Rhipicephalus microplus,* which agrees with the findings from Uttar Pradesh, India [42]. Thus*, R. microplus* is observed as the dominant tick species in both cattle and buffaloes, highlighting its possible role in the transmission of important pathogens in Central Nepal [33].

In the case of a dog, a high prevalence of *Rhipicephalus sanguineus* was observed. *R. sanguineus* plays an important role in the transmission of *Anaplasma* spp., *Ehrlichia* spp., and *Babesia* spp. in dogs [29]. All these findings were supported by different previous studies in Nepal and India [6,40] Therefore, in dogs, *R. sanguineus* was highly prevalent and recognized as a major vector of several important pathogens.

In addition to species prevalence and pathogen transmission, the sex ratio *within* tick populations further influences their reproductive success and associated health risks to domestic animals. The analysis of tick populations on domestic animals revealed notable variations in sex ratios, which have important implications for reproduction, pathogen transmission, and host health [43,44]. The sex ratio of the tick (female: male) population on domestic animals showed that *Rhipicephalus* spp. and *Haemaphysalis* spp. have a greater proportion of female ticks, but lower in the case of *Dermacento*r. These findings are similar to a study in Lebanon [45]; however, they are somewhat in contrast with that of Iran [14], which showed that the ratio of females to males was lower. Moreover, a sex ratio greater than one may lead to a higher reproductive potential among the genera of *Rhipicephalus* spp. and *Haemaphysalis* spp., in turn, increasing the risk of TBPs [43]. Conversely, in the case of *Dermacentor* sp*.*, males were in high proportion, reflecting the lower risks of TBP transmission and blood loss [44,46]. Therefore, a sex ratio favoring females in a tick population implies a greater potential than male ticks for reproduction, pathogen transmission, and economic impact on the host animals.

Species richness was notably highest in the Terai, declining sharply in the mid-hills and high hills/mountains. Most of the ticks used in the study were from the Terai region, and a smaller sample was collected from the hills and Himalayas, which may be due to the tropical and sub-tropical climate in the Terai and geographical differences, as well as the low prevalence of ticks in winter or cold areas. This is in conjunction with previous study of ticks in mid hills of Nepal [6], which also reported the highest number of ticks in Chitwan compared to Dang and Lamjung. This trend is consistent with ecological expectations, where lower elevations generally offer more favorable environmental conditions (like higher temperature, humidity, and vegetation cover) to enhance the greater biodiversity of ticks [47]. Along with the species’ richness, the tick diversity was also higher in the case of the Terai than in the hill and the high hill/mountain. This might be due to the greater number of domestic hosts involved in the study in Terai. This indicates that in the Terai regions, the probability of getting infected with TBPs is higher in animals. Higher species richness in plain areas increases the risk of transmitting a wider range of TBPs (*Babesia, Anaplasma, Theileria,* and *Ehrlichia)* to animals and humans, complicating disease control efforts [29]. Therefore, areas with high tick diversity require more comprehensive surveillance and management strategies to mitigate health and economic impacts.

Not all the ticks collected were fully identified because of the presence of different life stages of ticks, like nymphs and larvae, where identification based on key morphological features is complex. The reason might be due to variation in ornamentation among different geographical regions, and under the limitations of resources for confirmation. Some highly engorged females, which lacked key morphological features, were kept as unidentified ticks. Similarly, some of the engorged females of *Rhipicephalus* and *Haemaphysalis* were only identified up to the genus level due to a lack of visible morphological features to classify them up to the species level. Out of the total ticks in this study, five of them were designated as unidentified ticks. This was best explained by the loss of mouth parts during the collection of ticks and the absence of major identifying features. Thus, morphology-based identification of ticks depends on proper sample collection, preservation, and careful observation using standard protocols for tick collection, including the flagging-dragging, carbon dioxide traps, or host-based collection [48].

In the future, researchers should focus on molecular identification of tick species in any form (engorged, morphologically damaged/unidentified, and cryptic species) and pathogens associated with them to better understand disease risks. Moreover, long-term ecological studies and integrated tick management strategies are essential to predict changes in tick distribution and mitigate the rising threat of TBPs under environmental and climate change scenarios.

### Constraints of the study

Major constraint of this study was sampling, and it was performed during the winter, not an ideal season for ticks. Hence, study during this short period may underestimate the tick species collected and identified. Molecular confirmation was not done due to limited resources, and it is solely based on morphology, providing room for some technical errors. Health impact and TBPs of animal and public health were not included due to time and resource constraints.

## Conclusions

Ticks are neglected arthropods, especially in the South Asian region, with huge importance in veterinary and public health systems. This study showed four genera of ticks prevalent in different domestic animals and in three districts of Bagmati Province, ranging from plain to high hill, which include *Rhipicephalus, Haemaphysalis, Dermacentor*, and *Hyalomma*. The tick distribution and diversity are higher in Chitwan as compared to other areas, which may increase the risks of transmission of various TBPs. Moreover, some of the ticks have zoonotic importance that needs to be further explored with priority. A proper integrated tick management system should be used to safeguard the health of animals and the public from TBPs, especially in the areas of high tick diversity.

## Supporting information

S1 Table Distribution of host from which ticks were collected from the study area. This includes the number of different species of host involved in the study from different regions of Bagmati, Province.
(DOCX)
