# Supplementary material for: Species diversity and geographical distribution of ticks infesting domestic animals in Bagmati Province, Nepal
Source: PLoS One. 2026 Jun 8;21(6):e0351151. doi: 10.1371/journal.pone.0351151 (PMC13245791; doi:10.1371/journal.pone.0351151)
Supplement: S1 Table — This includes the number of different species of host involved in the study from different regions of Bagmati, Province. (DOCX) [file pone.0351151.s001.docx]

**S1 Table**. Distribution of host from which ticks were collected from the study area

| **Host** | **Terai** | **Midhill** | **Mountain** |
| --- | --- | --- | --- |
| Cattle | 28 | 2 | 0 |
| Buffalo | 22 | 18 | 0 |
| Sheep | 45 | 3 | 1 |
| Goat | 20 | 4 | 6 |
| Dog | 41 | 12 | 7 |
| **Total** | **156** | **39** | **14** |
