# Supplementary material for: Species diversity and geographical distribution of ticks infesting domestic animals in Bagmati Province, Nepal
Source: PLoS One. 2026 Jun 8;21(6):e0351151. doi: 10.1371/journal.pone.0351151 (PMC13245791; doi:10.1371/journal.pone.0351151)

PLOS ONE

# Species diversity and geographical distribution of ticks infesting domestic animals in Bagmati province, Nepal

--Manuscript Draft--

| | |
|---|---|
| Manuscript Number: | PONE-D-25-27830 |
| Article Type: | Research Article |
| Full Title: | Species diversity and geographical distribution of ticks infesting domestic animals in Bagmati province, Nepal |
| Short Title: | Distribution of ticks in domestic animals across ecological zones of Bagmati,Nepal |
| Corresponding Author: | Kamana Thapa<br>Agriculture and Forestry University<br>Chitwan, NEPAL |
| Keywords: | Dogs, Ixodids, Morphometry, Ruminants, Tick diversity |
| Abstract: | Background Ticks are hematophagous arthropods that are vectors for bacterial, viral, rickettsial, and protozoal diseases in different animals and humans, especially in tropical countries. Tick-borne pathogens (TBPs) are host and vector-species-specific and depict geographical variations. This study was conducted to determine the diversity of ticks and their geographical distribution among domestic animals. Ticks were collected from a total of 210 domestic animals, cattle (n = 30), buffaloes (n = 40), sheep (n = 50), goats (n = 30), and dogs (n = 60) representing Chitwan (terai), Kathmandu (mid-hill), and Rasuwa (high hill). The genus and species of ticks were identified using morphological keys under a stereomicroscope. Analysis was performed using MS Excel, and Shannon-Wiener's and Simpson's diversity indexes were calculated. Methodology/Principal Findings A total of 4 genera of ticks were observed, representing Rhipicephalus, Haemaphysalis, Dermacentor, and Hyalomma. Among them, Rhipicephalus (Boophilus) microplus (30.42%) was most abundant, followed by Haemaphysalis longicornis (26.74%), Rhipicephalus sanguineus (18.8%), Haemaphysalis sulcata (13.23%), and Rhipicephalus decoloratus (2.86%). Similarly, Haemaphysalis spp. (2.18%), Haemaphysalis leachi (2.05%), Dermacentor spp. (1.36%), Rhipicephalus spp. (1.36%), Hyalomma spp. (0.41%), and Rhipicephalus parva (14%). Geographical distribution and tick species diversity were higher in Chitwan. Similarly, species diversity in the case of the host was found to be greater in small ruminants. The result suggested higher tick distribution and diversity among warm tropical regions of Nepal Conclusions This study investigates the distribution of ticks (Haemaphysalis, Rhipicephalus, Dermacenter, Hyalomma) in domestic animals across various climatic conditions, identifying the high-risk areas and animal species for tick- borne diseases, including zoonoses. The findings highlight the need for advanced research on tick ecology, epidemiology of TBPs, and the implementation of effective control strategies. |
| Order of Authors: | Kamana Thapa |
| | Chet Raj Pathak |
| | Somnath Aryal |
| | Surendra Prasad Kanu |
| Additional Information: | |
| Question | Response |
| **Financial Disclosure**<br><br>Enter a financial disclosure statement that describes the sources of funding for the work included in this submission. Review the submission guidelines for detailed requirements. View published research | The author(s) received no specific funding for this work. |

| | |
|---|---|
| articles from *PLOS ONE* for specific examples.<br><br>This statement is required for submission and **will appear in the published article** if the submission is accepted. Please make sure it is accurate.<br><br>**Funded studies**<br>Enter a statement with the following details:<br>• Initials of the authors who received each award<br>• Grant numbers awarded to each author<br>• The full name of each funder<br>• URL of each funder website<br>• Did the sponsors or funders play any role in the study design, data collection and analysis, decision to publish, or preparation of the manuscript?<br><br>Did you receive funding for this work? | |
| **Competing Interests**<br><br>Use the instructions below to enter a competing interest statement for this submission. On behalf of all authors, disclose any competing interests that could be perceived to bias this work—acknowledging all financial support and any other relevant financial or non-financial competing interests.<br><br>This statement is required for submission and **will appear in the published article** if the submission is accepted. Please make sure it is accurate and that any funding sources listed in your Funding Information later in the submission form are also declared in your Financial Disclosure statement.<br><br>View published research articles from *PLOS ONE* for specific examples. | The authors have declared that no competing interests exist. |

| | |
|---|---|
| **NO authors have competing interests**<br><br>Enter: *The authors have declared that no competing interests exist.*<br><br>**Authors with competing interests**<br><br>Enter competing interest details beginning with this statement:<br><br>*I have read the journal's policy and the authors of this manuscript have the following competing interests: [insert competing interests here]*<br><br>* typeset | |
| **Ethics Statement**<br><br>Enter an ethics statement for this submission. This statement is required if the study involved:<br><br>• Human participants<br>• Human specimens or tissue<br>• Vertebrate animals or cephalopods<br>• Vertebrate embryos or tissues<br>• Field research<br><br>Write "N/A" if the submission does not require an ethics statement.<br><br>General guidance is provided below. Consult the submission guidelines for detailed instructions. **Make sure that all information entered here is included in the Methods section of the manuscript.** | N/A |

*Powered by Editorial Manager® and ProduXion Manager® from Aries Systems Corporation*

| | |
|---|---|
| **Format for specific study types**<br><br>**Human Subject Research (involving human participants and/or tissue)**<br>• Give the name of the institutional review board or ethics committee that approved the study<br>• Include the approval number and/or a statement indicating approval of this research<br>• Indicate the form of consent obtained (written/oral) or the reason that consent was not obtained (e.g. the data were analyzed anonymously)<br><br>**Animal Research (involving vertebrate animals, embryos or tissues)**<br>• Provide the name of the Institutional Animal Care and Use Committee (IACUC) or other relevant ethics board that reviewed the study protocol, and indicate whether they approved this research or granted a formal waiver of ethical approval<br>• Include an approval number if one was obtained<br>• If the study involved *non-human primates*, add *additional details* about animal welfare and steps taken to ameliorate suffering<br>• If anesthesia, euthanasia, or any kind of animal sacrifice is part of the study, include briefly which substances and/or methods were applied<br><br>**Field Research**<br><br>Include the following details if this study involves the collection of plant, animal, or other materials from a natural setting:<br>• Field permit number<br>• Name of the institution or relevant body that granted permission | |
| **Data Availability**<br><br>Authors are required to make all data underlying the findings described fully available, without restriction, and from the time of publication. PLOS allows rare exceptions to address legal and ethical concerns. See the PLOS Data Policy and FAQ for detailed information. | Yes - all data are fully available without restriction |

| | |
|---|---|
| A Data Availability Statement describing where the data can be found is required at submission. Your answers to this question constitute the Data Availability Statement and **will be published in the article**, if accepted.<br><br>**Important:** Stating 'data available on request from the author' is not sufficient. If your data are only available upon request, select 'No' for the first question and explain your exceptional situation in the text box.<br><br>Do the authors confirm that all data underlying the findings described in their manuscript are fully available without restriction? | |
| **Describe where the data may be found in full sentences. If you are copying our sample text, replace any instances of XXX with the appropriate details.**<br><br>• If the data are **held or will be held in a public repository**, include URLs, accession numbers or DOIs. If this information will only be available after acceptance, indicate this by ticking the box below. For example: *All XXX files are available from the XXX database (accession number(s) XXX, XXX.)*.<br>• If the data are all contained **within the manuscript and/or Supporting Information files**, enter the following: *All relevant data are within the manuscript and its Supporting Information files.*<br>• If neither of these applies but you are able to provide **details of access elsewhere**, with or without limitations, please do so. For example:<br><br>*Data cannot be shared publicly because of [XXX]. Data are available from the XXX Institutional Data Access / Ethics Committee (contact via XXX) for researchers who meet the criteria for access to confidential data.*<br><br>*The data underlying the results presented in the study are available from (include the name of the third party* | All relevant data are within the manuscript |

| | |
|---|---|
| *and contact information or URL).*<br>• This text is appropriate if the data are owned by a third party and authors do not have permission to share the data.<br><br>* typeset | |
| Additional data availability information: | |

Manuscript

Click here to access/download;Manuscript;Tick_Diversity_Nepal_Manuscript

# Species diversity and geographical distribution of ticks infesting domestic animals in Bagmati province, Nepal

Kamana Thapa[a*], Chet Raj Pathak[a*], Somnath Aryal[a], Surendra Prasad Kanu[a]

[a] Faculty of Animal Science, Veterinary Science and Fisheries, Agriculture and Forestry University, Rampur, Chitwan 44209, Nepal

*Correspondences:

Kamana Thapa; Faculty of Animal Science, Veterinary Science and Fisheries, Agriculture and Forestry University, Rampur, Chitwan 44209, Nepal

Email: kamana.thapa@favf.edu.np; Phone: +977 9845902733

Assistant Professor Chet Raj Pathak; Faculty of Animal Science, Veterinary Science and Fisheries, Agriculture and Forestry University, Rampur, Nepal

Email: crpathak@afu.edu.np; Phone: +977 9848827681

## ABSTRACT

**Background:** Ticks are hematophagous arthropods that are vectors for bacterial, viral, rickettsial, and protozoal diseases in different animals and humans, especially in tropical countries. Tick-borne pathogens (TBPs) are host and vector-species-specific and depict geographical variations. This study was conducted to determine the diversity of ticks and their geographical distribution among domestic animals. Ticks were collected from a total of 210 domestic animals, cattle (n = 30), buffaloes (n = 40), sheep (n = 50), goats (n = 30), and dogs (n = 60) representing Chitwan (terai), Kathmandu (mid-hill), and Rasuwa (high hill). The genus and species of ticks were identified using morphological keys under a stereomicroscope. Analysis was performed using MS Excel, and Shannon-Wiener's and Simpson's diversity indexes were calculated.

**Methodology/Principal Findings:** A total of 4 genera of ticks were observed, representing *Rhipicephalus*, *Haemaphysalis*, *Dermacentor*, and *Hyalomma*. Among them, *Rhipicephalus* (*Boophilus*) *microplus* (30.42%) was most abundant, followed by *Haemaphysalis longicornis* (26.74%), *Rhipicephalus sanguineus* (18.8%), *Haemaphysalis sulcata* (13.23%), and *Rhipicephalus decoloratus* (2.86%). Similarly, *Haemaphysalis* spp. (2.18%), *Haemaphysalis leachi* (2.05%), *Dermacentor* spp. (1.36%), *Rhipicephalus* spp. (1.36%), *Hyalomma* spp. (0.41%), and *Rhipicephalus parv*a (14%). Geographical distribution and tick species diversity were higher in Chitwan. Similarly, species diversity in the case of the host was found to be greater in small ruminants. The result suggested higher tick distribution and diversity among warm tropical regions of Nepal

**Conclusions:** This study investigates the distribution of ticks (*Haemaphysalis*, *Rhipicephalus*, *Dermacenter*, *Hyalomma*) in domestic animals across various climatic conditions, identifying the high-risk areas and animal species for tick- borne diseases, including zoonoses. The findings highlight the need for advanced research on tick ecology, epidemiology of TBPs, and the implementation of effective control strategies.

## Author Summary

Ticks are ~~the~~ important vectors of various tick-borne diseases that affect both animals and humans. Our study investigates diversity and geographical distribution of tick species among domestic animals in Bagmati Province, focusing on cattle, buffaloes, sheep, goats and dogs and three different regions (Chitwan, Kathmandu, Rasuwa). A total of four tick genera were identified – Rhipicephalus, Haemaphysalis, Dermacenter, and Hyalomma with Rhipicephalus (Boophilus) microplus being the most abundant species. Tick species diversity and distribution were found to be higher in tropical regions and small ruminants. Our findings help identify the animals and regions most at risk, highlighting the importance of targeted surveillance and control strategies. We emphasize the need for further research into tick ecology, epidemiology of TBPs, and control measures to mitigate the risks of tick-borne diseases in Nepal.

## 1. Introduction

Ectoparasites constitute a significant problem in most developing and underdeveloped countries worldwide. Different types of ectoparasites are observed in animals, like lice, ticks, fleas, and mites, hampering the health as well as the production of animals [1]. Ticks are considered one of the most common ectoparasites, affecting animals and humans. Since ticks are blood-sucking ectoparasites, they act as a potential vector for transmitting pathogens from infected to healthy

humans and animals [2]. The nature of the transmission cycle and tick-borne pathogens (TBPs) depends on the species of tick involved. Ticks are found worldwide but are more prevalent in tropical and subtropical countries [3]. Approximately 900 tick species have been documented worldwide, of which more than 700 belong to the hard ticks (Ixodidae)[4].

Ticks cause significant economic loss in animals through anemia, reduction in weight, and milk production [5]. Tick-induced itching and local irritations have been shown to cause a 20–30% drop in the hide in cattle [6]. Ticks are the first important vector in humans, and in the case of animals, they hold the second position as an essential vector for disease transmission [7]. They transmit viral, bacterial, rickettsial, and parasitic pathogens [8], [9], [10], [11], [12]. These pathogens include *Babesia* spp., *Thelaria* spp., *Anaplasma* spp., *Borrelia* spp., *Ehrlichia* spp. However, in humans, the most common diseases transmitted by ticks are Lyme disease and ehrlichiosis, babesiosis, Colorado fever, Rocky Mountain fever, tularemia, Q fever, tick paralysis, and tick encephalitis [13], [14], [15] , [16].

Worldwide, hard tick infestations cost around $7 billion per year [17]. Whereas in the context of Nepal, economic loss due to ticks and tick-borne diseases in livestock was reported to be 18.71% of the total cost [18]. In the case of dogs, the large proportion of community dogs lacks basic health monitoring and treatment [19]. Those dogs harboring ticks and different TBPs may become a source for healthy individuals [19]. Previous records regarding TBPs and their potential health impacts highlight the probability of diverse tick species in different geographical locations and animal hosts.

There are limited studies regarding tick species prevalence, abundance, species diversity, and geographic distribution of ticks infesting animals in Nepal [18] Those limitations in systematic

research on prevalence, abundance, and distribution are one of the main reasons for the failure to attain effective preventive measures against ticks in Nepal [6]. The environmental conditions and inadequate preventive and control strategies have offered an ideal setting for tick species in Nepal. Thus, the research was conducted to identify ticks and observe the distribution pattern among different species of animals and geographical locations of Bagmati province, Nepal.

## 2. Methods

### 2.1 Study area

The study was carried out in Bagmati Province, which is in the central part of Nepal. Samples were collected from six different sampling areas (municipalities), which lie in three districts of the province: Kathmandu (mid hill), Chitwan (plain), and Rasuwa (high hill).

Chitwan is located at an altitude of 301–1000 m and subtropical to tropical climatic conditions with temperature 10 °C–38 °C, precipitation: 1,800 mm, humidity: 75–80%. Similarly, Kathmandu is located at an elevation of 1,262 m–2,732 m above sea level, with a subtropical to temperate climatic zone with temperature: 1 °C–30 °C, precipitation: 1,400 mm, humidity: 65%. Rasuwa, the Himalayan region at an altitude of 300–5000 masl, ranging from subtropical to alpine with temperature: -5 °C – 20 °C, precipitation: 1,200 mm, humidity: 70–80% (Figure 1)

### 2.2 Study duration and Population

Ticks were collected directly from the bodies of five different domestic animal species, including buffalo, cattle, sheep, goats, and dogs. The study was carried out from September 2023 to January

2024. A total of 210 animals were selected, including 60 dogs, 40 buffalo, 30 cattle, 50 sheep, and 30 goats.

### 2.3 Sample collection

In the study, only tick-infested animals were included. The ticks were carefully removed using forceps and gloved hands. During the detachment of the tick from the animal's body, the basis capitulum of the tick was held to ensure that the mouthpart remained intact. While collecting ticks from animals, the entire body was examined, particularly the ear, dewlap, perineal region, scrotum, udder, back, and inside the tuft of the tail. Approximately 2 to 5 ticks were collected from each animal [details in the results section, sample description (Table 1)]. The collected ticks from different animal species were kept in separate labeled vials containing 70% ethanol and were stored at 4 °C. Detailed information regarding the animals, such as age, sex, date of collection, severity of infestation, and locations, was also recorded.

### 2.4 Identification of ticks

The collected ticks were brought to the laboratory, where tick genera were identified using a stereo-zoom microscope. Further species-level identification was performed at magnification of 40X and 100X. For the identification, different key morphological features of ticks, such as scutum, basis capitula, palp, dentition, presence or absence of spur, adanal or accessory plates, festoon, were observed based on the published guidelines (20), (21), and (22).

### 2.5 Data analysis

Data entry and analysis of all variables and numerical data were conducted using MS Excel 2016. The data were analyzed quantitatively with a focus on species diversity, species richness, and

evenness. Additionally, the Simpson diversity index and Shannon-Wiener diversity index were calculated for each sampling district (region), animal categories [small ruminants (sheep and goats), large ruminants (cattle and buffalo), and pets (dogs)], as well as for the entire province using the formula below: -

**Shannon-Wiener diversity index (H) = - ∑ pi ln(pi)**

Where, pi proportion of each tick species

**Simpson's diversity index (S)= ∑ n(n-1)/N(N-1)**

Where n = number of individuals of that single species

N = No. of individuals of the total population

# 3. Results

## 3.1 Sample description

A total of 755 ticks were collected from 210 animals in Bagmati Province. Among these animals, there were 50 sheep, 30 goats, 30 cattle, 40 buffaloes, and 60 dogs. The number of male sheep was 6 (12%), compared to 44 female sheep (88%). For the goats, there were 13 males (43.33%) and 17 females (56.66%). All cattle were female, while 9 males (22.50%) and 26 females (77.50%) were found among the buffalo. The pet dogs were composed of 48 females (80%) and 12 males (20%). Most of the sheep and dogs in the study were aged 1–3 years, while the goats were less than 1 year old, and the cattle and buffalo were older than 3 years. During the sampling, most cattle, sheep, and goats had moderate tick infestations, whereas buffalo and dogs showed high levels of infestation (Table 1).

**Table 1.** Descriptive information on age, sex, and tick infestation severity on the host.

| Animal | Sex | | Age | | | Tick infestation severity | | |
|---|---|---|---|---|---|---|---|---|
| | Male | Female | ≤ 1yr | 1–3 yrs. | ≥3yrs. | Less | Moderate | High |
| **Sheep** | 6 | 44 | 11 | 26 | 13 | 8 | 22 | 20 |
| **Goat** | 13 | 17 | 12 | 11 | 7 | 5 | 18 | 7 |
| **Cattle** | - | 30 | 3 | 4 | 23 | 6 | 15 | 9 |
| **Buffalo** | 9 | 31 | 5 | 16 | 19 | 10 | 9 | 21 |
| **Dog** | 12 | 48 | 8 | 28 | 24 | 4 | 22 | 34 |
| **Total** | **40** | **170** | **39** | **85** | **86** | **33** | **86** | **91** |

There were 755 ticks in total, 17 (2.25%) of which were nymphs and 738 (97.75%) adults. Sexual dimorphism and identification of only adults were possible; however, only 5 ticks were classed as unidentified ticks. Male ticks accounted for 38.60% and female ticks for 61.3% of the total adult tick population, and the sex ratio of each species of tick genus was calculated (Table 2).

**Table 2.** Sex ratio of the tick population on domestic animals of the Bagmati province

| Tick species | Male tick count | Female tick count | Sex ratio (female: male) | Sex ratio of the genus |
|---|---|---|---|---|
| *Rhipicephalus microplus* | 86 | 137 | 1.59:1 | 1.14:1 |
| *Rhipicephalus decoloratus* | 15 | 6 | 0.40:1 | |
| *Rhipicephalus sanguineus* | 81 | 57 | 0.70:1 | |

| | | | | |
|---|---|---|---|---|
| *Rhipicephalus parva* | 1 | - | - | |
| *Rhipicephalus* spp. | - | 10 | - | |
| *Haemaphysalis sulcata* | - | 97 | - | |
| *Haemaphysalis longicornis* | 92 | 104 | 1.13:1 | 2.52:1 |
| *Haemaphysalis leachi* | - | 15 | - | |
| *Haemaphysalis* spp. | - | 16 | - | |
| *Dermacentor* spp. | 8 | 5 | 0.62:1 | 0.62:1 |
| *Hyalomma* spp. | - | 3 | - | - |
| **Total** | **283** | **450** | | |

### 3.2 Tick species identification and abundance

Ticks collected were identified to genus and species level, except nymphs and 5 unidentified ticks. During the study, 4 genera of ticks were identified (Fig. 2). Among them, two genera were identified to the species level, and the remaining to the genus level only. These include ticks like *Rhipicephalus* [*Rhipicephalus (Boophilus*) *microplus, Rhipicephalus (boophilus) decoloratus, Rhipicephalus parva, Rhipicephalus sanguineus, Rhipicephalus* spp.], *Haemaphysalis* (*Haemaphysalis longicornis, Haemaphysalis sulcata, Haemaphysalis leachi, Haemaphysalis* spp.*), Dermacentor spp.*, and *Hyalomma* spp. During identification, the main morphological features of ticks, like scutum, palp, basis capitula, dentition, festoon, coxae, and spur, were ~~detected~~ ( Figure 2)

In the current study, *Rhipicephalus* had the highest prevalence (53%) in domestic animals. *Rhipicephalus microplus* was found to be the most abundant species with 30.42%, followed by

*Haemaphysalis longicornis* with 26.74%, *Rhipicephalus sanguineus* with 18.83%, *Haemaphysalis sulcata* with 13.23%, *Rhipicephalus decoloratus* with 2.86%, and *Haemaphysalis spp*.2.18%, *Haemaphysalis leachi* 2.05%, *Dermacentor spp*. 1.77%, *Rhipicephalus spp*. 1.36%, *Hyalomma spp*. 0.41% and *Rhipicephalus parva* 0.14% (Table 3).

**Table 3.** The abundance of different identified tick species in domestic animals

| Tick Species | Abundance (%) |
|---|---|
| *Rhipicephalus microplus* | 30.42 |
| *Haemaphysalis longicornis* | 26.74 |
| *Rhipicephalus sanguineus* | 18.83 |
| *Haemaphysalis sulcata* | 13.23 |
| *Rhipicephalus decoloratus* | 2.86 |
| *Haemaphysalis leachi* | 2.05 |
| *Haemaphysalis* spp. | 2.18 |
| *Dermacentor* spp. | 1.77 |
| *Rhipicephalus* spp. | 1.36 |
| *Hyalomma* spp. | 0.41 |
| *Rhipicephalus parva* | 0.14 |
| **Total** | **100** |

### 3.3 Host-wise tick distribution

*Rhipicephalus microplus* had affected all four species of domestic ruminants, whereas *Rhipicephalus decoloratus* was found only in large ruminants. Moreover, the goats and sheep of

Bagmati province were mostly observed to be affected by *Haemaphysalis longicornis* (59.21%) and then by *Haemaphysalis sulcata* (31.5%). Large ruminants and dogs were affected less by these tick species. Mostly, *Rhipicephalus sanguineus* (76.6%) was observed in dog species and was not seen in domestic ruminants. Also, *Hyalomma* ticks were noted in sheep, and most of the *Dermacentor* spp. (76.92%) were found in goats. The host-wise distribution of ticks is presented in Table 4.

**Table 4**. Host-wise distribution of ticks

| **Tick species** | **Tick number** | | | | |
|---|---|---|---|---|---|
| | Cattle | Buffalo | Sheep | Goat | Dog |
| *Rhipicephalus microplus* | 78 | 142 | 2 | 1 | 0 |
| *Rhipicephalus decoloratus* | 7 | 14 | 0 | 0 | 0 |
| *Rhipicephalus sanguineus* | 0 | 0 | 0 | 0 | 138 |
| *Rhipicephalus* spp. | 0 | 0 | 0 | 0 | 10 |
| *Rhipicephalus parva* | 0 | 1 | 0 | 0 | 0 |
| *Haemaphysalis sulcata* | 1 | 0 | 75 | 21 | 0 |
| *Haemaphysalis longicornis* | 5 | 0 | 126 | 54 | 11 |
| *Haemaphysalis leachi* | 0 | 0 | 0 | 0 | 15 |
| *Haemaphysalis* spp. | 1 | 0 | 8 | 3 | 4 |
| *Dermacentor* spp. | 0 | 0 | 1 | 10 | 2 |
| *Hyalomma* spp. | 0 | 0 | 3 | 0 | 0 |

| | | | | | |
|---|---|---|---|---|---|
| **Total** | **92** | **157** | **215** | **89** | **180** |

### 3.4 Geographical distribution of ticks and diversity

More ticks were observed from Chitwan (90.72%) compared to Rasuwa (2.72%) and Kathmandu (6.54%). Total abundance and richness of tick species were observed to be higher in Chitwan (90.72%) or plain terai compared to hill and high hill. Similarly, *Rhipicephalus microplus* was found to be the most abundant tick in Chitwan (29.77%), followed by *Haemaphysalis longicornis* (28.57%), and the least abundant *Rhipicephalus parva* was (0.15%). *Rhipicephalus microplus* ticks from Kathmandu also showed the highest abundance (52.08%), whereas in Rasuwa, *Rhipicephalus sanguineus* (50%) and *Dermacentor* spp. (50%) were observed in equal proportion.

**Table 5.** Geographical distribution of ticks in different locations

| **Tick species** | **Abundances of tick species** | | | **Total abundance (%)** | **Total abundance of Genera** |
|---|---|---|---|---|---|
| | Chitwan | Kathmandu | Rasuwa | | |
| *Rhipicephalus microplus* | 198 | 25 | - | 223 (30.42) | |
| *Rhipicephalus decoloratus* | 21 | - | - | 21(2.86) | |
| *Rhipicephalus sanguineus* | 113 | 15 | 10 | 138 (18.83) | 393 (53%) |
| *Rhipicephalus parva* | 1 | - | - | 1 (0.14) | |
| *Rhipicephalus* spp. | 10 | - | | 10 (1.36) | |
| *Haemaphysalis sulcata* | 97 | - | | 97 (13.23) | |
| *Haemaphysalis longicornis* | 190 | 6 | | 196 (26.74) | 324 (44.2%) |
| *Haemaphysalis leachi* | 15 | - | | 15 (2.05) | |
| *Haemaphysalis* spp. | 14 | 2 | | 16 (2.18) | |
| *Dermacentor* spp. | 3 | - | 10 | 13 (1.77) | 13 (1.7%) |
| *Hyalomma* spp. | 3 | - | - | 3 (0.40) | 3 (0.4%) |

| | | | | | |
|---|---|---|---|---|---|
| **Total** | 665 | 48 | 20 | 733 | |

### 3.5 Species richness and diversity

Species richness was found to be higher in the plain of Bagmati province (13 species), followed by mid-hill (4 spp.) and high hill/mountain (2 spp.) (Table 6). In the case of Shannon Wiener diversity index, the values calculated were 1.7, 1.1, and 0.693, respectively, from plain, mid-hill, and high hill. Among these regions, the terai was found to have high diversity, and the high hills had low diversity. Moreover, the Simpson indices for various species of ticks in three geographical regions were 0.22 for plain, 0.37 for mid-hill, and 0.47 for high hill. This also showed the highest diversity in plains, as the lower the value of Simpson's index, the higher the diversity.

Among large ruminants, small ruminants, and pets, a total of 6 species of ticks were identified, i.e., all animal species infested by the same number of ticks. The Shannon Wiener diversity index was higher for small ruminants (1.01), followed by dogs (0.877), and large ruminants (0.463) (Table 6). This showed more diversity of ticks among small ruminants and less diversity in large ruminants. In addition, Simpson's diversity index values for large ruminants (0.45), small ruminants (0.79), and dogs (0.6) indicate higher diversity in ticks among small ruminants. The overall value of the Shannon-Wiener diversity index of ticks among domestic animals of Bagmati province was 1.72, and Simpson's diversity index was 0.22.

**Table 6.** Calculation of tick species diversity among different areas and hosts

| Parameters | Bagmati Province | Large Ruminants | Small Ruminants | Dogs | Plain | Mid-hill | High hill |
|---|---|---|---|---|---|---|---|
| **Species richness** | 11 | 6 | 6 | 6 | 11 | 4 | 2 |

| | | | | | | | |
|---|---|---|---|---|---|---|---|
| **Shannon Wiener's diversity index** | 1.72 | 0.46 | 1.01 | 0.87 | 1.70 | 1.10 | 0.69 |
| **Simpson's Diversity Index** | 0.22 | 0.79 | 0.45 | 0.60 | 0.22 | 0.37 | 0.47 |

## 4. Discussion

In this study, four genera of ticks (*Rhipicephalus*, *Haemaphysalis*, *Dermacentor*, and *Hyalomma*) were identified among the domestic animals and pets of different geographical regions of central Nepal. Among the four genera, *Rhipicephalus* had four species (*R. microplus*, *R. decoloratus*, *R. sanguinus*, *R. Sanguineus*), and *Haemaphysalis* had three species (*H. sulcata*, *H. longicornis*, *H. leachi*); however, the two genera, *Dermacentor* and *Hyalomma*, were not fully identified up to the species level based on the morphological characteristics. According to existing information, this is the first study on the diversity and spatial distribution of tick species infesting domestic animals.

The prevalence and host associations of various tick species in this study showed distinct patterns across the different livestock and companion animals. The current study revealed the prevalence of three genera of ticks in goats, including *Rhipicephalus*, *Haemaphysalis*, and *Dermacentor*, similar to those of [18]. The presence of more Ixodids in goats could be because of the sampling duration, as this study was conducted mainly in the winter period (unfavorable conditions) [23]. These ticks are responsible for transmitting *Anaplasma*, *Babesia*, and *Theileria* in goats [24]. Similarly, *H. longicornis* was the most abundant and predominant species of tick infesting sheep, which is supported by [25]; however, it contrasts with [26], which showed *R. sanguineus* as the dominant tick species. These may be due to geographical differences, grazing patterns, and

sampling sites in the small ruminants like the ear and head [27]. *Haemaphysalis longicornis* is mainly responsible for transmitting *Rickettsia*, *Anaplasma*, and *Borrelia* in sheep and can potentially infect humans, also causing Japanese spotted fever (*Rickettsia japonica*), Anaplasmosis (*Anaplasma* spp.), and Lyme disease (*Borrelia* spp.) [28]. Therefore, the study highlights the prevalence and distribution of various tick species in small ruminants, emphasizing their role in pathogen transmission and the potential health risks among animals and humans.

Tick infestation patterns in cattle and buffaloes revealed the predominance of a single species, with important implications for disease transmission. *Rhipicephalus microplus* was the most abundant among the cattle, which is supported by  [29] The tick species is responsible for transmitting *Rickettsia* spp., *Anaplasma* spp., and *Ehrlichia* spp. in cattle [30]. Similarly, the predominant tick infesting buffaloes was *Rhipicephalus microplus*, which is in agreement with the findings of [31] Thus, *R. microplus* emerged as the dominant tick species in both cattle and buffaloes, highlighting its significant role in the transmission of important pathogens in Central Nepal.

In the case of a dog, a high prevalence of *Rhipicephalus sanguineus* was observed. These ticks are the potential cause of transmitting *Anaplasma* spp., *Ehrlichia* spp., and *Babesia* spp. in dogs [32]. All these findings were supported by [6] and [29]. Therefore, in dogs, *R. sanguineus* was highly prevalent and recognized as a major vector of several important pathogens. In addition to species prevalence and pathogen transmission, the sex ratio within tick populations further influences their reproductive success and associated health risks to domestic animals.

The analysis of tick populations on domestic animals revealed notable variations in sex ratios, which have important implications for reproduction, pathogen transmission, and host health. The sex ratio of the tick (female: male) population on domestic animals showed that *Rhipicephalus* spp. and *Haemaphysalis* spp. have a greater proportion of female ticks, but lower in the case of

*Dermacento*r. These findings are similar to [33] however, somewhat in contrast with those [13], which showed that the ratio of females to males was lower. Moreover, a sex ratio greater than one may lead to a higher reproductive potential among the genera of *Rhipicephalus* spp. and *Haemaphysalis* spp., in turn, increasing the risk of TBPs [34]. Conversely, in the case of *Dermacentor* spp., males were in high proportion, reflecting the lower risks of TBP transmission and blood loss [35], [36]. Therefore, a sex ratio favoring females in a tick population implies a greater potential than male ticks for reproduction, pathogen transmission, and economic impact on the host animals.s

Species richness was notably highest in the Terai, declining sharply in the mid-hills and high hills/mountains. Most of the ticks used in the study were from the Terai region, and a smaller sample was collected from the hills and Himalayas, which may be due to the tropical and sub-tropical climate in the Terai and geographical differences, as well as the low prevalence of ticks in winter or cold areas. This is in conjunction with [6], which also reported the highest number of ticks in Chitwan compared to Dang and Lamjung. This trend is consistent with ecological expectations, where lower elevations generally offer more favorable environmental conditions (like higher temperature, humidity, and vegetation cover) to enhance the greater biodiversity of ticks [37]. Along with the species' richness, the tick diversity was also higher in the case of the Terai than in the hill and the high hill/ mountain. This might be due to the greater number of domestic hosts involved in the study in Terai under favorable environmental conditions. This indicates that in the Terai regions, the probability of getting infected with TBPs is higher in animals. Higher species richness in plain areas increases the risk of transmitting a wider range of TBPs to animals and humans, complicating disease control efforts. Therefore, areas with high tick

diversity require more comprehensive surveillance and management strategies to mitigate health and economic impacts.

Not all the ticks collected were fully identified because of the presence of different stages of ticks, like engorged females, nymphs, and larvae, where identification based on key morphological features was impossible. The reason might be due to variation in ornamentation among different geographical regions, and under the limitations of resources for confirmation. The presence of engorged females could be another reason for the *Hyalomma* species. Similarly, some of the engorged females of *Rhipicephalus* and *Haemaphysalis* were only identified up to the genus level due to a lack of visible morphological features. Out of the total ticks, the study showed five of them as unidentified ticks. This was best explained by the loss of mouth parts during the collection of ticks and the absence of major identifying features. Thus, morphology-based identification of ticks depends on proper sample collection, preservation, and careful observation.

In the future, researchers should focus on molecular identification of tick species and pathogens associated with them to better understand disease risks. Moreover, long-term ecological studies and integrated tick management strategies are essential to predict changes in tick distribution and mitigate the rising threat of TBPs under environmental and climate change scenarios.

**Constraints of the study**

Mainly, sampling was performed during the winter. The season so far for this short period may miss some species. Based on morphology, there could be some technical errors. Health impact and TBPs of animal and public health were not included due to time and resource constraints.

## 5. Conclusions

The study showed four genera of ticks, prevalent in different domestic animals and in three districts of Bagmati province, ranging from plain to high hill, which include *Rhipicephalus, Haemaphysalis, Dermacentor*, and *Hyalomma*. The tick distribution and diversity are higher in Chitwan as compared to other areas, which may increase the risks of transmission of various TBPs. Moreover, some of the ticks have zoonotic importance that needs to be further explored with priority. Ultimately, safeguard the health of animals and the public from TBPs.

**Acknowledgements-**

The authors are grateful to the Department of Microbiology and Parasitology, Faculty of Animal Science, Veterinary Science and Fisheries, Agriculture and Forestry University for Laboratory facilitation to conduct research. Additionally, the authors would like to thank the farmers and pet owners for their cooperation during the sampling process.

**Data Availability Statement**

All relevant data are contained within the manuscript.

**Financial Disclosure Statement**

For this research, we had no specific grant as funding agencies

**Competing interests-** All authors have approved and there are no competing interests**.**

**Author's contributions- Kamana Thapa**: Writing- original draft preparation, Visualization, Project administration, Methodology, Investigation, Formal analysis, data curation, Conceptualization. **Somnath Aryal**: Methodology, Writing, review, and editing. **Surendra**

**Prasad Kanu**: Supervision, Manuscript review, and editing. **Chet Raj Pathak**: Supervision, Validation, Methodology, Manuscript preparation, Formal analysis, Writing – review and editing. All authors commented on the previous versions of this manuscript. All authors have read and approved the final manuscript.

**Figure legends**

**Figure. 1**. Sampling points for ticks from different farm animals and pets.

**Figure. 2.** Different species of ticks were detected. A. *Rhipicehalus sanguineus* male, (i) dorsal, (ii)ventral; B. *R. sanguineus* female, (i) dorsal, (ii) ventral, (a) mouth parts; C. *R. microplus* male, (i) dorsal, (ii) ventral; D. *R. microplus* female, (i) dorsal, (ii) ventral, (b) mouthpart; E. *R.decoloratus* male, (i) dorsal, (ii) ventral; F. *R. parva* male, (i) dorsal (ii) ventral; G. *Haemaphysalis sulcate* female, (i) dorsal; H. *H. longicornis* male, (i) dorsal, (ii) ventral; I. *H.longicornis* female, (i) dorsal, (ii) ventral, (c) mouthpart; J. *H. leachi* male, (i) dorsal, (ii) ventral; K. *Dermacentor sp*. male, (i) dorsal, (ii) ventral; L. *Hyalomma sp.* female, (i)dorsal.

## References


1. Farahi A, Ebrahimzade E, Nabian S, Hanafi-bojd AA, Akbarzadeh K, Bahonar A. Temporal and Spatial Distribution and Species Diversity of Hard Ticks (Acari: Ixodidae) in the Eastern Region of Caspian Sea. Acta Trop. 2016;

2. Ramzan M, Naeem-ullah U, Saba S, Iqbal N. Prevalence and identification of tick species ( Ixodidae ) on domestic animals in district Multan , Punjab Pakistan. Int J Acarol. 2020;00(00):1–5.

3. Rehman A, Nijhof AM, Sauter-louis C, Schauer B, Staubach C, Conraths FJ. Distribution of ticks infesting ruminants and risk factors associated with high tick prevalence in livestock farms in the semi- arid and arid agro-ecological zones of Pakistan. 2017;1–15.

4. Guglielmone AA, Robbins RG, Apanaskevich DA, Petney TN, Estrada-Peňa A, Horak IG, et al. The Argasidae, Ixodidae and Nuttalliellidae (Acari: Ixodida) of the world: a list of valid species names. Zootaxa [Internet]. 2020 Jul 6 [cited 2025 May 7];2528(2528):1–28. Available from: https://ri.conicet.gov.ar/handle/11336/97869

5. Yadav N, Sci RUASciMed, 2024 undefined. Status of Economic Losses and Health Impact of the Tick Bites on Farm, Field and Dairy Animals. researchgate.net [Internet]. [cited 2025 Apr 4]; Available from: https://www.researchgate.net/profile/Ravi-Upadhyay/publication/386540639_Status_of_Economic_Losses_and_Health_Impact_of_the_Tick_Bites_on_Farm_Field_and_Dairy_Animals/links/675518a1ad10b614ef36f4a7/Status-of-Economic-Losses-and-Health-Impact-of-the-Tick-Bites-on-Farm-Field-and-Dairy-Animals.pdf

6. Dhital B, Shrestha S, … KKJ of A and, 2018 undefined. Distribution of the cattle ticks from mid hills to plains of Nepal. nepjol.info [Internet]. 2018 [cited 2025 May 7];1(1):197–205. Available from: https://nepjol.info/index.php/janr/article/view/22235

7. Badji A, Ndiaye M, Gaye A, Dieng I, Ndiaye EH, Dolgova AS, et al. Detection of Crimean – Congo Haemorrhagic Fever Virus from Livestock Ticks in Northern , Central and Southern Senegal in 2021. 2023;

8. Barandika JF, Hurtado A, Garcı C, Gil H, Escudero R, Barral M, et al. Tick-Borne Zoonotic Bacteria in Wild and Domestic Small Mammals in Northern Spain □. 2007;73(19):6166–71.

9. Labuda M, Nuttall PA. Tick-borne viruses. Parasitology [Internet]. 2004 [cited 2025 Apr 23];129(S1):S221–45. Available from: https://www.cambridge.org/core/journals/parasitology/article/tickborne-viruses/E8217B4D74B049183A25DCC4CCA03E3D

10. Baneth G. Tick-borne infections of animals and humans: a common ground. Int J Parasitol [Internet]. 2014 Aug 1 [cited 2025 Apr 23];44(9):591–6. Available from: https://www.sciencedirect.com/science/article/pii/S0020751914000964

11. Witter R, Martins TF, Campos AK, Melo ALT, Corrêa SHR, Morgado TO, et al. Rickettsial infection in ticks (Acari: Ixodidae) of wild animals in midwestern Brazil. Ticks Tick Borne Dis [Internet]. 2016 Apr 1 [cited 2025 Apr 23];7(3):415–23. Available from: https://www.sciencedirect.com/science/article/abs/pii/S1877959X15300601

12. Sevinc F, Sciences XXEJ of V, 2015 undefined. Major tick-borne parasitic diseases of animals: A frame of references in Turkey. dergipark.org.tr [Internet]. 2015 [cited 2025 Apr 23];31:132–42. Available from: https://dergipark.org.tr/en/download/article-file/228535

13. Ganjali M, Dabirzadeh M, Sargolzaie M. Species Diversity and Distribution of Ticks ( Acari : Ixodidae ) in Zabol County , Eastern Iran. 2014;8(December):219–23.

14. Gharekhani J, … AGSJ of C, 2015 undefined. Determination of hard tick species (Acarina: Ixodidae) on sheep and cattle in Hamedan Province, Iran. researchgate.net [Internet]. [cited 2025 Apr 23]; Available from: https://www.researchgate.net/profile/Mohammad-Reza-Youssefi/publication/280293884_Determination_of_hard_tick_species_Acarina_Ixodidae_on_sheep_and_cattle_in_Hamedan_Province_Iran/links/55afe06508aeb9239917170b/Determination-of-hard-tick-species-Acarina-Ixodidae-on-sheep-and-cattle-in-Hamedan-Province-Iran.pdf

15. Jafari B, Moghaddam HS, … PV, 2020 undefined. New Record and Description of Dermacentor marginatus (Acari: Ixodidae) from Ovis orientalis arkal (Mammalia: Bovidae) in Northeastern Iran. ecopersia.modares.ac.ir [Internet]. 2019 [cited 2025 Apr 23]; Available from: http://ecopersia.modares.ac.ir/article-24-21641-en.html

16. Körner S, Makert GR, Ulbert S, Pfeffer M, Mertens-Scholz K. The Prevalence of Coxiella burnetii in Hard Ticks in Europe and Their Role in Q Fever Transmission Revisited—A Systematic Review. frontiersin.org [Internet]. 2021 Apr 26 [cited 2025 Apr 23];8:655715. Available from: https://www.frontiersin.org/articles/10.3389/fvets.2021.655715/full

17. Hassan JUL, Rizwan HM, Hassan UL, Rizwan HM. Journal of the Hellenic Veterinary Medical Society. 2018;68(1):51–8.

18. Kunwar A, Shakya SR, Ghimire TR. Original paper Nepal. 2022;68(2):287–96.

19. Regañón DD, Agulla B, Piya B, Ruiz NF, Villaescusa A, Sancho MG, et al. Stray dogs in Nepal have high prevalence of vector - borne pathogens : a molecular survey. Parasit Vectors. 2020;1–8.

20. Walker AR, Bouattour A, Horak I. Ticks of Domestic Animals in Africa : a Guide to Identification of Species. 2014.

21. Barker S, Walker AR. Ticks of Australia. The species that infest domestic animals and humans. 2014.

22. Egizi AM, Robbins RG, Beati L, Nava S, Evans CR, Occi JL, et al. A pictorial key to differentiate the recently detected exotic Haemaphysalis longicornis Neumann , 1901 ( Acari , Ixodidae ) from native congeners in North America. 2019;128:117–28.

23. Madder M, Horak I, … HS: F of veterinary SU of, 2014 undefined. Tick identification. afrivip.org [Internet]. [cited 2025 May 7]; Available from: https://afrivip.org/sites/default/files/identification_complete_1.pdf

24. Ghafar A, Abbas T, Rehman A, Sandhu ZUD, Cabezas-Cruz A, Jabbar A. Systematic review of ticks and tick-borne pathogens of small ruminants in Pakistan. mdpi.comA Ghafar, T Abbas, A Rehman, ZD Sandhu, A Cabezas-Cruz, A JabbarPathogens,

2020•mdpi.com [Internet]. 2020 Nov 1 [cited 2025 Apr 5];9(11):1–32. Available from: https://www.mdpi.com/2076-0817/9/11/937

25. Teng Z, Shi Y, Zhao N, Zhang X, Jin X, He J, et al. Molecular Detection of Tick-Borne Bacterial and Protozoan Pathogens in Haemaphysalis longicornis (Acari: Ixodidae) Ticks from Free-Ranging Domestic Sheep in Hebei Province, China. Pathogens [Internet]. 2023 Jun 1 [cited 2025 May 7];12(6):763. Available from: https://www.mdpi.com/2076-0817/12/6/763/htm

26. Shemshad K, Rafinejad J, Kamali K, Piazak N. Species diversity and geographic distribution of hard ticks ( Acari : Ixodoidea : Ixodidae ) infesting domestic ruminants , in Qazvin Province , Iran. 2012;373–80.

27. Zheng H, Yu Z, Zhou L, Yang X, Liu J. Seasonal abundance and activity of the hard tick Haemaphysalis longicornis (Acari: Ixodidae) in North China. Springer [Internet]. 2012 Feb [cited 2025 May 7];56(2):133–41. Available from: https://link.springer.com/article/10.1007/s10493-011-9505-x

28. Teng Z, Shi Y, Zhao N, Zhang X, Jin X, He J, et al. Molecular Detection of Tick-Borne Bacterial and Protozoan Pathogens in Haemaphysalis longicornis (Acari: Ixodidae) Ticks from Free-Ranging Domestic Sheep in Hebei Province, China. Pathogens [Internet]. 2023 Jun 1 [cited 2025 Apr 4];12(6):763. Available from: https://www.mdpi.com/2076-0817/12/6/763/htm

29. Anish RK, Venu R, Rayulu VC, Jacob SS, Srilatha CH. Prevalence and diversity of ixodid tick fauna in domestic animals of Andhra Prevalence and diversity of ixodid tick fauna in domestic animals of Andhra Pradesh state , India. 2020;(October).

30. Xu J, Gu XL, Jiang ZZ, Cao XQ, Wang R, Peng QM, et al. Pathogenic Rickettsia, Anaplasma, and Ehrlichia in Rhipicephalus microplus ticks collected from cattle and laboratory hatched tick larvae. 2023 [cited 2025 Apr 5]; Available from: https://doi.org/10.1371/journal.pntd.0011546.g001

31. Naseem A, Khan MA, Ali SZ. Prevalence and risk factors associated with tick infestation of buffaloes in the eastern part of Uttar Pradesh, India. Int J Acarol. 2020;344–50.

32. Pandey GS, Pathak CR, Thapa S, Sadaula A, Manandhar P, Abdelbaset AE, et al. Exploring tick-borne pathogens in community dogs in Nepal. Parasitol Int. 2025 Jun 1;106:103003.

33. Dabaja MF, Tempesta M, Bayan A, Vesco G, Greco G, Torina A, et al. Diversity and distribution of ticks from domestic ruminants in Lebanon. 2014;147–55.

34. Oosten A Van, Duron O, diseases DHT and tick borne, 2018 undefined. Sex ratios of the tick Ixodes arboricola are strongly female-biased, but there are no indications of sex-distorting bacteria. Elsevier [Internet]. [cited 2025 Apr 22]; Available from: https://www.sciencedirect.com/science/article/pii/S1877959X17304041

35. bioRxiv EA, 2025 undefined. The Prevalence and Identification of Livestock Tick by Sex Ratio and Host in Tehran Province. biorxiv.orgE AbbasibioRxiv, 2025•biorxiv.org [Internet]. [cited 2025 Apr 23]; Available from: https://www.biorxiv.org/content/10.1101/2025.03.13.643006.abstract

36. Andrade-Ponce G, Giles B, … BNIJ for, 2025 undefined. Different drivers, same tick: Effect of host traits, habitat, and climate on the infestation of three rodent species by larval Dermacentor ticks. ElsevierGP Andrade-Ponce, BG Giles, BC Newman, AM López-Pérez, CB EversoleInternational Journal for Parasitology: Parasites and Wildlife, 2025•Elsevier [Internet]. [cited 2025 Apr 22]; Available from: https://www.sciencedirect.com/science/article/pii/S2213224425000197

37. Cunze S, Glock G, Kochmann J, Klimpel S. Ticks on the move—climate change-induced range shifts of three tick species in Europe: current and future habitat suitability for Ixodes ricinus in comparison with. SpringerS Cunze, G Glock, J Kochmann, S KlimpelParasitology research, 2022•Springer [Internet]. 2022 Aug 1 [cited 2025 Apr 23];121(8):2241–52. Available from: https://link.springer.com/article/10.1007/s00436-022-07556-x

Figure 1

Click here to access/download;Figure;Figure_1 tif.tif

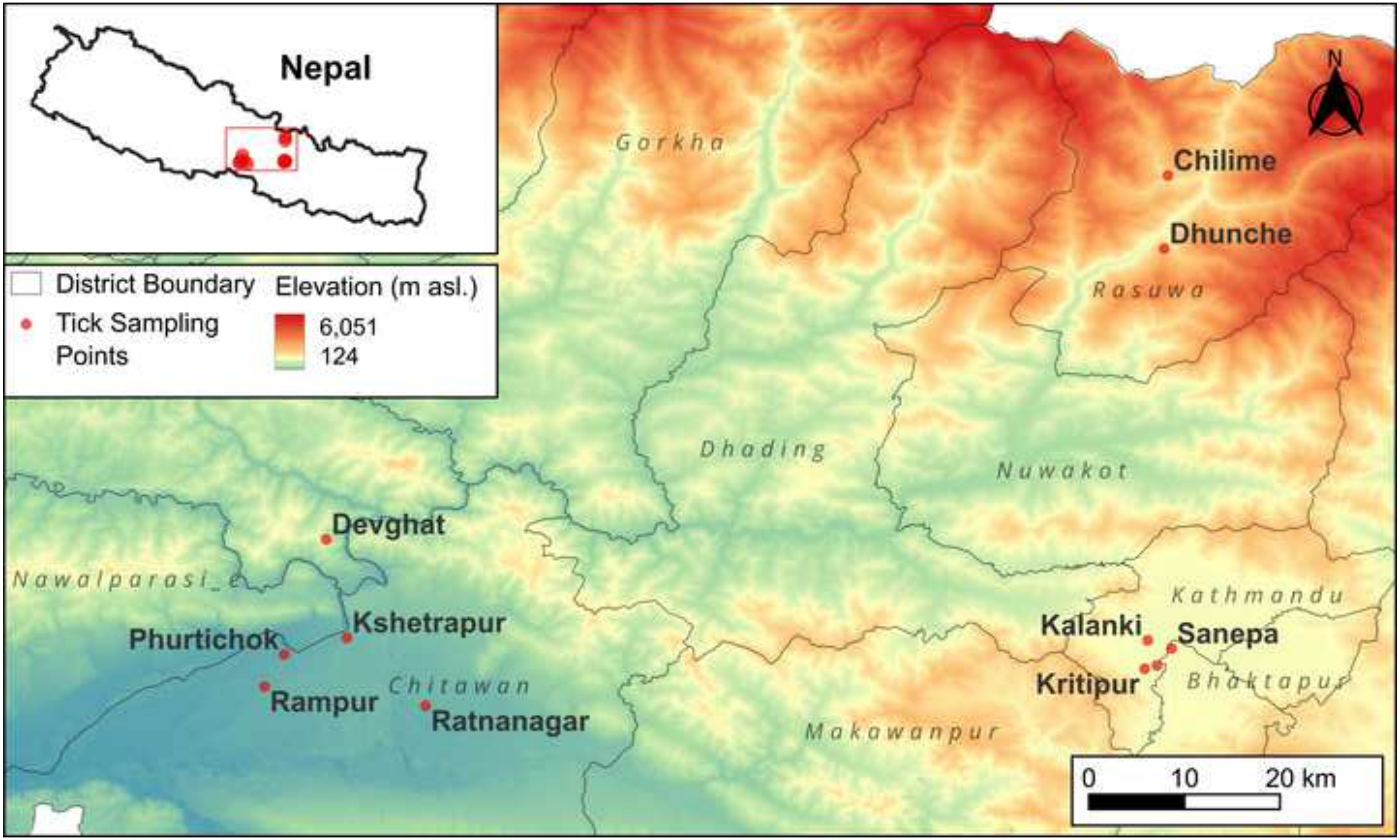

Figure 2

Click here to access/download;Figure;Figure_2 tif.tif

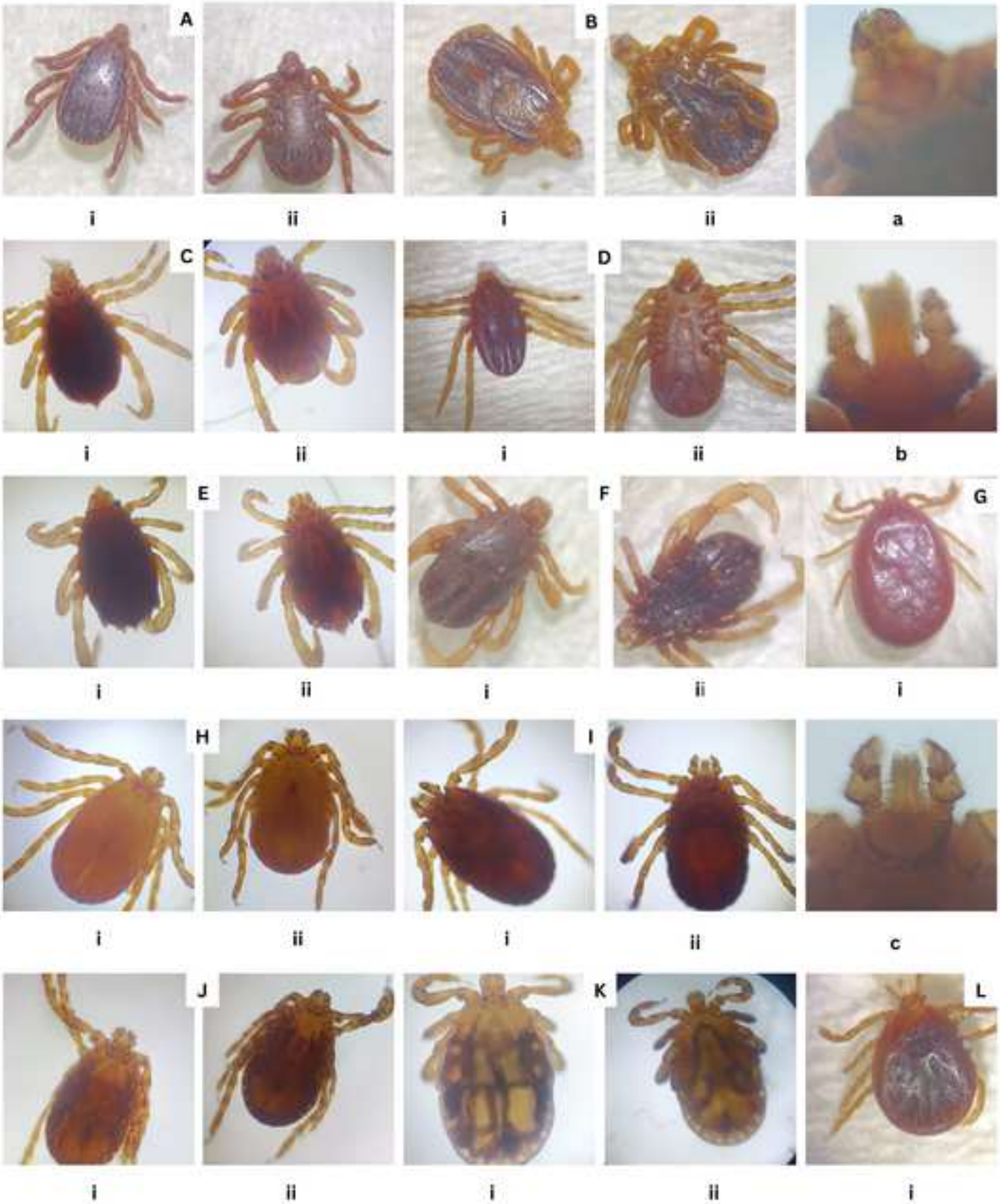

Supplement: Attachment — Submitted filename: PONE-D-25-27830_reviewer.pdf [file pone.0351151.s002.pdf]
