# Supplementary material for: Species diversity and geographical distribution of ticks infesting domestic animals in Bagmati Province, Nepal
Source: PLoS One. 2026 Jun 8;21(6):e0351151. doi: 10.1371/journal.pone.0351151 (PMC13245791; doi:10.1371/journal.pone.0351151)
Supplement: Attachment — Submitted filename: Ethical concerns.docx [file pone.0351151.s003.docx]

**Dear Editor: Thank you very much for providing me an opportunity to review the MS. Here are few comments/feedback to the authors.**

**Thank you.**

**Sincerely,**

**Reviewer**

**…………………………………………………..**

**Ethical concerns: Are the studies approved by Ethical Committee of the country? If yes, please provide the details about ethics.**

**I think the country has veterinary ethical body regarding the study ethical clearance and authors should state if so.**

Abstract: Total animals = 210

**The sample size for whole of the study areas at provincial level is very low.**

66 Ectoparasites constitute a significant problem in most developing and underdeveloped countries

**Which sentence/science/citation support this sentence?**

114 buffalo, cattle, sheep, goats, and dogs. The study was carried out from September 2023 to January

**To collect so few samples, you are almost using two seasons; September last of rainy season and January, the winter season. How can you relate the results over the different periods?**

122 udder, back, and inside the tuft of the tail. Approximately 2 to 5 ticks were collected from each

**You said you collected about 2 to 5 ticks only, it means, you are leaving other ticks.**

140 Shannon-Wiener diversity index (H) = - Σ pi ln(pi)

**If you are collecting 2 to 5 ticks only, then, how can H represents accurate values?**

142 Simpson’s diversity index (S)= Σ n(n-1)/N(N-1)

**Also the S values, how can it be assessed in each host?**

148 A total of 755 ticks were collected from 210 animals in Bagmati Province.

**It means, the numbers of ticks from 210 animals is very very low in this Province. I think the data will not be so much significant for the global readers.**

**However, the low data is highly significant in the context of taxonomic data.**

173 (*Haemaphysalis longicornis, Haemaphysalis sulcata, Haemaphysalis leachi, Haemaphysalis*

174 spp*.), Dermacentor spp.,* and *Hyalomma* spp. During identification, the main morphological

**What does it mean by *Hyalomma* spp. and *Haemaphysalis* spp.?**

181 *Haemaphysalis leachi* 2.05%, *Dermacentor spp*. 1.77%, *Rhipicephalus spp*. 1.36

**Similar for *Dermacentor* spp. and *Rhipicephalus* spp.?**

**It is difficult to explain rather than describe different species diversities, abundance based on a few samples of ticks collected.**

**Therefore, due to low sample collections, although the species identification needs further revision of the MS, especially regarding shortening of the MS. Also authors may make it significant in the context of pathology and explain why different organs/tissues are preferably infested by ticks. However, due to collection of equal/unequal numbers of ticks from few hosts, may make the wrong conclusions.**

**Thank you.**
