# Supplementary material for: Species diversity and geographical distribution of ticks infesting domestic animals in Bagmati Province, Nepal
Source: PLoS One. 2026 Jun 8;21(6):e0351151. doi: 10.1371/journal.pone.0351151 (PMC13245791; doi:10.1371/journal.pone.0351151)
Supplement: Attachment — Submitted filename: plos Comment 1.docx [file pone.0351151.s004.docx]

Comment 1

Line no 210 Bagmati province (13 species), But in table it is 11 which one is correct

Comment 2

Actual species is 7. Other are spp. How the index calculated it is misleading.

The spp is not identified and cannot be included in analysis as it is considering as separate species.

Comment 3.

Why the study is in winter, and author himself stated unfavourable.

Comment: 4

What is sampling method. How many domestic animals were available, how many screened, and how many tick infested.

There should be table for Tick infestation rate.

Comment 5:

There is no statistical analysis. For scientific publication there is need of statistical analysis for tick infestation rate, tick abundance, and tick intensity. This will give actual burden of the ticks in the study area.

Comment 6

The Shannon Wiener diversity index

was higher for small ruminants (1.01), followed by dogs (0.877), and large ruminants (0.463)

(Table 6). This showed more diversity of ticks among small ruminants and less diversity in large ruminants.

Wrong interpretation of index. Check reference.

Comment.7

Of the identified species, how many are reported as vectors (zoonosis) transmit to human and domestic animals in Nepal. Give emphasis on this aspects,

Comment 8

How Rhipicephalus decolaratus has been identified. It is very rare and difficult for identification .

Author can avoid sentence such as difficult to identify female and nymph. In the discussion that part of paragraph has to removed and it is common issue and it is not new research finding. This is species diversity paper and experienced taxonomist able to identify all stages of ticks.

Line 292 to 301 statement is not true. Any statement should be given wit published refererence. It will mislead to the reader.

Comment: 9

**Constraints of the study**

Should be modified that the author itself not confidence.

Comment 10

Line no 207. Total abundance of genera column is misleading. It has to be removed,

Overall comment

It is basic work. Many statements are assumption rather than evidene.
