# Supplementary material for: Species diversity and geographical distribution of ticks infesting domestic animals in Bagmati Province, Nepal
Source: PLoS One. 2026 Jun 8;21(6):e0351151. doi: 10.1371/journal.pone.0351151 (PMC13245791; doi:10.1371/journal.pone.0351151)
Supplement: Attachment — Submitted filename: Response to Reviewers_Final.docx [file pone.0351151.s007.docx]

**Point-by-point response to the editor and reviewers**

**PONE-D-25-27830**

Species diversity and geographical distribution of ticks infesting domestic animals in Bagmati province, Nepal

**Editors’ Comments**

Thank you for submitting your manuscript to PLOS ONE. After careful consideration, we feel that it has merit but does not fully meet PLOS ONE’s publication criteria as it currently stands. Therefore, we invite you to submit a revised version of the manuscript that addresses the points raised during the review process.

Please submit your revised manuscript by Sep 25, 2025, 11:59PM. If you will need more time than this to complete your revisions, please reply to this message or contact the journal office at [plosone@plos.org](mailto:plosone@plos.org). When you're ready to submit your revision, log on to <https://www.editorialmanager.com/pone/> and select the 'Submissions Needing Revision' folder to locate your manuscript file.

Please include the following items when submitting your revised manuscript:

- A rebuttal letter that responds to each point raised by the academic editor and reviewer(s). You should upload this letter as a separate file labeled 'Response to Reviewers'.
- A marked-up copy of your manuscript that highlights changes made to the original version. You should upload this as a separate file labeled 'Revised Manuscript with Track Changes'.
- An unmarked version of your revised paper without tracked changes. You should upload this as a separate file labeled 'Manuscript'.

If you would like to make changes to your financial disclosure, please include your updated statement in your cover letter. Guidelines for resubmitting your figure files are available below the reviewer comments at the end of this letter.

- Thank you for providing an opportunity to revise and improve our manuscript based on the valuable comments and suggestions from the editor and reviewers.
- We have written this response letter (point-by-point response) to respond specifically to the editor´s and reviewers´ comments, as outlined below.
- Bullet points with texts in red font in this response letter represent our specific response to each comment or suggestion.
- Specific line numbers in the parentheses are provided in this letter to track our changes easily in the revised manuscript file (separate file)
- Any changes in the revised manuscript are marked with texts in red.
- Along with this letter, we have uploaded a revised version of the manuscript, as well as a clean version of the revised manuscript.

Read more information on sharing protocols at [https://plos.org/protocols?utm_medium=editorial-email&utm_source=authorletters&utm_campaign=protocols](https://track.editorialmanager.com/CL0/https:%2F%2Fplos.org%2Fprotocols%3Futm_medium=editorial-email%26utm_source=authorletters%26utm_campaign=protocols/1/010f01989a3f859b-773c37a0-945c-4a5e-8e1c-f83cb1be9d61-000000/dyOJYbRmtMwObmxTq4v-cEYUBcMaSRBR0GyLPFE5nas=223).

- Thank you. We have followed all the guidelines regarding the authorship policies of the journal.
  We look forward to receiving your revised manuscript.

**Journal Requirements:**

When submitting your revision, we need you to address these additional requirements.

1. Please ensure that your manuscript meets PLOS ONE's style requirements, including those for file naming. The PLOS ONE style templates can be found at

<https://journals.plos.org/plosone/s/file?id=wjVg/PLOSOne_formatting_sample_main_body.pdf> and

[https://journals.plos.org/plosone/s/file?id=ba62/PLOSOne_formatting_sample_title_authors_affiliations.pdf](https://track.editorialmanager.com/CL0/https:%2F%2Fjournals.plos.org%2Fplosone%2Fs%2Ffile%3Fid=ba62%2FPLOSOne_formatting_sample_title_authors_affiliations.pdf/1/010f01989a3f859b-773c37a0-945c-4a5e-8e1c-f83cb1be9d61-000000/mOo4kFt1OTXZpxYe-gCQRFJM9QZL6azyFg-KU1IBfDM=223)

- We have now revised the manuscript format accordingly [Title page (Line: 1-18 and Headings (Line: 20, 59, 97,157,250, 349,356,365, 373)].

2. We note that Figure 1 in your submission contains map/satellite images which may be copyrighted. All PLOS content is published under the Creative Commons Attribution License (CC BY 4.0), which means that the manuscript, images, and Supporting Information files will be freely available online, and any third party is permitted to access, download, copy, distribute, and use these materials in any way, even commercially, with proper attribution. For these reasons, we cannot publish previously copyrighted maps or satellite images created using proprietary data, such as Google software (Google Maps, Street View, and Earth). For more information, see our copyright guidelines: <http://journals.plos.org/plosone/s/licenses-and-copyright>.

 We require you to either (1) present written permission from the copyright holder to publish these figures specifically under the CC BY 4.0 license, or (2) remove the figures from your submission:

 1. You may seek permission from the original copyright holder of Figure 1 to publish the content specifically under the CC BY 4.0 license.

 We recommend that you contact the original copyright holder with the Content Permission Form (<http://journals.plos.org/plosone/s/file?id=7c09/content-permission-form.pdf>) and the following text:

“I request permission for the open-access journal PLOS ONE to publish XXX under the Creative Commons Attribution License (CCAL) CC BY 4.0 (<http://creativecommons.org/licenses/by/4.0/>). Please be aware that this license allows unrestricted use and distribution, even commercially, by third parties. Please reply and provide explicit written permission to publish XXX under a CC BY license and complete the attached form.”

 Please upload the completed Content Permission Form or other proof of granted permissions as an "Other" file with your submission.

 In the figure caption of the copyrighted figure, please include the following text: “Reprinted from [ref] under a CC BY license, with permission from [name of publisher], original copyright [original copyright year].”

- Thank you for your suggestion. We have decided to keep figure 1 with additional information, links for permission using caption “**Fig 1.** Tick sampling points within the selected districts (Chitwan, Kathmandu, and Rasuwa). The thick dark grey line represents the national boundary of Nepal, while lighter grey lines indicate administrative district boundaries. Elevation is shown using a color ramp derived from Shuttle Radar Topography Mission (SRTM) data. Country and administrative boundary shapefiles of Nepal were obtained from Open Knowledge Nepal ([https://localboundries.oknp.org/](https://localboundries.oknp.org/?utm_source=chatgpt.com)) under a CC BY 4.0 open license. SRTM elevation data were obtained from the United States Geological Survey (USGS/NASA), which are in the public domain (<https://www.usgs.gov/centers/eros/science/usgs-eros-archive-digital-elevation-shuttle-radar-topography-mission-srtm>). Figure created with QGIS software version 3.34, an open-source Geographic Information System (GIS) licensed under the GNU General Public License (<https://qgis.org>).”

 2. If you are unable to obtain permission from the original copyright holder to publish these figures under the CC BY 4.0 license or if the copyright holder’s requirements are incompatible with the CC BY 4.0 license, please either i) remove the figure or ii) supply a replacement figure that complies with the CC BY 4.0 license. Please check copyright information on all replacement figures and update the figure caption with source information. If applicable, please specify in the figure caption text when a figure is similar but not identical to the original image and is therefore for illustrative purposes only.

3. We note you have included a table to which you do not refer in the text of your manuscript. Please ensure that you refer to Table 5 in your text; if accepted, production will need this reference to link the reader to the Table.

- Thank you for suggestion. Now Table 5 is cited in text. (Line: 222).

4. If the reviewer comments include a recommendation to cite specific previously published works, please review and evaluate these publications to determine whether they are relevant and should be cited. There is no requirement to cite these works unless the editor has indicated otherwise.

- Thank you so much. References are added now only if they are more relevant while revising.

**Additional Editor Comments:**

Dear Authors,

The reviewers have completed their evaluation of your manuscript. I encourage you to revise and resubmit your work, ensuring that all reviewer comments are thoroughly addressed. Please incorporate the feedback carefully and provide a detailed, point-by-point response that clearly outlines every change made in response to the reviewers’ suggestions.

In addition, kindly correct all typographical and grammatical errors, and ensure that the manuscript is prepared in full compliance with the journal’s formatting and submission guidelines.
We look forward to receiving your revised submission. [Note: HTML markup is below. Please do not edit.]

- Thank you so much for opportunity to revise the manuscript. We have prepared our manuscript as per suggestions as much as possible and applicable.

**Reviewers' comments:**

**Reviewer's Responses to Questions**

**1. Is the manuscript technically sound, and do the data support the conclusions?**

The manuscript must describe a technically sound piece of scientific research with data that supports the conclusions. Experiments must have been conducted rigorously, with appropriate controls, replication, and sample sizes. The conclusions must be drawn appropriately based on the data presented.

Reviewer #1: Partly

Reviewer #2: No

Reviewer #3: Partly

Reviewer #4: Partly

**2. Has the statistical analysis been performed appropriately and rigorously?**

Reviewer #1: No

Reviewer #2: No

Reviewer #3: No

Reviewer #4: No

**3. Have the authors made all data underlying the findings in their manuscript fully available?**

The [PLOS Data policy](http://track.editorialmanager.com/CL0/http:%2F%2Fwww.plosone.org%2Fstatic%2Fpolicies.action%23sharing/1/010f01989a3f859b-773c37a0-945c-4a5e-8e1c-f83cb1be9d61-000000/zN6rUY5CiAzXYfy6yl-vwURNaExkYXm2xy64hFIb8FI=223) requires authors to make all data underlying the findings described in their manuscript fully available without restriction, with rare exception (please refer to the Data Availability Statement in the manuscript PDF file). The data should be provided as part of the manuscript or its supporting information or deposited to a public repository. For example, in addition to summary statistics, the data points behind means, medians and variance measures should be available. If there are restrictions on publicly sharing data e.g. participant privacy or use of data from a third party those must be specified.

Reviewer #1: Yes

Reviewer #2: Yes

Reviewer #3: No

Reviewer #4: No

**4. Is the manuscript presented in an intelligible fashion and written in standard English?**

PLOS ONE does not copyedit accepted manuscripts, so the language in submitted articles must be clear, correct, and unambiguous. Any typographical or grammatical errors should be corrected at revision, so please note any specific errors here.

Reviewer #1: No

Reviewer #2: Yes

Reviewer #3: Yes

Reviewer #4: No

- Thank you so much for responding to all the questions above and for the opportunity to revise this manuscript.

**5. Review Comments to the Author**

Please use the space provided to explain your answers to the questions above. You may also include additional comments for the author, including concerns about dual publication, research ethics, or publication ethics. (Please upload your review as an attachment if it exceeds 20,000 characters)

**Reviewer #1:**

Dear authors:

Your research on ticks associated with domestic animals in Nepal offers valuable insights into the study of the parasite-host relationship. However, I have several questions regarding the results, particularly the taxonomic identification of the ticks.

Firstly, as you mention in the text, determining the species of some of the ticks was not possible due to their stage of development (i.e., they were nymphs). Two genera (Hyalomma and Dermacentor) could not be specifically identified due to the absence of applicable keys. However, some species that are widely distributed in Nepal, such as Dermacentor everestianus, have recently been redescribed in all their developmental stages. While nymphs can be identified using taxonomic criteria, the characteristics required for identification are more complex to observe under microscopic examination.

- Thank you so much for your valuable suggestion. Although *Dermacentor everestianus* has been redescribed in detail (Apanaskevich et al., 2014), its identification relies on precise morphometric characters and features typically observed using electron microscopy, which are difficult to assess with stereo-zoom dissecting microscopy only, without specialized resources and comparative keys to distinguish one from other. Moreover, *D. everestianus* is mainly reported from high-altitude regions in summer, while our samples were collected from the rainy to winter seasons, So, instead of missing identification, we conservatively identified our specimens only to the genus level.

2) With regard to this matter, you indicate in the text that several nymphs were found in your study, but you are interested in calculating the sex ratio. Please clarify the rationale behind calculating the sex ratio for species identified at the genus level, given that the nymph and adult stages correspond to different developmental phases.

- Nymphs are immature and cannot be sexed reliably so, we have not calculated the sex ratio for nymphs in this study. We consider the fact that sex ratio are species specific and our study have some limitation, but we just wanted to give a general overview of population dynamics and disease transmission risk in those host and areas. (Line: 299-300).

Thirdly, you indicated that you were unable to identify some specimens because they were engorged females. This is not a valid reason for failing to identify a tick specimen. The characteristics can be observed, and there are methods to manipulate ticks so that they can be observed.

- Thank you for your comments. It’s not that we have not identified all the engorged females, it’s just few of them because their whole identification keys were not clear. However, during manipulation by pressing or dissecting the engorged female ticks were identified only up to genera (Line: 326-328).

4) The term "spp." is used in the genera that were identified. The term "spp." indicates the presence of multiple morphospecies of ticks within a particular genus. Did you find that to be the case? If not, please use the word "sp."

- Thank you for pointing this out. If the number of ticks identified up to genus level were more than one, then we used the term “spp.” considering their possibility of being multiple species under the common genera whereas the identified species contains only one individual then we used the term “sp.” this statement is validate by other previous studies <https://pmc.ncbi.nlm.nih.gov/articles/PMC3681633/> (Line: 136-139).

5) You indicate that your work does not necessitate an ethics statement, however, the guidelines for authors stipulate that the use of vertebrates requires this statement. As you have experience working with domestic animals, please indicate in the text whether the animals came from a herd managed by shepherds or from your university's facilities. In either case, your institution must have a Bioethics Committee that should have approved your work in advance.

- For this work, we collected ticks from individual households rearing animals, herd managed by shepherds and collected some ticks from university’s facilities as well. We got permission for this from the owners and University. We have included ethical approval from the University with the revised manuscript (Line: 117-120).

6) Please indicate in which scientific collection you plan to deposit the material from this work. Please specify this in the text.

- All species will be deposited in department of parasitology and microbiology at Agriculture and Forestry University.

7) Each photograph in Figure 2 must be accompanied by a scale bar. It is unclear whether the quality was compromised during the conversion process, but both the map and Figure 2 do not meet the journal's standards.

- The quality was little compromised during the conversion process, but we will try to attach the best possible figures we have.

Please consider these comments as opportunities for improvement in your future work. Please refer to the attached PDF for additional comments.

- Thank you so much for inputs. We have thoroughly addressed comments.

Best wishes for a successful outcome.

- Thank you so much for your valuable suggestions.

**Reviewer #2:**

Ethical concerns: Are the studies approved by Ethical Committee of the country? If yes, please provide the details about ethics. I think the country has veterinary ethical body regarding the study ethical clearance and authors should state if so.

- We got permission from the farmers and University for the study and have attached the ethical from the University with the manuscript (Line: 118-120).

Abstract: Total animals = 210, The sample size for the whole study areas at provincial level is very low.

- We screened many animals, but we could not include all of them in the study as many of them were not infested with ticks. We counted only infested animals in the study, leading to a smaller number of sample sizes (Line: 123).

66 Ectoparasites constitute a significant problem in most developing and underdeveloped countries
Which sentence/science/citation support this sentence?

- Thank you. Reference is added in the manuscript (Line: 70).

114 buffalo, cattle, sheep, goats, and dogs. The study was carried out from September 2023 to January
To collect so few samples, you are almost using two seasons; September last of rainy season and January, the winter season. How can you relate the results over the different periods?

- We collected the ticks mostly in winter season, so we will not interpret the results over different time period.

122 udder, back, and inside the tuft of the tail. Approximately 2 to 5 ticks were collected from each
You said you collected about 2 to 5 ticks only, it means, you are leaving other ticks.

- Some animals were severely infested, and some animals had only few of them. Collecting all the ticks from all animals is not practically applicable and may compromise animal welfare also. So, we choose to take representative 2-5 ticks depending on the severity of infestation. We have included references also (Line: 128).

140 Shannon-Wiener diversity index (H) = - Σ pi ln(pi)

If you are collecting 2 to 5 ticks only, then, how can H represents accurate values?

- We acknowledge the reviewer’s concern, collecting 2-5 ticks per animal may not fully represent the entire diversity on each host. However, the index was calculated across pooled samples from multiple hosts within each species and region, rather than focusing on per individual animal. Thus, it intends to provide a general estimate of tick diversity among animals and the study area.

142 Simpson’s diversity index (S)= Σ n(n-1)/N(N-1)

Also, the S values, how can it be assessed in each host?

- As there were limited ticks collected from each individual host, S values were not calculated for individual host. Rather, we calculated Simpson’s diversity index by collecting data from all individuals of certain animal species and this gave the diversity patterns across host population.

148 A total of 755 ticks were collected from 210 animals in Bagmati Province. It means, the numbers of ticks from 210 animals is very very low in this Province. I think the data will not be so much significant for the global readers. However, the low data is highly significant in the context of taxonomic data.

- Thank you for pointing this out.

173 (Haemaphysalis longicornis, Haemaphysalis sulcata, Haemaphysalis leachi, Haemaphysalis
174 spp.), Dermacentor spp., and Hyalomma spp. During identification, the main morphological
What does it mean by Hyalomma spp. and Haemaphysalis spp.?
181 Haemaphysalis leachi 2.05%, Dermacentor spp. 1.77%, Rhipicephalus spp. 1.36
Similar for Dermacentor spp. and Rhipicephalus spp.? It is difficult to explain rather than describe different species diversities, abundance based on a few samples of ticks collected.

- We identified some ticks up to genus level and they were different from other species that we identified. So, we tried to keep them separately as class.

Therefore, due to low sample collections, although the species identification needs further revision of the MS, especially regarding shortening of the MS. Also authors may make it significant in the context of pathology and explain why different organs/tissues are preferably infested by ticks. However, due to collection of equal/unequal numbers of ticks from few hosts, may make the wrong conclusions.
Thank you.

- Thank you for your comments and we have already explained the reasons for low and unequal sample collection.

**Reviewer #3:**

Comment 1:

Line no 210 Bagmati province (13 species), But in table it is 11 which one is correct

- Thank you for pointing out, it’s 11 species. We have made corrections in the manuscript (Line: 232).

Comment 2:

Actual species is 7. Other are spp. How the index calculated it is misleading.
The spp is not identified and cannot be included in analysis as it is considering as separate species.

- Even though some ticks were identified up to genus level, they were different species than what we have identified here. So, we thought of keeping them separately and counting them as well.

Comment 3:

Why the study is in winter, and author himself stated unfavorable.

- Thank you for your query. The time allocated for this research was six months only. Thus, we have time constraint for this study (Line: 344-347).

Comment 4:

What is sampling method. How many domestic animals were available, how many screened, and how many tick infested. There should be table for Tick infestation rate.

- We agree that it would be better if we included those things, but this research is more focused on the distribution and diversity of ticks rather than the prevalence of ticks/ infestation rate. We screened the animals and considered only the infested animals for the study.

Comment 5:

There is no statistical analysis. For scientific publication there is need of statistical analysis for tick infestation rate, tick abundance, and tick intensity. This will give actual burden of the ticks in the study area.

- Thank you so much for your concern. We were more focused on distribution of ticks so we did not consider these parameters.

Comment 6

The Shannon Wiener diversity index was higher for small ruminants (1.01), followed by dogs (0.877), and large ruminants (0.463) (Table 6). This showed more diversity of ticks among small ruminants and less diversity in large ruminants.

Wrong interpretation of index. Check reference.

- Shannon Wiener diversity index is higher in small ruminants suggesting higher diversity in small ruminants and the conclusion is supported by.(Moradi et al., 2021) and we have included the reference in the manuscript (Line: 151).

Comment 7

Of the identified species, how many are reported as vectors (zoonosis) transmit to human and domestic animals in Nepal. Give emphasis on this aspects,

- In our previous study, we detected *Haemaphysalis*, *Dermacentor*, and *Rhipicephalus* from dogs infected with *Babesia*, *Anaplasma,* *Ehrlichia canis*, and *Hepatozoon*, suggesting their potential role as vectors. While a few studies report the prevalence of tick-borne diseases in Nepal, there is a limited focus on the vector species and their role in transmission.

Comment 8

How Rhipicephalus decolaratus has been identified. It is very rare and difficult for identification.
Author can avoid sentence such as difficult to identify female and nymph. In the discussion that part of paragraph has to removed and it is common issue and it is not new research finding. This is species diversity paper and experienced taxonomist able to identify all stages of ticks. Line 292 to 301 statement is not true. Any statement should be given with published refererence. It will mislead the reader.

- Identification was done based on the guidelines mentioned in the paper line 135 and observed features like hexagonal basis capitula, presence of adanal and accessory adanal plates, caudal appendage, ventral spur etc.

Comment: 9

Constraints of the study Should be modified that the author itself not confidence.

- Thank you for your suggestion. We did not get chance to do PCR, so, we have included the point in the revised manuscript (Line: 345).

Comment 10

Line no 207. Total abundance of genera column is misleading. It has to be removed

- Thank you so much. We have removed that column.

Overall comment

It is basic work. Many statements are assumption rather than evidence.

- Thank you so much for complimenting us and revising opportunity with genuine feedback.

**Reviewer #4:**

Dear author,

Please find below the comments for this article.

PLOS ONE (PONE-D-25-27830). Species diversity and geographical distribution of ticks infesting domestic animals in Bagmati province, Nepal

Comments
1. Mention in details the year wise Tick borne diseases recorded in your area or the place of survey?

- Thank you for your genuine concern. However, there are not many studies in this field, and we are unable to reflect such precise data to depict the year-round details.

2. When this study was conducted season? and duration

- The study was conducted from September 2023 to January 2024, and it was mainly winter season (Line: 117-118).

3. Ethical clearance certificate?

- Thank you so much. We have included that in this revised manuscript from the Department of Microbiology and Parasitology to conduct research under the supervision of registered veterinarians (Line: 118-120).

4. Have you got field permission in the individual places?

- Thank you. Yes, we got permission and consent from the owners before sampling.

5. Line 103 On what basis you selected the areas of collection six different sampling areas and
please mention the number of Tick borne infection noticed in those areas

- The areas of study were selected based on the population of animals. Kathmandu is an urban area where people prefer to keep dogs as their companion and do not rear much of other animals. Rasuwa on the other hand is rural region and people depend on live stocks. While in Chitwan, we can find most of these animals.

6. Please mention the areas name individually with the elevation in different areas.?

- We have already included that in the text (Line: 109-113).

7. Mention any urban rural, semi urban and forested areas were included

- Yes, we have included such areas. Kathmandu and Chitwan are basically urban areas and Rasuwa come under semi urban/ rural areas (Line: 103-104).

8. Can you relate the available species as a vector of any TBD

- Yes, we detected 11 species of ticks and some of them like *Rhipicephalus microplus*, *Rhipicephalus sanguineus,* *Haemaphysalis longicornis* which are generally responsible for diseases causing agents: *Babesia, Ehrlichia, Rickettsia, Anaplasma, Theileria* etc. These diseases are also reported in the context of Nepal in recent reports. These could be responsible vectors in the context of Nepal.

9. line no. 86 Nepal, economic loss due to ticks and tick-borne diseases in livestock was reported to be 18.71%- Please mention that in detail.

- Details are added in the manuscript (Line: 81-84).

10. line 87 of the total cost [18] ?.

- Thank you so much. Actually ‘total cost’ refers to the total cost of livestock production (Line: 79-82).

11. 128,129 you followed a wet method or dry method for observation of ticks under the microscope?

- Thank you. Since we have ethanol preserved tick samples. Obviously, we followed dry method (Line: 133).

12. Have you collected ticks from domestic animals, fowls and cats?

- Thank you so much. It is clearly mentioned in the manuscript. However, for your information we only collected the tick samples collected from cattle, buffaloes, sheep, goats, and dogs (Line: 116-118-119).

13. What are the slide preparation procedures followed?

- Thank you for your queries. We mostly used stereo zoom microscope. We simply placed them on the slide and observed them.

14. Mention indices references?

- We have included that in paper (Line: 151).

15. 153 How did you find the age of the individual animal?

- Thank you for your nice comment. First, based on the farm records and history told by owner of animal we were able to find the age of animals. In addition to their records and we checked the dentition in some cases as well.

16. What is the criteria followed to delineate the less, moderate and high?- any reference?

- Thank you so much. The severity of tick infestations was also categorized as less (if <10 ticks/animal), moderate (10-50 ticks/animal), and high (>50 ticks/animal). Yes, we have added the reference (Line: 163-164).

17. 177 Can you mention the specific animal Which is mostly affected by ticks infestation?

- In our case, Sheep were mostly infested as we collected 215 ticks from them. (See Table 4).

18. 186,189, affected means Is there any clinical manifestation found on the animal?

- Thank you for pointing out. It’s infested, and we have made corrections in the revised paper (Line: 213).

19. 201 Hill high - what is the elevation?

- We have mentioned that in the paper in the Material and methods sections under subheading Study area (Line: 111-113)

20. 241 Is there any Tick-borne disease noticed on goats in those areas?

- Thank you. There are no such studies that reported tick-borne diseases specific to goats in our study sites.

21. 256,257 highlighting its significant role in the transmission of important pathogens in Central Nepal-m please explain in detail

- Based on the literature, *Rhipicephalus microplus* was found to be responsible for transmitting different diseases (we have mentioned the paper in our paper). *Rhipicephalus microplus* was found to be abundant in our study in that region and we wanted to highlight the possibility of these diseases in animals of that region as *Anaplasma bovis, Anaplasma marginale, Theileria annulata, Theileria orinetalis* pathogens were observed in cattle infested with the ticks and these things are mentioned in (Line: 251- 257).

22. What are the different diseases transmitted by this species in Nepal -Mention it with proper reference?

- There are few studies in ticks and tick-borne diseases in Nepal which we have included in the (Line: 251- 257).

23. In the discussion, please mention about the diseases transmitted by ticks in Nepal reported elsewhere and please mention about the ticks species already reported in Nepal as a small introduction.

- Thank you so much. We have mentioned in revised version (Line: 251- 257).

24. 238 Compare this study with the other studies in Nepal, for example: Utsav Lamichhane and Hom Bahadur Basnet, 2020.

- Thank you for your suggestion. Utsav Lamichhane and Hom Bahadur Basnet, 2020, they have studied ticks in cattle only and compared the results from two different seasons, but our study focuses on different species of animals and regions of Bagmati province. Majority of ticks observed were *Rhipicephalus microplus* in their study which is similar to this study. They also observed ticks like Ixodes, Amblyomma etc which were not found in the study. We have included these things in the revised paper (Line: 284-286).

25. How is this work similar to Kunwar 2022?

- Thank you for your queries. We have cited Kunwar et al. 2022 in the discussion section for the prevalence of tick on goat where Chitwan District is the common research site and the identified species of ticks were almost similar (Line: 266-267).

26. 240 Please mention in detail the tick species available during the favourable season from these sampled areas.

- This is one of the first research to identify ticks in those locations. We don’t have much literature about the ticks in the sampled areas. One of the studies by Aryal et al. 2025 found *Rhipicephalus*, *Haemophysalis* and *Dermacento*r in dogs of Chitwan and Kathmandu.
- *Rhipicephalus microplus* in cattle of Chitwan District (Pandey et al. 2025), *Rhipicephalus*, *Amblyomma, Ixodes* in cattle of Chitwan in different seasons (Lamichhane and Hom Bahadur Basnet, 2020). These all are mentioned in (Line: 251-257).

27. 241 Is there any of the Anaplasma, Babesia, and Theileria infection reported in goats in Nepal?

- Yes. There is one document regarding blood parasites in goats in the eastern Terai regions of Nepal (Line: 276-278).

28. 246-248 Please mention the tick-borne diseases reported from these areas.

- Thank you. The tick-borne disease specific to the goat are still lacking in our study areas, however the future detection of such disease-causing pathogens cannot be neglected (Line: 277-278).

29. 257 Its significant role in the transmission of important pathogens in Central Nepal - please mention the reference to substantiate your claim.

- Yes, we have added the reference (Line: 251-257).

30. Have you done any screening to find out the pathogens present in these ticks to prove your statements?

- No, we have not worked in this field.

31. 264-265 Can you cite any reference to support your claim?

- Thank you so much. We have added three references in the given statement/claim (Line: 284).

32. After studying tick populations for one or two years from this area during different seasons, you can mention seasonal variation patterns.

- Thank you so much for suggestions. As we have limited time for this research, we were unable to include the sample for the whole year around. So, we did not exaggerate this parameter in the manuscript and included in the constraints of our study (Line: 336-337).

33. 286 Please mention in detail about the pathogens present in Terai region

- The pathogens present in terai are *Babesia, Borrelia*, *Anaplasma*, *Theileria*, *E.canis* and many of them are included in manuscript (Line: 251- 257).

34. 292-294 What are you going to do with unidentified samples?

- We have preserved those unidentified samples for PCR in future. However, this time we have provided some recommendations to overcome such research constraints (Line: 338-340).

35. 299-300 Collection method is to be changed please use the standard protocol

- Standard protocols for tick collection include the flagging-dragging, carbon dioxide traps, or host-based collection (Line: 336-337).

36. This study is conducted in a very short period, this should be at least conducted for a year to get a clear picture from this place

- We appreciate your feedback, but we had limited time of six months research internship to conduct the research.

37. In figure 1, please mention the name of the individual areas.

Thank you so much. Samples were collected from six different sampling Municipalities: Kathmandu Metropolitan City and Kritipur Municipality representing the Kathmandu District (mid hill), Ratnanagar Municipality and Bharatpur Metropolitan City representing Chitwan District (plain), and Dhunche Municipality and Aamachhodingmo Rural Municipality representing Rasuwa (high hill). Ratnanagar, Rampur, Phurtichok, Khsetripur, Devghat were the sites from Chitwan District, Chovar, Sanepa, Kritipur, and Kalanki were the sampling sites in Kathmandu District and Dhunche and Chilime from Rasuwa District (Fig 1) (Line: 99-107).

38. Ref 18 title should be given in the reference

- Thank you so much for your suggestion. We have modified accordingly (Line: 438).

39. All the references should be modified as per the journal format

- Thank you for genuine concern. We have followed the journal format.

40. In this article, no statistical outputs were expressed. The following statistical points are required to include.
41. In this article, GIS Choropleth Maps (empowered by ESRI), as Figure 2, are required to include.

- Yes, we have included the Map with details, links to access, and permissions.

42. In which criteria was age calculation made for the host animals grouping (i.e., grouped into three age groups as <1, 1-3, and > 3), and severity of Tick infestation (i.e., grouped into low, moderate and high)?

- Animals were categorized into the three age groups to make inferences for epidemiological relevance and susceptibility of tick infestations i.e. whether young, adults, or old-aged animals (Line: 168-171).
- In addition, the severity of tick infestations was also categorized as less (if <10 ticks/animals), moderate (10-50 ticks/animal) and severe (>50 ticks/animals) (Line: 170-171).

6. **PLOS authors have the option to publish the peer review history of their article (**[**what does this mean?**](https://track.editorialmanager.com/CL0/https:%2F%2Fjournals.plos.org%2Fplosone%2Fs%2Feditorial-and-peer-review-process%23loc-peer-review-history/1/010f01989a3f859b-773c37a0-945c-4a5e-8e1c-f83cb1be9d61-000000/6ZI8Y670CBz8ZSxtFawje_t6VoqP04hpbh40IxZ4JAw=223)**). If published, this will include your full peer review and any attached files.**

If you choose “no”, your identity will remain anonymous but your review may still be made public.

**Do you want your identity to be public for this peer review?** For information about this choice, including consent withdrawal, please see our [Privacy Policy](https://track.editorialmanager.com/CL0/https:%2F%2Fwww.plos.org%2Fprivacy-policy/1/010f01989a3f859b-773c37a0-945c-4a5e-8e1c-f83cb1be9d61-000000/rXlP7V5LZNHX8-RFCjT7hPdtgiPjfmAAaGi5dbZOvCY=223).

Reviewer #1: **Yes:** Angel Herrera-Mares

Reviewer #2: No

Reviewer #3: No

Reviewer #4: No

[NOTE: If reviewer comments were submitted as an attachment file, they will be attached to this email and accessible via the submission site. Please log into your account, locate the manuscript record, and check for the action link "View Attachments". If this link does not appear, there are no attachment files.]

While revising your submission, please upload your figure files to the Preflight Analysis and Conversion Engine (PACE) digital diagnostic tool, [https://pacev2.apexcovantage.com/](https://track.editorialmanager.com/CL0/https:%2F%2Fpacev2.apexcovantage.com%2F/1/010f01989a3f859b-773c37a0-945c-4a5e-8e1c-f83cb1be9d61-000000/TUMNI35a_tRxaepg2Yfxb8T23zOfIf58O87bZVHU4lI=223). PACE helps ensure that figures meet PLOS requirements. To use PACE, you must first register as a user. Registration is free. Then, login and navigate to the UPLOAD tab, where you will find detailed instructions on how to use the tool. If you encounter any issues or have any questions when using PACE, please email PLOS at [figures@plos.org](mailto:figures@plos.org). Please note that Supporting Information files do not need this step.
