# Supplementary material for: Species diversity and geographical distribution of ticks infesting domestic animals in Bagmati Province, Nepal
Source: PLoS One. 2026 Jun 8;21(6):e0351151. doi: 10.1371/journal.pone.0351151 (PMC13245791; doi:10.1371/journal.pone.0351151)
Supplement: Attachment — Submitted filename: comments 121.2026.docx [file pone.0351151.s006.docx]

This is a well written manuscript on tick diversity & distribution in Bagmati, Nepal. The study is well structured, methods are clearly described and results are presented. Some suggestion for improvement, from minor correction to more substantive recommendation for strengthening the discuss and clarity.

Abstract and summary: the abstract mention Rhipicephalus parva (0.14%), but in table 3 it appears as Rhipicephalus parva (14%)-this is typo that must be corrected

Summary could be slightly expanded to explicitly state the zoonotic implications and public health relevance.

Ensure italicization of all genus/species names throughout (Rhipicephalus, Haemaphysalis, etc.).

In Table 6, ensure column headings are clear (“Large Ruminants”, “Small Ruminants”, “Dogs”).

Reference formatting: ensure all in-text citations match the reference list (not provided here)

Line no 26–27	Specify that Rhipicephalus (Boophilus) microplus is a hard tick for clarity.

Line no 35–36	Correct “Rhipicephalus parva (14%)” to “Rhipicephalus parva (0.14%)” to match Table 3.

Line no 138–139 Clarify: “spp.” for multiple species under a genus, “sp.” for a single unidentified species.

Line no 163–164 Cite reference for infestation severity categories or justify your classification.

Line no 176–178 Specify that nymphs were excluded from sex ratio calculations.

Line no 201–206 Consider adding a brief sentence on why R. microplus might be so abundant (e.g., host availability, climate suitability).

Line no 252 Check consistency in spelling: “Dermacentor” (not Dermacenter).

Line no 289 Change “significant role” to “potential role” unless supported by pathogen detection data.

Line no 313-314 Explicitly state that winter sampling may underestimate tick diversity.

Line no 326–332 This paragraph is important—consider moving it to the Constraints section for better flow.

Line no 344–347 Expand slightly: e.g., Molecular confirmation - species identification accuracy.

Line no 350–355 In conclusions, explicitly recommend integrated tick management in high-diversity areas like Chitwan.

Overall recommendation- Suitable for publication after revisions. The study delivers useful baseline data on tick ecology in Nepal and identifies high-risk areas and hosts. Expanding the discussion on zoonotic risks and control would enhance its significance.
