# Supplementary material for: Species diversity and geographical distribution of ticks infesting domestic animals in Bagmati Province, Nepal
Source: PLoS One. 2026 Jun 8;21(6):e0351151. doi: 10.1371/journal.pone.0351151 (PMC13245791; doi:10.1371/journal.pone.0351151)
Supplement: Attachment — Submitted filename: Response to reviewers (2).docx [file pone.0351151.s008.docx]

**Response to the editor and reviewers**

**PONE-D-25-27830R1**

Species diversity and geographical distribution of ticks infesting domestic animals in Bagmati province, Nepal

ACADEMIC EDITOR:

I want to express my sincere gratitude for your consideration. I have some reservations regarding the taxonomic identity of the nymphs. The authors must ensure that they are working with a single morphospecies or more. Should they elect to undertake a microscopic review of all nymphs, they will be able to verify this.

It is imperative to note that the calculation of diversity indices necessitates the accurate identification of species. Because of that, I recommended to the authors that they remove the nymph results from the calculation of indices and focus only on tick species, i.e., those identified with genus and specific epithet.

For these reasons, I believe that the manuscript requires some minor revisions.

Thank you for your suggestions. We could not perform taxonomic identification of nymphs so, we have not included nymph for the diversity indices calculation. Also, *Rhipicephalus spp.* and *Haemaphysalis spp.* had more than one morphospecies of ticks so, we removed them from the diversity index calculation.

Please submit your revised manuscript by Mar 16 2026 11:59PM. If you will need more time than this to complete your revisions, please reply to this message or contact the journal office at plosone@plos.org. When you're ready to submit your revision, log on to https://www.editorialmanager.com/pone/ and select the 'Submissions Needing Revision' folder to locate your manuscript file.

Please include the following items when submitting your revised manuscript:

A letter that responds to each point raised by the academic editor and reviewer(s). You should upload this letter as a separate file labeled 'Response to Reviewers'.

A marked-up copy of your manuscript that highlights changes made to the original version. You should upload this as a separate file labeled 'Revised Manuscript with Track Changes'.

An unmarked version of your revised paper without tracked changes. You should upload this as a separate file labeled 'Manuscript'.

If you would like to make changes to your financial disclosure, please include your updated statement in your cover letter. Guidelines for resubmitting your figure files are available below the reviewer comments at the end of this letter.

Thank you for giving us the chance to go through our manuscript and make some improvements based on the feedback and suggestions from the editor and reviewers.

Here is our response to reviewer letter where we have tried to go through all the comments and provide point to point response and improve our manuscript.

Bullet points with texts in red in this letter represent our response and the line numbers are included in the parentheses which help to track the changes that we have made in the manuscript.

Changes in the manuscript are also marked with different colors, and we have uploaded both revised version of the manuscript with markings and a clean version of the manuscript.

If applicable, we recommend that you deposit your laboratory protocols in protocols.io to enhance the reproducibility of your results. Protocols.io assigns your protocol its own identifier (DOI) so that it can be cited independently in the future. For instructions see: https://journals.plos.org/plosone/s/submission-guidelines#loc-laboratory-protocols. Additionally, PLOS ONE offers an option for publishing peer-reviewed Lab Protocol articles, which describe protocols hosted on protocols.io. Read more information on sharing protocols at https://plos.org/protocols?utm_medium=editorial-email&utm_source=authorletters&utm_campaign=protocols

Journal Requirements:

If the reviewer comments include a recommendation to cite specific previously published works, please review and evaluate these publications to determine whether they are relevant and should be cited. There is no requirement to cite these works unless the editor has indicated otherwise.

Please review your reference list to ensure that it is complete and correct. If you have cited papers that have been retracted, please include the rationale for doing so in the manuscript text, or remove these references and replace them with relevant current references. Any changes to the reference list should be mentioned in the rebuttal letter that accompanies your revised manuscript. If you need to cite a retracted article, indicate the article’s retracted status in the References list and also include a citation and full reference for the retraction notice.

Thank you so much. All the references have been updated as per the journal’s guidelines.

[Note: HTML markup is below. Please do not edit.]

Reviewers' comments:

Reviewer's Responses to Questions

Comments to the Author

1. If the authors have adequately addressed your comments raised in a previous round of review and you feel that this manuscript is now acceptable for publication, you may indicate that here to bypass the “Comments to the Author” section, enter your conflict of interest statement in the “Confidential to Editor” section, and submit your "Accept" recommendation.

Reviewer #1: All comments have been addressed

Reviewer #2: (No Response)

Reviewer #3: All comments have been addressed

Reviewer #4: All comments have been addressed

2. Is the manuscript technically sound, and do the data support the conclusions?

The manuscript must describe a technically sound piece of scientific research with data that supports the conclusions. Experiments must have been conducted rigorously, with appropriate controls, replication, and sample sizes. The conclusions must be drawn appropriately based on the data presented.

Reviewer #1: Partly

Reviewer #2: Yes

Reviewer #3: No

Reviewer #4: Yes

3. Has the statistical analysis been performed appropriately and rigorously?

Reviewer #1: No

Reviewer #2: Yes

Reviewer #3: No

Reviewer #4: No

4. Have the authors made all data underlying the findings in their manuscript fully available?

The PLOS Data policy requires authors to make all data underlying the findings described in their manuscript fully available without restriction, with rare exception (please refer to the Data Availability Statement in the manuscript PDF file). The data should be provided as part of the manuscript or its supporting information, or deposited to a public repository. For example, in addition to summary statistics, the data points behind means, medians and variance measures should be available. If there are restrictions on publicly sharing data—e.g. participant privacy or use of data from a third party—those must be specified.

Reviewer #1: Yes

Reviewer #2: Yes

Reviewer #3: No

Reviewer #4: Yes

5. Is the manuscript presented in an intelligible fashion and written in standard English?

PLOS ONE does not copyedit accepted manuscripts, so the language in submitted articles must be clear, correct, and unambiguous. Any typographical or grammatical errors should be corrected at revision, so please note any specific errors here.

Reviewer #1: Yes

Reviewer #2: Yes

Reviewer #3: Yes

Reviewer #4: Yes

6. Review Comments to the Author

Please use the space provided to explain your answers to the questions above. You may also include additional comments for the author, including concerns about dual publication, research ethics, or publication ethics. (Please upload your review as an attachment if it exceeds 20,000 characters)

Thank you so much for your time and effort to review and giving us the opportunity to revise the manuscript.

**Reviewer #1: Dear authors:**

I would like to thank you for taking my comments into consideration. However, I have some remaining reservations about your use of the "sp." and "spp." terms. It is important to note that the application of these taxonomical terms to species at the individual level is incorrect. Even in the reference provided, the terms are explained at the species level rather than the individual level. Due to the aforementioned points, I have some concerns about the calculation of both Diversity Indices. As you have identified nymphs, and are uncertain as to the number of species present, the values are likely to be understimated. This may result in discussions and conclusions that are not fully accurate.

I am aware that certain financial constraints may be an obstacle to accessing certain facilities, such as SEM, which are necessary for the diagnostic characteristics required to identify nymphs at the species level. However, a thorough review of the nymphs under the stereoscopic microscope is recommended to ensure the presence of one or more mophospecies.

I believe this is a vital aspect of your work. One alternative approach is to eliminate the nymphs from your analyses and focus solely on tick species (genus and specific epithet). Please note that I would not advise removing your nymphs results from the manuscript, but rather from the Shannon and Simpson Indices.

Best wishes

Thank you for your valuable comments and suggestions. We have not included nymphs in our diversity indices calculations, and we have included that point in the manuscript as well. (139)

**Reviewer #2:**

1. You have shown the difference between spp. and sp.Thank you. It means there are more than 11 species you discovered. Please explain accordingly.

Yes. But we have not included them in the calculation. We have not used nypmh data to calculate the diversity indices. Again, few adult ticks also had some missing parts which we discarded. At last, there were some ticks which were not identified upto species level and they could not be categorized as one morphospecies. Hence, we did not use them to calculate our indices. This is one of the major contraints of this study, because to further confirm the ticks upto species level, we could not do the molecular identification due to limited resources.

2. You indicated different diversities of ticks geographically. Why and how does those diversities occur in the geographies? You calculated those values. But what is the significance of those values? What is the current situation of such diversity? Need to be discussed and explained in the MS. Does this depend on host numbers or host age/sexes?

Differences in the diversity of ticks based on geography can be best described based on the climate (temperature, humidity). Since we did sampling in 3 different climatic zone ranging from subtropical to alpine regions as described in line 109-114, difference in diversity was observed.

Higher Shannon Wiener diversity value suggests higher diversity in that geographical region. This suggests that there is presence of multiple species with relatively even distribution and there is higher risk of multiple tick-borne pathogens in that region. Hence, the diversity values are important.

There are limited papers and data on diversity in those areas so we cannot say about the exact current situation right now.

Yes, it does depend on the host related factors like age, sex and numbers. Rather, susceptibility of animals to tick infestation differs from individual to individual based on their age, sex, environment, management practice etc. Young animals may have lower exposure to ticks compared to older ones due to shorter environmental contact. Taking few hosts may underestimate the diversity as less abundant species may not be detected. Different tick species also exhibit host preferences, which can influence the diversity observed in a host population.

3. You said: '' This might be due to the greater number of 320 domestic hosts involved in the study in Terai under favorable environmental conditions.

How can you evidently say greater number of domestic hosts invovled in the study of Terai under favorable environmental conditions

The number of animals used in the study was more from Terai compared to other regions. The line is corrected in the manuscript. (318-319)

4. Again, please include the new updates of conclusion. Ticks are very neglected arthropods in the Asia especially, Nepal and neighboring countries.

Thank you so much. We have updated that in our study. (349-350)

5. Table 5. Geographical distribution of ticks in different locations

You collected tick specimen and pooled. It may result in the collection and pooling values biased. How did you reduce or nullify the biasness. That is why at your last paragraph, you should clearly state that this is biased study, however, it is qualitative

Thank you for your comment. We have already included that in constraints of study

You should write in the legend the total number of hosts studied in each geographic site.Thank you for your suggestion. We have included that information in supplementary file.

6. Table 6

Please add numbers of hosts studied in the legend. Also in hte province, district, host-wise

Thank you. We have included the host information in supplementary file.

7. Table 3

How did you achieve the abundance? what is the number for each species?

Total abundance is the total number of species and abundance percentage was calculated by dividing the total abundance of that species by total number of ticks and multiplying by 100. (149-150)

8. Table 1:

≤ 1yr 1–3 yrs. ≥3yrs

I did not understand your grouping. In which basis did you do that? any scientiific basis?

less than or equal to 1 year in one group

one to 3 year animal means 1 year is already in previous group.

equal to 3 or more than 3 means 3 is already included in the later group.

How is it possible?

Thank you for pointing that out. We divided the groups as <1 yr, 1-3 yrs, and >3 yrs and we have corrected that in our manuscript as well. (174)

Others:

Most of the references are not properly written. Please sort out this if possible.

Thank you. We have corrected that.

Reviewer #3: The reply to the reviewer is not satisfactory. We did not recommend this study in the plos level journal. Sample size is small.

Thank you for your comment. We did check other animals as well but only tick infested animals were included in our study. Since it was mostly conducted during winter season, many of the animals were not infested and we could not include them in our study.

Reviewer #4: This is a well written manuscript on tick diversity & distribution in Bagmati, Nepal. The study is well structured, methods are clearly described and results are presented. Some suggestion for improvement, from minor correction to more substantive recommendation for strengthening the discuss and clarity.

Abstract and summary: the abstract mention Rhipicephalus parva (0.14%), but in table 3 it appears as Rhipicephalus parva (14%)-this is typo that must be corrected

Summary could be slightly expanded to explicitly state the zoonotic implications and public health relevance.

Ensure italicization of all genus/species names throughout (Rhipicephalus, Haemaphysalis, etc.).

In Table 6, ensure column headings are clear (“Large Ruminants”, “Small Ruminants”, “Dogs”).

Reference formatting: ensure all in-text citations match the reference list (not provided here)

Line no 26–27 Specify that Rhipicephalus (Boophilus) microplus is a hard tick for clarity.

Line no 35–36 Correct “Rhipicephalus parva (14%)” to “Rhipicephalus parva (0.14%)” to match Table 3.

Line no 138–139 Clarify: “spp.” for multiple species under a genus, “sp.” for a single unidentified species.

Line no 163–164 Cite reference for infestation severity categories or justify your classification.

Line no 176–178 Specify that nymphs were excluded from sex ratio calculations.

Line no 201–206 Consider adding a brief sentence on why R. microplus might be so abundant (e.g., host availability, climate suitability).

Line no 252 Check consistency in spelling: “Dermacentor” (not Dermacenter).

Line no 289 Change “significant role” to “potential role” unless supported by pathogen detection data.

Line no 313-314 Explicitly state that winter sampling may underestimate tick diversity.

Line no 326–332 This paragraph is important—consider moving it to the Constraints section for better flow.

Line no 344–347 Expand slightly: e.g., Molecular confirmation - species identification accuracy.

Line no 350–355 In conclusions, explicitly recommend integrated tick management in high-diversity areas like Chitwan.

Overall recommendation- Suitable for publication after revisions. The study delivers useful baseline data on tick ecology in Nepal and identifies high-risk areas and hosts. Expanding the discussion on zoonotic risks and control would enhance its significance.

Thank you for your suggestion. We have included all the things mentioned above and made all necessary changes in the manuscript.

7. PLOS authors have the option to publish the peer review history of their article (what does this mean?). If published, this will include your full peer review and any attached files.

If you choose “no”, your identity will remain anonymous but your review may still be made public.

Do you want your identity to be public for this peer review? For information about this choice, including consent withdrawal, please see our Privacy Policy.

Reviewer #1: No

Reviewer #2: Yes: Tirth Raj Ghimire

Reviewer #3: No

Reviewer #4: No
