# Supplementary material for: Species diversity and geographical distribution of ticks infesting domestic animals in Bagmati Province, Nepal
Source: PLoS One. 2026 Jun 8;21(6):e0351151. doi: 10.1371/journal.pone.0351151 (PMC13245791; doi:10.1371/journal.pone.0351151)
Supplement: Attachment — Submitted filename: Response to Reviewers .docx [file pone.0351151.s009.docx]

PONE-D-25-27830R2

Species diversity and geographical distribution of ticks infesting domestic animals in Bagmati province, Nepal

PLOS One

Dear Dr. Thapa,

Thank you for submitting your manuscript to PLOS ONE. After careful consideration, we feel that it has merit but does not fully meet PLOS ONE’s publication criteria as it currently stands. Therefore, we invite you to submit a revised version of the manuscript that addresses the points raised during the review process.

==============================

ACADEMIC EDITOR:

While the manuscript has been improved, there are still some concerns about its suitability for PLOS One. I suggest a minor revision at this stage. I would like to express my concerns regarding the failure to identify engorged females. It should be noted that the authors have classified some species under that genera (Rhipicephalus and Haemapysalis). They have expanded their knowledge of tick taxonomy and taxonomy in general, and they have avoided the misuse of the words "spp." and "sp."

==============================

Thank you for your comment. We also identified engorged female tick which you can see in the results section in sex ratio statistics. The confusion occurred from the failure of explaining the limitation of identification of highly engorged females with or without intact mouth parts and other morphological traits. We wanted to explain that, only those highly engorged females which lack mouthparts or lack other features were not identified. Some of them were only identified up to genus level. We have clearly stated this in line 325 to 333 of the manuscript.

Please submit your revised manuscript by Jun 17 2026 11:59PM. If you will need more time than this to complete your revisions, please reply to this message or contact the journal office at plosone@plos.org. When you're ready to submit your revision, log on to https://www.editorialmanager.com/pone/ and select the 'Submissions Needing Revision' folder to locate your manuscript file.

Please include the following items when submitting your revised manuscript:

A letter that responds to each point raised by the academic editor and reviewer(s). You should upload this letter as a separate file labeled 'Response to Reviewers'.

A marked-up copy of your manuscript that highlights changes made to the original version. You should upload this as a separate file labeled 'Revised Manuscript with Track Changes'.

An unmarked version of your revised paper without tracked changes. You should upload this as a separate file labeled 'Manuscript'.

If you would like to make changes to your financial disclosure, please include your updated statement in your cover letter. Guidelines for resubmitting your figure files are available below the reviewer comments at the end of this letter.

If applicable, we recommend that you deposit your laboratory protocols in protocols.io to enhance the reproducibility of your results. Protocols.io assigns your protocol its own identifier (DOI) so that it can be cited independently in the future. For instructions see: https://journals.plos.org/plosone/s/submission-guidelines#loc-laboratory-protocols. Additionally, PLOS ONE offers an option for publishing peer-reviewed Lab Protocol articles, which describe protocols hosted on protocols.io. Read more information on sharing protocols at https://plos.org/protocols?utm_medium=editorial-email&utm_source=authorletters&utm_campaign=protocols.

As the corresponding author, your ORCID iD is verified in the submission system and will appear in the published article. PLOS supports the use of ORCID, and we encourage all coauthors to register for an ORCID iD and use it as well. Please encourage your coauthors to verify their ORCID iD within the submission system before final acceptance, as unverified ORCID iDs will not appear in the published article. Only the individual author can complete the verification step; PLOS staff cannot verify ORCID iDs on behalf of authors.

We look forward to receiving your revised manuscript.

Kind regards,

Faham Khamesipour, Ph.D.

Academic Editor

PLOS One

Journal Requirements:

If the reviewer comments include a recommendation to cite specific previously published works, please review and evaluate these publications to determine whether they are relevant and should be cited. There is no requirement to cite these works unless the editor has indicated otherwise.

Please review your reference list to ensure that it is complete and correct. If you have cited papers that have been retracted, please include the rationale for doing so in the manuscript text, or remove these references and replace them with relevant current references. Any changes to the reference list should be mentioned in the rebuttal letter that accompanies your revised manuscript. If you need to cite a retracted article, indicate the article’s retracted status in the References list and also include a citation and full reference for the retraction notice.

[Note: HTML markup is below. Please do not edit.]

Reviewers' comments:

Reviewer's Responses to Questions

Comments to the Author

1. If the authors have adequately addressed your comments raised in a previous round of review and you feel that this manuscript is now acceptable for publication, you may indicate that here to bypass the “Comments to the Author” section, enter your conflict of interest statement in the “Confidential to Editor” section, and submit your "Accept" recommendation.

Reviewer #1: All comments have been addressed

Reviewer #2: All comments have been addressed

Reviewer #4: All comments have been addressed

2. Is the manuscript technically sound, and do the data support the conclusions?

The manuscript must describe a technically sound piece of scientific research with data that supports the conclusions. Experiments must have been conducted rigorously, with appropriate controls, replication, and sample sizes. The conclusions must be drawn appropriately based on the data presented.

Reviewer #1: Partly

Reviewer #2: Yes

Reviewer #4: Partly

3. Has the statistical analysis been performed appropriately and rigorously?

Reviewer #1: Yes

Reviewer #2: Yes

Reviewer #4: I Don't Know

4. Have the authors made all data underlying the findings in their manuscript fully available?

The PLOS Data policy requires authors to make all data underlying the findings described in their manuscript fully available without restriction, with rare exception (please refer to the Data Availability Statement in the manuscript PDF file). The data should be provided as part of the manuscript or its supporting information, or deposited to a public repository. For example, in addition to summary statistics, the data points behind means, medians and variance measures should be available. If there are restrictions on publicly sharing data—e.g. participant privacy or use of data from a third party—those must be specified.

Reviewer #1: Yes

Reviewer #2: Yes

Reviewer #4: Yes

5. Is the manuscript presented in an intelligible fashion and written in standard English?

PLOS ONE does not copyedit accepted manuscripts, so the language in submitted articles must be clear, correct, and unambiguous. Any typographical or grammatical errors should be corrected at revision, so please note any specific errors here.

Reviewer #1: No

Reviewer #2: Yes

Reviewer #4: (No Response)

We have also tried to avoid the grammatical errors and improve the manuscript. We made some changes in text to be more clear, correct, and unambiguous in manuscript without changing their meaning regarding results, discussion and content.

6. Review Comments to the Author

Please use the space provided to explain your answers to the questions above. You may also include additional comments for the author, including concerns about dual publication, research ethics, or publication ethics. (Please upload your review as an attachment if it exceeds 20,000 characters)

Reviewer #1: Dear authors:

I appreciate your consideration of my feedback. It seems that your paper has undergone significant improvement. There are a few points that require your attention. I have some concerns about not identifying the engorged females. This is because certain species are suitable for comparison, particularly in the cases of Rhipicephalus and Haemaphysalis. I appreciate your efforts to learn more about tick taxonomy, which will help prevent the misuse of spp. and sp.

Thank you so much for your response.

We did the identification of engorged females as well. But, in the discussion part, the sentence was unclear. We wanted to disclose that, some highly engorged female which may or may not have intact mouth parts & morphological keys, were not identified even upto genus level.

Also, we have been very careful while handling the engorged ticks. Due to their high engorgement, while collecting samples some ticks lost their mouthparts. While examination, we still can identify them as female based on the scutum, but due to the lack other features, we could not identify them. That’s why we are disclosing this in our article to avoid any bias. But we had some weakness in the describing this in the manuscript. We are thankful for your response which helped us to review and make change. In some, engorged female tick, which had intact mouthparts, we were able to identify them upto Genus level based on the taxonomic keys that we mentioned in the manuscript but not up to species level.

We have changed line 325 to 333 to address this issue in manuscript.

-Please note that the words "sp." and "spp." should not be italicized along the manuscript.

-Please direct your attention to lines 125 to 126. These are the results. Please remove the sentence "Approximately 2 to 5 ticks were collected from each animal (20,21)."

-Please note that "ticks" on line 251 should not be italicized.

We had addressed these grammatical issues as well in the manuscript.

Best regards and best of luck with your paper.

Reviewer #2: Thank you Authors for your nice works. The article is now beautiful to get published in this journal.

Congratulations!

Reviewer #4: (No Response)

7. PLOS authors have the option to publish the peer review history of their article (what does this mean?). If published, this will include your full peer review and any attached files.

If you choose “no”, your identity will remain anonymous but your review may still be made public.

Do you want your identity to be public for this peer review? For information about this choice, including consent withdrawal, please see our Privacy Policy.

Reviewer #1: No

Reviewer #2: No

Reviewer #4: Yes: Dr. Renu Govindarajan

[NOTE: If reviewer comments were submitted as an attachment file, they will be attached to this email and accessible via the submission site. Please log into your account, locate the manuscript record, and check for the action link "View Attachments". If this link does not appear, there are no attachment files.]

To ensure your figures meet our technical requirements, please review our figure guidelines: https://journals.plos.org/plosone/s/figures

You may also use PLOS’s free figure tool, NAAS, to help you prepare publication quality figures: https://journals.plos.org/plosone/s/figures#loc-tools-for-figure-preparation.

NAAS will assess whether your figures meet our technical requirements by comparing each figure against our figure specifications.
