# Supplementary material for: Species diversity and geographical distribution of ticks infesting domestic animals in Bagmati Province, Nepal
Source: PLoS One. 2026 Jun 8;21(6):e0351151. doi: 10.1371/journal.pone.0351151 (PMC13245791; doi:10.1371/journal.pone.0351151)
Supplement: Peer review history

# Author comment: Author response to Decision Letter 0

**DOI:** 10.1371/journal.pone.0351151.r001

**Transfer Alert**

This paper was transferred from another journal. As a result, its full editorial history (including decision letters, peer reviews and author responses) may not be present.

22 May 2025

# Peer review: Decision Letter 0

**DOI:** 10.1371/journal.pone.0351151.r002

**Authors:** Bersissa Kumsa

11 Aug 2025

PONE-D-25-27830

Species diversity and geographical distribution of ticks infesting domestic animals in Bagmati province, Nepal

PLOS ONE

Dear Dr. Thapa,

Thank you for submitting your manuscript to PLOS ONE. After careful consideration, we feel that it has merit but does not fully meet PLOS ONE’s publication criteria as it currently stands. Therefore, we invite you to submit a revised version of the manuscript that addresses the points raised during the review process.

Please submit your revised manuscript by Sep 25 2025 11:59PM. If you will need more time than this to complete your revisions, please reply to this message or contact the journal office at plosone@plos.org. When you're ready to submit your revision, log on to https://www.editorialmanager.com/pone/ and select the 'Submissions Needing Revision' folder to locate your manuscript file.

Please include the following items when submitting your revised manuscript:

- A rebuttal letter that responds to each point raised by the academic editor and reviewer(s). You should upload this letter as a separate file labeled 'Response to Reviewers'.
- A marked-up copy of your manuscript that highlights changes made to the original version. You should upload this as a separate file labeled 'Revised Manuscript with Track Changes'.
- An unmarked version of your revised paper without tracked changes. You should upload this as a separate file labeled 'Manuscript'.

If you would like to make changes to your financial disclosure, please include your updated statement in your cover letter. Guidelines for resubmitting your figure files are available below the reviewer comments at the end of this letter.

If applicable, we recommend that you deposit your laboratory protocols in protocols.io to enhance the reproducibility of your results. Protocols.io assigns your protocol its own identifier (DOI) so that it can be cited independently in the future. For instructions see: https://journals.plos.org/plosone/s/submission-guidelines#loc-laboratory-protocols. Additionally, PLOS ONE offers an option for publishing peer-reviewed Lab Protocol articles, which describe protocols hosted on protocols.io. Read more information on sharing protocols at https://plos.org/protocols?utm_medium=editorial-email&utm_source=authorletters&utm_campaign=protocols.

We look forward to receiving your revised manuscript.

Kind regards,

Bersissa Kumsa, DVM, MSc, PhD

Academic Editor

PLOS ONE

Journal Requirements:

When submitting your revision, we need you to address these additional requirements.

1.Please ensure that your manuscript meets PLOS ONE's style requirements, including those for file naming. The PLOS ONE style templates can be found at

https://journals.plos.org/plosone/s/file?id=wjVg/PLOSOne_formatting_sample_main_body.pdf and

https://journals.plos.org/plosone/s/file?id=ba62/PLOSOne_formatting_sample_title_authors_affiliations.pdf

2. We note that Figure 1 in your submission contain map/satellite images which may be copyrighted. All PLOS content is published under the Creative Commons Attribution License (CC BY 4.0), which means that the manuscript, images, and Supporting Information files will be freely available online, and any third party is permitted to access, download, copy, distribute, and use these materials in any way, even commercially, with proper attribution. For these reasons, we cannot publish previously copyrighted maps or satellite images created using proprietary data, such as Google software (Google Maps, Street View, and Earth). For more information, see our copyright guidelines: http://journals.plos.org/plosone/s/licenses-and-copyright.

We require you to either (1) present written permission from the copyright holder to publish these figures specifically under the CC BY 4.0 license, or (2) remove the figures from your submission:

1. You may seek permission from the original copyright holder of Figure 1 to publish the content specifically under the CC BY 4.0 license.

We recommend that you contact the original copyright holder with the Content Permission Form (http://journals.plos.org/plosone/s/file?id=7c09/content-permission-form.pdf) and the following text:

“I request permission for the open-access journal PLOS ONE to publish XXX under the Creative Commons Attribution License (CCAL) CC BY 4.0 (http://creativecommons.org/licenses/by/4.0/). Please be aware that this license allows unrestricted use and distribution, even commercially, by third parties. Please reply and provide explicit written permission to publish XXX under a CC BY license and complete the attached form.”

Please upload the completed Content Permission Form or other proof of granted permissions as an "Other" file with your submission.

In the figure caption of the copyrighted figure, please include the following text: “Reprinted from [ref] under a CC BY license, with permission from [name of publisher], original copyright [original copyright year].”

2. If you are unable to obtain permission from the original copyright holder to publish these figures under the CC BY 4.0 license or if the copyright holder’s requirements are incompatible with the CC BY 4.0 license, please either i) remove the figure or ii) supply a replacement figure that complies with the CC BY 4.0 license. Please check copyright information on all replacement figures and update the figure caption with source information. If applicable, please specify in the figure caption text when a figure is similar but not identical to the original image and is therefore for illustrative purposes only.

The following resources for replacing copyrighted map figures may be helpful:

USGS National Map Viewer (public domain): http://viewer.nationalmap.gov/viewer/

The Gateway to Astronaut Photography of Earth (public domain): http://eol.jsc.nasa.gov/sseop/clickmap/

Maps at the CIA (public domain): https://www.cia.gov/library/publications/the-world-factbook/index.html and https://www.cia.gov/library/publications/cia-maps-publications/index.html

NASA Earth Observatory (public domain): http://earthobservatory.nasa.gov/

Landsat: http://landsat.visibleearth.nasa.gov/

USGS EROS (Earth Resources Observatory and Science (EROS) Center) (public domain): http://eros.usgs.gov/#

Natural Earth (public domain): http://www.naturalearthdata.com/

3. We note you have included a table to which you do not refer in the text of your manuscript. Please ensure that you refer to Table 5 in your text; if accepted, production will need this reference to link the reader to the Table.

4. If the reviewer comments include a recommendation to cite specific previously published works, please review and evaluate these publications to determine whether they are relevant and should be cited. There is no requirement to cite these works unless the editor has indicated otherwise.

Additional Editor Comments:

Dear Authors,

The reviewers have completed their evaluation of your manuscript. I encourage you to revise and resubmit your work, ensuring that all reviewer comments are thoroughly addressed. Please incorporate the feedback carefully and provide a detailed, point-by-point response that clearly outlines every change made in response to the reviewers’ suggestions.

In addition, kindly correct all typographical and grammatical errors, and ensure that the manuscript is prepared in full compliance with the journal’s formatting and submission guidelines.

We look forward to receiving your revised submission.

[Note: HTML markup is below. Please do not edit.]

Reviewers' comments:

Reviewer's Responses to Questions

**Comments to the Author**

1. Is the manuscript technically sound, and do the data support the conclusions?

The manuscript must describe a technically sound piece of scientific research with data that supports the conclusions. Experiments must have been conducted rigorously, with appropriate controls, replication, and sample sizes. The conclusions must be drawn appropriately based on the data presented.

Reviewer #1: Partly

Reviewer #2: No

Reviewer #3: Partly

Reviewer #4: Partly

**********

2. Has the statistical analysis been performed appropriately and rigorously?

Reviewer #1: No

Reviewer #2: No

Reviewer #3: No

Reviewer #4: No

**********

3. Have the authors made all data underlying the findings in their manuscript fully available?

The PLOS Data policy requires authors to make all data underlying the findings described in their manuscript fully available without restriction, with rare exception (please refer to the Data Availability Statement in the manuscript PDF file). The data should be provided as part of the manuscript or its supporting information, or deposited to a public repository. For example, in addition to summary statistics, the data points behind means, medians and variance measures should be available. If there are restrictions on publicly sharing data—e.g. participant privacy or use of data from a third party—those must be specified.

Reviewer #1: Yes

Reviewer #2: Yes

Reviewer #3: No

Reviewer #4: No

**********

4. Is the manuscript presented in an intelligible fashion and written in standard English?

PLOS ONE does not copyedit accepted manuscripts, so the language in submitted articles must be clear, correct, and unambiguous. Any typographical or grammatical errors should be corrected at revision, so please note any specific errors here.

Reviewer #1: No

Reviewer #2: Yes

Reviewer #3: Yes

Reviewer #4: No

**********

5. Review Comments to the Author

Please use the space provided to explain your answers to the questions above. You may also include additional comments for the author, including concerns about dual publication, research ethics, or publication ethics. (Please upload your review as an attachment if it exceeds 20,000 characters)

Reviewer #1: Dear authors:

Your research on ticks associated with domestic animals in Nepal offers valuable insights into the study of the parasite-host relationship. However, I have several questions regarding the results, particularly the taxonomic identification of the ticks.

Firstly, as you mention in the text, determining the species of some of the ticks was not possible due to their stage of development (i.e., they were nymphs). Two genera (Hyalomma and Dermacentor) could not be specifically identified due to the absence of applicable keys. However, some species that are widely distributed in Nepal, such as Dermacentor everestianus, have recently been redescribed in all their developmental stages. While nymphs can be identified using taxonomic criteria, the characteristics required for identification are more complex to observe under microscopic examination.

2) With regard to this matter, you indicate in the text that several nymphs were found in your study, but you are interested in calculating the sex ratio. Please clarify the rationale behind calculating the sex ratio for species identified at the genus level, given that the nymph and adult stages correspond to different developmental phases.

Thirdly, you indicated that you were unable to identify some specimens because they were engorged females. This is not a valid reason for failing to identify a tick specimen. The characteristics can be observed, and there are methods to manipulate ticks so that they can be observed.

4) The term "spp." is used in the genera that were identified. The term "spp." indicates the presence of multiple morphospecies of ticks within a particular genus. Did you find that to be the case? If not, please use the word "sp."

5) You indicate that your work does not necessitate an ethics statement, however, the guidelines for authors stipulate that the use of vertebrates requires this statement. As you have experience working with domestic animals, please indicate in the text whether the animals came from a herd managed by shepherds or from your university's facilities. In either case, your institution must have a Bioethics Committee that should have approved your work in advance.

6) Please indicate in which scientific collection you plan to deposit the material from this work. Please specify this in the text.

7) Each photograph in Figure 2 must be accompanied by a scale bar. It is unclear whether the quality was compromised during the conversion process, but both the map and Figure 2 do not meet the journal's standards.

Please consider these comments as opportunities for improvement in your future work. Please refer to the attached PDF for additional comments.

Best wishes for a successful outcome.

Reviewer #2: Ethical concerns: Are the studies approved by Ethical Committee of the country? If yes, please provide the details about ethics.

I think the country has veterinary ethical body regarding the study ethical clearance and authors should state if so.

Abstract: Total animals = 210

The sample size for whole of the study areas at provincial level is very low.

66 Ectoparasites constitute a significant problem in most developing and underdeveloped countries

Which sentence/science/citation support this sentence?

114 buffalo, cattle, sheep, goats, and dogs. The study was carried out from September 2023 to January

To collect so few samples, you are almost using two seasons; September last of rainy season and January, the winter season. How can you relate the results over the different periods?

122 udder, back, and inside the tuft of the tail. Approximately 2 to 5 ticks were collected from each

You said you collected about 2 to 5 ticks only, it means, you are leaving other ticks.

140 Shannon-Wiener diversity index (H) = - Σ pi ln(pi)

If you are collecting 2 to 5 ticks only, then, how can H represents accurate values?

142 Simpson’s diversity index (S)= Σ n(n-1)/N(N-1)

Also the S values, how can it be assessed in each host?

148 A total of 755 ticks were collected from 210 animals in Bagmati Province.

It means, the numbers of ticks from 210 animals is very very low in this Province. I think the data will not be so much significant for the global readers.

However, the low data is highly significant in the context of taxonomic data.

173 (Haemaphysalis longicornis, Haemaphysalis sulcata, Haemaphysalis leachi, Haemaphysalis

174 spp.), Dermacentor spp., and Hyalomma spp. During identification, the main morphological

What does it mean by Hyalomma spp. and Haemaphysalis spp.?

181 Haemaphysalis leachi 2.05%, Dermacentor spp. 1.77%, Rhipicephalus spp. 1.36

Similar for Dermacentor spp. and Rhipicephalus spp.?

It is difficult to explain rather than describe different species diversities, abundance based on a few samples of ticks collected.

Therefore, due to low sample collections, although the species identification needs further revision of the MS, especially regarding shortening of the MS. Also authors may make it significant in the context of pathology and explain why different organs/tissues are preferably infested by ticks. However, due to collection of equal/unequal numbers of ticks from few hosts, may make the wrong conclusions.

Thank you.

Reviewer #3: Comment 1

Line no 210 Bagmati province (13 species), But in table it is 11 which one is correct

Comment 2

Actual species is 7. Other are spp. How the index calculated it is misleading.

The spp is not identified and cannot be included in analysis as it is considering as separate species.

Comment 3.

Why the study is in winter, and author himself stated unfavorable.

Comment: 4

What is sampling method. How many domestic animals were available, how many screened, and how many tick infested.

There should be table for Tick infestation rate.

Comment 5:

There is no statistical analysis. For scientific publication there is need of statistical analysis for tick infestation rate, tick abundance, and tick intensity. This will give actual burden of the ticks in the study area.

Comment 6

The Shannon Wiener diversity index

was higher for small ruminants (1.01), followed by dogs (0.877), and large ruminants (0.463)

(Table 6). This showed more diversity of ticks among small ruminants and less diversity in large ruminants.

Wrong interpretation of index. Check reference.

Comment.7

Of the identified species, how many are reported as vectors (zoonosis) transmit to human and domestic animals in Nepal. Give emphasis on this aspects,

Comment 8

How Rhipicephalus decolaratus has been identified. It is very rare and difficult for identification .

Author can avoid sentence such as difficult to identify female and nymph. In the discussion that part of paragraph has to removed and it is common issue and it is not new research finding. This is species diversity paper and experienced taxonomist able to identify all stages of ticks.

Line 292 to 301 statement is not true. Any statement should be given wit published refererence. It will mislead to the reader.

Comment: 9

Constraints of the study

Should be modified that the author itself not confidence.

Comment 10

Line no 207. Total abundance of genera column is misleading. It has to be removed,

Overall comment

It is basic work. Many statements are assumption rather than evidence.

Reviewer #4: Dear author,

Please find below the comments for this article.

PLOS ONE (PONE-D-25-27830)

Species diversity and geographical distribution of ticks infesting domestic animals in Bagmati province, Nepal

Comments

1.Mention in details the yearwise Tick borne diseases recorded in your area or the place of survey?

2.When this study was conducted season? and duration

3.Ethical clearance certificate?

4.Have you got field permission in the individual places?

5.Line 103 On what basis you selected the areas of collection six different sampling areas and

please mention the number of Tick borne infection noticed in those areas

6.Please mention the areas name individually with the elevation in different areas.?

7.Mention any urban rural, semi urban and forested areas were included

8.Can you relate the available species as a vector of any TBD

9.line no. 86 Nepal, economic loss due to ticks and tick-borne diseases in livestock was reported to be 18.71%- Please mention that in detail.

10.line 87 of the total cost [18] ?.

11.128,129 you followed a wet method or dry method for observation of ticks under the microscope?

12.Have you collected ticks from domestic animals, fowls and cats?

13.What are the slide preparation procedures followed?

14.Mention indices references?

15.153 How did you find the age of the individual animal?

16.What is the criteria followed to delineate the less, moderate and high?- any reference?

17.177 Can you mention the specific animal Which is mostly affected by ticks infestation?

18.186,189, affected means Is there any clinical manifestation found on the animal?

19.201 Hill high - what is the elevation?

20.241 Is there any Tick borne disease noticed on goats in those areas?

256,257 highlighti

21.257 its significant role in the transmission of important pathogens in Central Nepal-m please explain in detail.

22.What are the different diseases transmitted by this species in Nepal -Mention it with proper reference?

23.In the discussion please mention about the diseases transmitted by ticks in Nepal reported elsewhere and please mention about the ticks species already reported in Nepal as a small introduction.

24.238 Compare this study with the other studies in Nepal, for example:Utsav Lamichhane and Hom Bahadur Basnet, 2020.,,,,,,,,,,,,,,,,,,,,,,,,,,

25.How is this work similar to Kunwar 2022?

26.240 Please mention in detail the tick species available during the favourable season from these sampled areas.

27.241 Is there any of the Anaplasma, Babesia, and Theileria infection reported in goats in Nepal?

28.246-248 Please mention the tick borne diseases reported from these areas.

29.257 Its significant role in the transmission of important pathogens in Central Nepal - please mention the reference to substantiate your claim.

30.Have you done any screening to find out the pathogens present in these ticks to prove your statements?

31.264-265 Can you cite any reference to support your claim?

32.After studying tick populations for one or two years from this area during different seasons, you can mention seasonal variation patterns.

33.286 Please mention in detail about the pathogens present in Terai region

34.292-294 What are you going to do with unidentified samples?

35.299-300 Collection method is to be changed please use the standard protocol

36.This study is conducted in a very short period, This should be at least conducted for a year to get a clear picture from this place

37.In figure 1,please mention the name of the individual areas.

38.Ref 18 title should be given in the reference

39.All the references should be modified as per the journal format

40.In this article, no statistical outputs were expressed. The following statistical points are required to include.

41.In this article, GIS Choropleth Maps (empowered by ESRI), as Figure 2, are required to include.

42.In which criteria was age calculation made for the host animals grouping (i.e., grouped into three age groups as <1, 1-3, and > 3), and severity of Tick infestation (i.e., grouped into low, moderate and high)?

**********

6. PLOS authors have the option to publish the peer review history of their article (what does this mean?). If published, this will include your full peer review and any attached files.

If you choose “no”, your identity will remain anonymous but your review may still be made public.

**Do you want your identity to be public for this peer review?** For information about this choice, including consent withdrawal, please see our Privacy Policy.

Reviewer #1: **Yes:** Angel Herrera-Mares

Reviewer #2: No

Reviewer #3: No

Reviewer #4: No

**********

[NOTE: If reviewer comments were submitted as an attachment file, they will be attached to this email and accessible via the submission site. Please log into your account, locate the manuscript record, and check for the action link "View Attachments". If this link does not appear, there are no attachment files.]

While revising your submission, please upload your figure files to the Preflight Analysis and Conversion Engine (PACE) digital diagnostic tool, https://pacev2.apexcovantage.com/. PACE helps ensure that figures meet PLOS requirements. To use PACE, you must first register as a user. Registration is free. Then, login and navigate to the UPLOAD tab, where you will find detailed instructions on how to use the tool. If you encounter any issues or have any questions when using PACE, please email PLOS at figures@plos.org. Please note that Supporting Information files do not need this step.

Attachment Submitted filename: PONE-D-25-27830_reviewer.pdf

Attachment Submitted filename: Ethical concerns.docx

Attachment Submitted filename: plos Comment 1.docx

# Author comment: Author response to Decision Letter 1

**DOI:** 10.1371/journal.pone.0351151.r003

26 Oct 2025

Thank you for your comments. We have addressed each one of them and made necessary corrections too.

Attachment Submitted filename: Response to Reviewers_Final.docx

# Peer review: Decision Letter 1

**DOI:** 10.1371/journal.pone.0351151.r004

**Authors:** Faham Khamesipour

30 Jan 2026

PONE-D-25-27830R1

Species diversity and geographical distribution of ticks infesting domestic animals in Bagmati province, Nepal

PLOS One

Dear Dr. Thapa,

Thank you for submitting your manuscript to PLOS ONE. After careful consideration, we feel that it has merit but does not fully meet PLOS ONE’s publication criteria as it currently stands. Therefore, we invite you to submit a revised version of the manuscript that addresses the points raised during the review process.

==============================

**ACADEMIC EDITOR:**

- I want to express my sincere gratitude for your consideration. I have some reservations regarding the taxonomic identity of the nymphs. The authors must ensure that they are working with a single morphospecies or more. Should they elect to undertake a microscopic review of all nymphs, they will be able to verify this.
  It is imperative to note that the calculation of diversity indices necessitates the accurate identification of species. Because of that, I recommended to the authors that they remove the nymph results from the calculation of indices and focus only on tick species, i.e., those identified with genus and specific epithet.
  For these reasons, I believe that the manuscript requires some minor revisions.

-->==============================

Please submit your revised manuscript by Mar 16 2026 11:59PM. If you will need more time than this to complete your revisions, please reply to this message or contact the journal office at plosone@plos.org. When you're ready to submit your revision, log on to https://www.editorialmanager.com/pone/ and select the 'Submissions Needing Revision' folder to locate your manuscript file.

Please include the following items when submitting your revised manuscript:

- A letter that responds to each point raised by the academic editor and reviewer(s). You should upload this letter as a separate file labeled 'Response to Reviewers'.
- A marked-up copy of your manuscript that highlights changes made to the original version. You should upload this as a separate file labeled 'Revised Manuscript with Track Changes'.
- An unmarked version of your revised paper without tracked changes. You should upload this as a separate file labeled 'Manuscript'.

-->If you would like to make changes to your financial disclosure, please include your updated statement in your cover letter. Guidelines for resubmitting your figure files are available below the reviewer comments at the end of this letter.

If applicable, we recommend that you deposit your laboratory protocols in protocols.io to enhance the reproducibility of your results. Protocols.io assigns your protocol its own identifier (DOI) so that it can be cited independently in the future. For instructions see: https://journals.plos.org/plosone/s/submission-guidelines#loc-laboratory-protocols. Additionally, PLOS ONE offers an option for publishing peer-reviewed Lab Protocol articles, which describe protocols hosted on protocols.io. Read more information on sharing protocols at https://plos.org/protocols?utm_medium=editorial-email&utm_source=authorletters&utm_campaign=protocols.

We look forward to receiving your revised manuscript.

Kind regards,

Faham Khamesipour, Ph.D.

Academic Editor

PLOS One

Journal Requirements:

If the reviewer comments include a recommendation to cite specific previously published works, please review and evaluate these publications to determine whether they are relevant and should be cited. There is no requirement to cite these works unless the editor has indicated otherwise.

Please review your reference list to ensure that it is complete and correct. If you have cited papers that have been retracted, please include the rationale for doing so in the manuscript text, or remove these references and replace them with relevant current references. Any changes to the reference list should be mentioned in the rebuttal letter that accompanies your revised manuscript. If you need to cite a retracted article, indicate the article’s retracted status in the References list and also include a citation and full reference for the retraction notice.

[Note: HTML markup is below. Please do not edit.]

Reviewers' comments:

Reviewer's Responses to Questions

**Comments to the Author**

1. If the authors have adequately addressed your comments raised in a previous round of review and you feel that this manuscript is now acceptable for publication, you may indicate that here to bypass the “Comments to the Author” section, enter your conflict of interest statement in the “Confidential to Editor” section, and submit your "Accept" recommendation.

Reviewer #1: All comments have been addressed

Reviewer #2: (No Response)

Reviewer #3: All comments have been addressed

Reviewer #4: All comments have been addressed

**********

2. Is the manuscript technically sound, and do the data support the conclusions?

The manuscript must describe a technically sound piece of scientific research with data that supports the conclusions. Experiments must have been conducted rigorously, with appropriate controls, replication, and sample sizes. The conclusions must be drawn appropriately based on the data presented.

Reviewer #1: Partly

Reviewer #2: Yes

Reviewer #3: No

Reviewer #4: Yes

**********

3. Has the statistical analysis been performed appropriately and rigorously?

Reviewer #1: No

Reviewer #2: Yes

Reviewer #3: No

Reviewer #4: No

**********

4. Have the authors made all data underlying the findings in their manuscript fully available?

The PLOS Data policy requires authors to make all data underlying the findings described in their manuscript fully available without restriction, with rare exception (please refer to the Data Availability Statement in the manuscript PDF file). The data should be provided as part of the manuscript or its supporting information, or deposited to a public repository. For example, in addition to summary statistics, the data points behind means, medians and variance measures should be available. If there are restrictions on publicly sharing data—e.g. participant privacy or use of data from a third party—those must be specified.

Reviewer #1: Yes

Reviewer #2: Yes

Reviewer #3: No

Reviewer #4: Yes

**********

5. Is the manuscript presented in an intelligible fashion and written in standard English?

PLOS ONE does not copyedit accepted manuscripts, so the language in submitted articles must be clear, correct, and unambiguous. Any typographical or grammatical errors should be corrected at revision, so please note any specific errors here.

Reviewer #1: Yes

Reviewer #2: Yes

Reviewer #3: Yes

Reviewer #4: Yes

**********

6. Review Comments to the Author

Please use the space provided to explain your answers to the questions above. You may also include additional comments for the author, including concerns about dual publication, research ethics, or publication ethics. (Please upload your review as an attachment if it exceeds 20,000 characters)

Reviewer #1: Dear authors:

I would like to thank you for taking my comments into consideration. However, I have some remaining reservations about your use of the "sp." and "spp." terms. It is important to note that the application of these taxonomical terms to species at the individual level is incorrect. Even in the reference provided, the terms are explained at the species level rather than the individual level. Due to the aforementioned points, I have some concerns about the calculation of both Diversity Indices. As you have identified nymphs, and are uncertain as to the number of species present, the values are likely to be understimated. This may result in discussions and conclusions that are not fully accurate.

I am aware that certain financial constraints may be an obstacle to accessing certain facilities, such as SEM, which are necessary for the diagnostic characteristics required to identify nymphs at the species level. However, a thorough review of the nymphs under the stereoscopic microscope is recommended to ensure the presence of one or more mophospecies.

I believe this is a vital aspect of your work. One alternative approach is to eliminate the nymphs from your analyses and focus solely on tick species (genus and specific epithet). Please note that I would not advise removing your nymphs results from the manuscript, but rather from the Shannon and Simpson Indices.

Best wishes

Reviewer #2: 1. You have shown the difference between spp. and sp.Thank you. It means there are more than 11 species you discovered. Please explain accordingly.

2. You indicated different diversities of ticks geographically. Why and how does those diversities occur in the geographies? You calculated those values. But what is the significance of those values? What is the current situation of such diversity? Need to be discussed and explained in the MS. Does this depend on host numbers or host age/sexes?

3. You said: '' This might be due to the greater number of

320 domestic hosts involved in the study in Terai under favorable environmental conditions.

How can you evidently say greater number of domestic hosts invovled in the study of Terai under favorable environmental conditions

4. Again, please include the new updates of conclusion. Ticks are very neglected arthropods in the Asia especially, Nepal and neighboring countries.

5. Table 5. Geographical distribution of ticks in different locations

You collected tick specimen and pooled. It may result in the collection and pooling values biased. How did you reduce or nullify the biasness. That is why at your last paragraph, you should clearly state that this is biased study, however, it is qualitative because ..........................

You should write in the legend the total number of hosts studied in each geographic site.

6. Table 6

Please add numbers of hosts studied in the legend. Also in hte province, district, host-wise

7. Table 3

How did you achieve the abundance? what is the number for each species?

8. Table 1:

≤ 1yr 1–3 yrs. ≥3yrs

I did not understand your grouping. In which basis did you do that? any scientiific basis?

less than or equal to 1 year in one group

one to 3 year animal means 1 year is already in previous group.

equal to 3 or more than 3 means 3 is already included in the later group.

How is it possible?

Others:

Most of the references are not properly written. Please sort out this if possible.

Reviewer #3: The reply to the reviewer is not satisfactory. We did not recommend this study in the plos level journal. Sample size is small.

Reviewer #4: This is a well written manuscript on tick diversity & distribution in Bagmati, Nepal. The study is well structured, methods are clearly described and results are presented. Some suggestion for improvement, from minor correction to more substantive recommendation for strengthening the discuss and clarity.

Abstract and summary: the abstract mention Rhipicephalus parva (0.14%), but in table 3 it appears as Rhipicephalus parva (14%)-this is typo that must be corrected

Summary could be slightly expanded to explicitly state the zoonotic implications and public health relevance.

Ensure italicization of all genus/species names throughout (Rhipicephalus, Haemaphysalis, etc.).

In Table 6, ensure column headings are clear (“Large Ruminants”, “Small Ruminants”, “Dogs”).

Reference formatting: ensure all in-text citations match the reference list (not provided here)

Line no 26–27 Specify that Rhipicephalus (Boophilus) microplus is a hard tick for clarity.

Line no 35–36 Correct “Rhipicephalus parva (14%)” to “Rhipicephalus parva (0.14%)” to match Table 3.

Line no 138–139 Clarify: “spp.” for multiple species under a genus, “sp.” for a single unidentified species.

Line no 163–164 Cite reference for infestation severity categories or justify your classification.

Line no 176–178 Specify that nymphs were excluded from sex ratio calculations.

Line no 201–206 Consider adding a brief sentence on why R. microplus might be so abundant (e.g., host availability, climate suitability).

Line no 252 Check consistency in spelling: “Dermacentor” (not Dermacenter).

Line no 289 Change “significant role” to “potential role” unless supported by pathogen detection data.

Line no 313-314 Explicitly state that winter sampling may underestimate tick diversity.

Line no 326–332 This paragraph is important—consider moving it to the Constraints section for better flow.

Line no 344–347 Expand slightly: e.g., Molecular confirmation - species identification accuracy.

Line no 350–355 In conclusions, explicitly recommend integrated tick management in high-diversity areas like Chitwan.

Overall recommendation- Suitable for publication after revisions. The study delivers useful baseline data on tick ecology in Nepal and identifies high-risk areas and hosts. Expanding the discussion on zoonotic risks and control would enhance its significance.

**********

7. PLOS authors have the option to publish the peer review history of their article (what does this mean?). If published, this will include your full peer review and any attached files.

If you choose “no”, your identity will remain anonymous but your review may still be made public.

**Do you want your identity to be public for this peer review?** For information about this choice, including consent withdrawal, please see our Privacy Policy.

Reviewer #1: No

Reviewer #2: **Yes:** Tirth Raj Ghimire

Reviewer #3: No

Reviewer #4: No

**********

[NOTE: If reviewer comments were submitted as an attachment file, they will be attached to this email and accessible via the submission site. Please log into your account, locate the manuscript record, and check for the action link "View Attachments". If this link does not appear, there are no attachment files.]

To ensure your figures meet our technical requirements, please review our figure guidelines: https://journals.plos.org/plosone/s/figures

You may also use PLOS’s free figure tool, NAAS, to help you prepare publication quality figures: https://journals.plos.org/plosone/s/figures#loc-tools-for-figure-preparation.

NAAS will assess whether your figures meet our technical requirements by comparing each figure against our figure specifications.

Attachment Submitted filename: comments 121.2026.docx

# Author comment: Author response to Decision Letter 2

**DOI:** 10.1371/journal.pone.0351151.r005

13 Mar 2026

Response to the editor and reviewers

PONE-D-25-27830R1

Species diversity and geographical distribution of ticks infesting domestic animals in Bagmati province, Nepal

ACADEMIC EDITOR:

I want to express my sincere gratitude for your consideration. I have some reservations regarding the taxonomic identity of the nymphs. The authors must ensure that they are working with a single morphospecies or more. Should they elect to undertake a microscopic review of all nymphs, they will be able to verify this.

It is imperative to note that the calculation of diversity indices necessitates the accurate identification of species. Because of that, I recommended to the authors that they remove the nymph results from the calculation of indices and focus only on tick species, i.e., those identified with genus and specific epithet.

For these reasons, I believe that the manuscript requires some minor revisions.

Thank you for your suggestions. We could not perform taxonomic identification of nymphs so, we have not included nymph for the diversity indices calculation. Also, Rhipicephalus spp. and Haemaphysalis spp. had more than one morphospecies of ticks so, we removed them from the diversity index calculation.

Please submit your revised manuscript by Mar 16 2026 11:59PM. If you will need more time than this to complete your revisions, please reply to this message or contact the journal office at plosone@plos.org. When you're ready to submit your revision, log on to https://www.editorialmanager.com/pone/ and select the 'Submissions Needing Revision' folder to locate your manuscript file.

Please include the following items when submitting your revised manuscript:

A letter that responds to each point raised by the academic editor and reviewer(s). You should upload this letter as a separate file labeled 'Response to Reviewers'.

A marked-up copy of your manuscript that highlights changes made to the original version. You should upload this as a separate file labeled 'Revised Manuscript with Track Changes'.

An unmarked version of your revised paper without tracked changes. You should upload this as a separate file labeled 'Manuscript'.

If you would like to make changes to your financial disclosure, please include your updated statement in your cover letter. Guidelines for resubmitting your figure files are available below the reviewer comments at the end of this letter.

Thank you for giving us the chance to go through our manuscript and make some improvements based on the feedback and suggestions from the editor and reviewers.

Here is our response to reviewer letter where we have tried to go through all the comments and provide point to point response and improve our manuscript.

Bullet points with texts in red in this letter represent our response and the line numbers are included in the parentheses which help to track the changes that we have made in the manuscript.

Changes in the manuscript are also marked with different colors, and we have uploaded both revised version of the manuscript with markings and a clean version of the manuscript.

If applicable, we recommend that you deposit your laboratory protocols in protocols.io to enhance the reproducibility of your results. Protocols.io assigns your protocol its own identifier (DOI) so that it can be cited independently in the future. For instructions see: https://journals.plos.org/plosone/s/submission-guidelines#loc-laboratory-protocols. Additionally, PLOS ONE offers an option for publishing peer-reviewed Lab Protocol articles, which describe protocols hosted on protocols.io. Read more information on sharing protocols at https://plos.org/protocols?utm_medium=editorial-email&utm_source=authorletters&utm_campaign=protocols

Journal Requirements:

If the reviewer comments include a recommendation to cite specific previously published works, please review and evaluate these publications to determine whether they are relevant and should be cited. There is no requirement to cite these works unless the editor has indicated otherwise.

Please review your reference list to ensure that it is complete and correct. If you have cited papers that have been retracted, please include the rationale for doing so in the manuscript text, or remove these references and replace them with relevant current references. Any changes to the reference list should be mentioned in the rebuttal letter that accompanies your revised manuscript. If you need to cite a retracted article, indicate the article’s retracted status in the References list and also include a citation and full reference for the retraction notice.

Thank you so much. All the references have been updated as per the journal’s guidelines.

[Note: HTML markup is below. Please do not edit.]

Reviewers' comments:

Reviewer's Responses to Questions

Comments to the Author

1. If the authors have adequately addressed your comments raised in a previous round of review and you feel that this manuscript is now acceptable for publication, you may indicate that here to bypass the “Comments to the Author” section, enter your conflict of interest statement in the “Confidential to Editor” section, and submit your "Accept" recommendation.

Reviewer #1: All comments have been addressed

Reviewer #2: (No Response)

Reviewer #3: All comments have been addressed

Reviewer #4: All comments have been addressed

2. Is the manuscript technically sound, and do the data support the conclusions?

The manuscript must describe a technically sound piece of scientific research with data that supports the conclusions. Experiments must have been conducted rigorously, with appropriate controls, replication, and sample sizes. The conclusions must be drawn appropriately based on the data presented.

Reviewer #1: Partly

Reviewer #2: Yes

Reviewer #3: No

Reviewer #4: Yes

3. Has the statistical analysis been performed appropriately and rigorously?

Reviewer #1: No

Reviewer #2: Yes

Reviewer #3: No

Reviewer #4: No

4. Have the authors made all data underlying the findings in their manuscript fully available?

The PLOS Data policy requires authors to make all data underlying the findings described in their manuscript fully available without restriction, with rare exception (please refer to the Data Availability Statement in the manuscript PDF file). The data should be provided as part of the manuscript or its supporting information, or deposited to a public repository. For example, in addition to summary statistics, the data points behind means, medians and variance measures should be available. If there are restrictions on publicly sharing data—e.g. participant privacy or use of data from a third party—those must be specified.

Reviewer #1: Yes

Reviewer #2: Yes

Reviewer #3: No

Reviewer #4: Yes

5. Is the manuscript presented in an intelligible fashion and written in standard English?

PLOS ONE does not copyedit accepted manuscripts, so the language in submitted articles must be clear, correct, and unambiguous. Any typographical or grammatical errors should be corrected at revision, so please note any specific errors here.

Reviewer #1: Yes

Reviewer #2: Yes

Reviewer #3: Yes

Reviewer #4: Yes

6. Review Comments to the Author

Please use the space provided to explain your answers to the questions above. You may also include additional comments for the author, including concerns about dual publication, research ethics, or publication ethics. (Please upload your review as an attachment if it exceeds 20,000 characters)

Thank you so much for your time and effort to review and giving us the opportunity to revise the manuscript.

Reviewer #1: Dear authors:

I would like to thank you for taking my comments into consideration. However, I have some remaining reservations about your use of the "sp." and "spp." terms. It is important to note that the application of these taxonomical terms to species at the individual level is incorrect. Even in the reference provided, the terms are explained at the species level rather than the individual level. Due to the aforementioned points, I have some concerns about the calculation of both Diversity Indices. As you have identified nymphs, and are uncertain as to the number of species present, the values are likely to be understimated. This may result in discussions and conclusions that are not fully accurate.

I am aware that certain financial constraints may be an obstacle to accessing certain facilities, such as SEM, which are necessary for the diagnostic characteristics required to identify nymphs at the species level. However, a thorough review of the nymphs under the stereoscopic microscope is recommended to ensure the presence of one or more mophospecies.

I believe this is a vital aspect of your work. One alternative approach is to eliminate the nymphs from your analyses and focus solely on tick species (genus and specific epithet). Please note that I would not advise removing your nymphs results from the manuscript, but rather from the Shannon and Simpson Indices.

Best wishes

Thank you for your valuable comments and suggestions. We have not included nymphs in our diversity indices calculations, and we have included that point in the manuscript as well. (139)

Reviewer #2:

1. You have shown the difference between spp. and sp.Thank you. It means there are more than 11 species you discovered. Please explain accordingly.

Yes. But we have not included them in the calculation. We have not used nypmh data to calculate the diversity indices. Again, few adult ticks also had some missing parts which we discarded. At last, there were some ticks which were not identified upto species level and they could not be categorized as one morphospecies. Hence, we did not use them to calculate our indices. This is one of the major contraints of this study, because to further confirm the ticks upto species level, we could not do the molecular identification due to limited resources.

2. You indicated different diversities of ticks geographically. Why and how does those diversities occur in the geographies? You calculated those values. But what is the significance of those values? What is the current situation of such diversity? Need to be discussed and explained in the MS. Does this depend on host numbers or host age/sexes?

Differences in the diversity of ticks based on geography can be best described based on the climate (temperature, humidity). Since we did sampling in 3 different climatic zone ranging from subtropical to alpine regions as described in line 109-114, difference in diversity was observed.

Higher Shannon Wiener diversity value suggests higher diversity in that geographical region. This suggests that there is presence of multiple species with relatively even distribution and there is higher risk of multiple tick-borne pathogens in that region. Hence, the diversity values are important.

There are limited papers and data on diversity in those areas so we cannot say about the exact current situation right now.

Yes, it does depend on the host related factors like age, sex and numbers. Rather, susceptibility of animals to tick infestation differs from individual to individual based on their age, sex, environment, management practice etc. Young animals may have lower exposure to ticks compared to older ones due to shorter environmental contact. Taking few hosts may underestimate the diversity as less abundant species may not be detected. Different tick species also exhibit host preferences, which can influence the diversity observed in a host population.

3. You said: '' This might be due to the greater number of 320 domestic hosts involved in the study in Terai under favorable environmental conditions.

How can you evidently say greater number of domestic hosts invovled in the study of Terai under favorable environmental conditions

The number of animals used in the study was more from Terai compared to other regions. The line is corrected in the manuscript. (318-319)

4. Again, please include the new updates of conclusion. Ticks are very neglected arthropods in the Asia especially, Nepal and neighboring countries.

Thank you so much. We have updated that in our study. (349-350)

5. Table 5. Geographical distribution of ticks in different locations

You collected tick specimen and pooled. It may result in the collection and pooling values biased. How did you reduce or nullify the biasness. That is why at your last paragraph, you should clearly state that this is biased study, however, it is qualitative

Thank you for your comment. We have already included that in constraints of study

You should write in the legend the total number of hosts studied in each geographic site.Thank you for your suggestion. We have included that information in supplementary file.

6. Table 6

Please add numbers of hosts studied in the legend. Also in hte province, district, host-wise

Thank you. We have included the host information in supplementary file.

7. Table 3

How did you achieve the abundance? what is the number for each species?

Total abundance is the total number of species and abundance percentage was calculated by dividing the total abundance of that species by total number of ticks and multiplying by 100. (149-150)

8. Table 1:

≤ 1yr 1–3 yrs. ≥3yrs

I did not understand your grouping. In which basis did you do that? any scientiific basis?

less than or equal to 1 year in one group

one to 3 year animal means 1 year is already in previous group.

equal to 3 or more than 3 means 3 is already included in the later group.

How is it possible?

Thank you for pointing that out. We divided the groups as <1 yr, 1-3 yrs, and >3 yrs and we have corrected that in our manuscript as well. (174)

Others:

Most of the references are not properly written. Please sort out this if possible.

Thank you. We have corrected that.

Reviewer #3: The reply to the reviewer is not satisfactory. We did not recommend this study in the plos level journal. Sample size is small.

Thank you for your comment. We did check other animals as well but only tick infested animals were included in our study. Since it was mostly conducted during winter season, many of the animals were not infested and we could not include them in our study.

Reviewer #4: This is a well written manuscript on tick diversity & distribution in Bagmati, Nepal. The study is well structured, methods are clearly described and results are presented. Some suggestion for improvement, from minor correction to more substantive recommendation for strengthening the discuss and clarity.

Abstract and summary: the abstract mention Rhipicephalus parva (0.14%), but in table 3 it appears as Rhipicephalus parva (14%)-this is typo that must be corrected

Summary could be slightly expanded to explicitly state the zoonotic implications and public health relevance.

Ensure italicization of all genus/species names throughout (Rhipicephalus, Haemaphysalis, etc.).

In Table 6, ensure column headings are clear (“Large Ruminants”, “Small Ruminants”, “Dogs”).

Reference formatting: ensure all in-text citations match the reference list (not provided here)

Line no 26–27 Specify that Rhipicephalus (Boophilus) microplus is a hard tick for clarity.

Line no 35–36 Correct “Rhipicephalus parva (14%)” to “Rhipicephalus parva (0.14%)” to match Table 3.

Line no 138–139 Clarify: “spp.” for multiple species under a genus, “sp.” for a single unidentified species.

Line no 163–164 Cite reference for infestation severity categories or justify your classification.

Line no 176–178 Specify that nymphs were excluded from sex ratio calculations.

Line no 201–206 Consider adding a brief sentence on why R. microplus might be so abundant (e.g., host availability, climate suitability).

Line no 252 Check consistency in spelling: “Dermacentor” (not Dermacenter).

Line no 289 Change “significant role” to “potential role” unless supported by pathogen detection data.

Line no 313-314 Explicitly state that winter sampling may underestimate tick diversity.

Line no 326–332 This paragraph is important—consider moving it to the Constraints section for better flow.

Line no 344–347 Expand slightly: e.g., Molecular confirmation - species identification accuracy.

Line no 350–355 In conclusions, explicitly recomm

Attachment Submitted filename: Response to reviewers (2).docx

# Peer review: Decision Letter 2

**DOI:** 10.1371/journal.pone.0351151.r006

**Authors:** Faham Khamesipour

3 May 2026

PONE-D-25-27830R2

Species diversity and geographical distribution of ticks infesting domestic animals in Bagmati province, Nepal

PLOS One

Dear Dr. Thapa,

Thank you for submitting your manuscript to PLOS ONE. After careful consideration, we feel that it has merit but does not fully meet PLOS ONE’s publication criteria as it currently stands. Therefore, we invite you to submit a revised version of the manuscript that addresses the points raised during the review process.

==============================

**ACADEMIC EDITOR:**

- While the manuscript has been improved, there are still some concerns about its suitability for PLOS One. I suggest a minor revision at this stage. I would like to express my concerns regarding the failure to identify engorged females. It should be noted that the authors have classified some species under that genera (Rhipicephalus and Haemapysalis). They have expanded their knowledge of tick taxonomy and taxonomy in general, and they have avoided the misuse of the words "spp." and "sp."

-->==============================

Please submit your revised manuscript by Jun 17 2026 11:59PM. If you will need more time than this to complete your revisions, please reply to this message or contact the journal office at plosone@plos.org. When you're ready to submit your revision, log on to https://www.editorialmanager.com/pone/ and select the 'Submissions Needing Revision' folder to locate your manuscript file.

Please include the following items when submitting your revised manuscript:

- A letter that responds to each point raised by the academic editor and reviewer(s). You should upload this letter as a separate file labeled 'Response to Reviewers'.
- A marked-up copy of your manuscript that highlights changes made to the original version. You should upload this as a separate file labeled 'Revised Manuscript with Track Changes'.
- An unmarked version of your revised paper without tracked changes. You should upload this as a separate file labeled 'Manuscript'.

If you would like to make changes to your financial disclosure, please include your updated statement in your cover letter. Guidelines for resubmitting your figure files are available below the reviewer comments at the end of this letter.

If applicable, we recommend that you deposit your laboratory protocols in protocols.io to enhance the reproducibility of your results. Protocols.io assigns your protocol its own identifier (DOI) so that it can be cited independently in the future. For instructions see: https://journals.plos.org/plosone/s/submission-guidelines#loc-laboratory-protocols. Additionally, PLOS ONE offers an option for publishing peer-reviewed Lab Protocol articles, which describe protocols hosted on protocols.io. Read more information on sharing protocols at https://plos.org/protocols?utm_medium=editorial-email&utm_source=authorletters&utm_campaign=protocols.

As the corresponding author, your ORCID iD is verified in the submission system and will appear in the published article. PLOS supports the use of ORCID, and we encourage all coauthors to register for an ORCID iD and use it as well. Please encourage your coauthors to verify their ORCID iD within the submission system before final acceptance, as unverified ORCID iDs will not appear in the published article. *Only* the individual author can complete the verification step; PLOS staff *cannot* verify ORCID iDs on behalf of authors.

We look forward to receiving your revised manuscript.

Kind regards,

Faham Khamesipour, Ph.D.

Academic Editor

PLOS One

Journal Requirements:

If the reviewer comments include a recommendation to cite specific previously published works, please review and evaluate these publications to determine whether they are relevant and should be cited. There is no requirement to cite these works unless the editor has indicated otherwise.

Please review your reference list to ensure that it is complete and correct. If you have cited papers that have been retracted, please include the rationale for doing so in the manuscript text, or remove these references and replace them with relevant current references. Any changes to the reference list should be mentioned in the rebuttal letter that accompanies your revised manuscript. If you need to cite a retracted article, indicate the article’s retracted status in the References list and also include a citation and full reference for the retraction notice.

[Note: HTML markup is below. Please do not edit.]

Reviewers' comments:

Reviewer's Responses to Questions

**Comments to the Author**

1. If the authors have adequately addressed your comments raised in a previous round of review and you feel that this manuscript is now acceptable for publication, you may indicate that here to bypass the “Comments to the Author” section, enter your conflict of interest statement in the “Confidential to Editor” section, and submit your "Accept" recommendation.

Reviewer #1: All comments have been addressed

Reviewer #2: All comments have been addressed

Reviewer #4: All comments have been addressed

**********

2. Is the manuscript technically sound, and do the data support the conclusions?

The manuscript must describe a technically sound piece of scientific research with data that supports the conclusions. Experiments must have been conducted rigorously, with appropriate controls, replication, and sample sizes. The conclusions must be drawn appropriately based on the data presented.

Reviewer #1: Partly

Reviewer #2: Yes

Reviewer #4: Partly

**********

3. Has the statistical analysis been performed appropriately and rigorously?

Reviewer #1: Yes

Reviewer #2: Yes

Reviewer #4: I Don't Know

**********

4. Have the authors made all data underlying the findings in their manuscript fully available?

The PLOS Data policy requires authors to make all data underlying the findings described in their manuscript fully available without restriction, with rare exception (please refer to the Data Availability Statement in the manuscript PDF file). The data should be provided as part of the manuscript or its supporting information, or deposited to a public repository. For example, in addition to summary statistics, the data points behind means, medians and variance measures should be available. If there are restrictions on publicly sharing data—e.g. participant privacy or use of data from a third party—those must be specified.

Reviewer #1: Yes

Reviewer #2: Yes

Reviewer #4: Yes

**********

5. Is the manuscript presented in an intelligible fashion and written in standard English?

PLOS ONE does not copyedit accepted manuscripts, so the language in submitted articles must be clear, correct, and unambiguous. Any typographical or grammatical errors should be corrected at revision, so please note any specific errors here.

Reviewer #1: No

Reviewer #2: Yes

Reviewer #4: (No Response)

**********

6. Review Comments to the Author

Please use the space provided to explain your answers to the questions above. You may also include additional comments for the author, including concerns about dual publication, research ethics, or publication ethics. (Please upload your review as an attachment if it exceeds 20,000 characters)

Reviewer #1: Dear authors:

I appreciate your consideration of my feedback. It seems that your paper has undergone significant improvement. There are a few points that require your attention. I have some concerns about not identifying the engorged females. This is because certain species are suitable for comparison, particularly in the cases of Rhipicephalus and Haemaphysalis. I appreciate your efforts to learn more about tick taxonomy, which will help prevent the misuse of spp. and sp.

-Please note that the words "sp." and "spp." should not be italicized along the manuscript.

-Please direct your attention to lines 125 to 126. These are the results. Please remove the sentence "Approximately 2 to 5 ticks were collected from each animal (20,21)."

-Please note that "ticks" on line 251 should not be italicized.

Best regards and best of luck with your paper.

Reviewer #2: Thank you Authors for your nice works. The article is now beautiful to get published in this journal.

Congratulations!

Reviewer #4: (No Response)

**********

7. PLOS authors have the option to publish the peer review history of their article (what does this mean?). If published, this will include your full peer review and any attached files.

If you choose “no”, your identity will remain anonymous but your review may still be made public.

**Do you want your identity to be public for this peer review?** For information about this choice, including consent withdrawal, please see our Privacy Policy.

Reviewer #1: No

Reviewer #2: No

Reviewer #4: **Yes:** Dr. Renu Govindarajan

**********

[NOTE: If reviewer comments were submitted as an attachment file, they will be attached to this email and accessible via the submission site. Please log into your account, locate the manuscript record, and check for the action link "View Attachments". If this link does not appear, there are no attachment files.]

To ensure your figures meet our technical requirements, please review our figure guidelines: https://journals.plos.org/plosone/s/figures

You may also use PLOS’s free figure tool, NAAS, to help you prepare publication quality figures: https://journals.plos.org/plosone/s/figures#loc-tools-for-figure-preparation.

NAAS will assess whether your figures meet our technical requirements by comparing each figure against our figure specifications.

# Author comment: Author response to Decision Letter 3

**DOI:** 10.1371/journal.pone.0351151.r007

9 May 2026

Thank you for your response. We have made necessary edits to the manuscript.

Attachment Submitted filename: Response to Reviewers .docx

# Peer review: Decision Letter 3

**DOI:** 10.1371/journal.pone.0351151.r008

**Authors:** Faham Khamesipour

24 May 2026

Species diversity and geographical distribution of ticks infesting domestic animals in Bagmati Province, Nepal

PONE-D-25-27830R3

Dear Dr. Thapa,

We’re pleased to inform you that your manuscript has been judged scientifically suitable for publication and will be formally accepted for publication once it meets all outstanding technical requirements.

Within one week, you’ll receive an e-mail detailing the required amendments. When these have been addressed, you’ll receive a formal acceptance letter and your manuscript will be scheduled for publication.

An invoice will be generated when your article is formally accepted. Please note, if your institution has a publishing partnership with PLOS and your article meets the relevant criteria, all or part of your publication costs will be covered. Please make sure your user information is up-to-date by logging into Editorial Manager at Editorial Manager® and clicking the ‘Update My Information' link at the top of the page. For questions related to billing, please contact billing support.

If your institution or institutions have a press office, please notify them about your upcoming paper to help maximize its impact. If they’ll be preparing press materials, please inform our press team as soon as possible -- no later than 48 hours after receiving the formal acceptance. Your manuscript will remain under strict press embargo until 2 pm Eastern Time on the date of publication. For more information, please contact onepress@plos.org.

Kind regards,

Faham Khamesipour, Ph.D.

Academic Editor

PLOS One

Additional Editor Comments (optional):

Reviewers' comments:

Reviewer's Responses to Questions

**Comments to the Author**

1. If the authors have adequately addressed your comments raised in a previous round of review and you feel that this manuscript is now acceptable for publication, you may indicate that here to bypass the “Comments to the Author” section, enter your conflict of interest statement in the “Confidential to Editor” section, and submit your "Accept" recommendation.

Reviewer #1: All comments have been addressed

**********

2. Is the manuscript technically sound, and do the data support the conclusions?

The manuscript must describe a technically sound piece of scientific research with data that supports the conclusions. Experiments must have been conducted rigorously, with appropriate controls, replication, and sample sizes. The conclusions must be drawn appropriately based on the data presented.

Reviewer #1: Yes

**********

3. Has the statistical analysis been performed appropriately and rigorously?

Reviewer #1: Yes

**********

4. Have the authors made all data underlying the findings in their manuscript fully available?

The PLOS Data policy requires authors to make all data underlying the findings described in their manuscript fully available without restriction, with rare exception (please refer to the Data Availability Statement in the manuscript PDF file). The data should be provided as part of the manuscript or its supporting information, or deposited to a public repository. For example, in addition to summary statistics, the data points behind means, medians and variance measures should be available. If there are restrictions on publicly sharing data—e.g. participant privacy or use of data from a third party—those must be specified.

Reviewer #1: Yes

**********

5. Is the manuscript presented in an intelligible fashion and written in standard English?

PLOS ONE does not copyedit accepted manuscripts, so the language in submitted articles must be clear, correct, and unambiguous. Any typographical or grammatical errors should be corrected at revision, so please note any specific errors here.

Reviewer #1: Yes

**********

6. Review Comments to the Author

Please use the space provided to explain your answers to the questions above. You may also include additional comments for the author, including concerns about dual publication, research ethics, or publication ethics. (Please upload your review as an attachment if it exceeds 20,000 characters)

Reviewer #1: (No Response)

**********

7. PLOS authors have the option to publish the peer review history of their article (what does this mean?). If published, this will include your full peer review and any attached files.

If you choose “no”, your identity will remain anonymous but your review may still be made public.

**Do you want your identity to be public for this peer review?** For information about this choice, including consent withdrawal, please see our Privacy Policy.

Reviewer #1: No

**********

# Editor report: Acceptance letter

**DOI:** 10.1371/journal.pone.0351151.r009

**Authors:** Faham Khamesipour

PONE-D-25-27830R3

PLOS One

Dear Dr. Thapa,

I'm pleased to inform you that your manuscript has been deemed suitable for publication in PLOS One. Congratulations! Your manuscript is now being handed over to our production team.

At this stage, our production department will prepare your paper for publication. This includes ensuring the following:

* All references, tables, and figures are properly cited

* All relevant supporting information is included in the manuscript submission,

* There are no issues that prevent the paper from being properly typeset

You will receive further instructions from the production team, including instructions on how to review your proof when it is ready. Please keep in mind that we are working through a large volume of accepted articles, so please give us a few days to review your paper and let you know the next and final steps.

Lastly, if your institution or institutions have a press office, please let them know about your upcoming paper now to help maximize its impact. If they'll be preparing press materials, please inform our press team within the next 48 hours. Your manuscript will remain under strict press embargo until 2 pm Eastern Time on the date of publication. For more information, please contact onepress@plos.org.

You will receive an invoice from PLOS for your publication fee after your manuscript has reached the completed accept phase. If you receive an email requesting payment before acceptance or for any other service, this may be a phishing scheme. Learn how to identify phishing emails and protect your accounts at https://explore.plos.org/phishing.

If we can help with anything else, please email us at customercare@plos.org.

Thank you for submitting your work to PLOS ONE and supporting open access.

Kind regards,

PLOS ONE Editorial Office Staff

on behalf of

Dr. Faham Khamesipour

Academic Editor

PLOS One
